# Anticoagulant residues associated with an attempted rodent eradication from a subtropical coral atoll

**DOI:** 10.1371/journal.pone.0344972

**Published:** 2026-03-23

**Authors:** Carmen C. Antaky, Israel L. Leinbach, Jonathan H. Plissner, Elizabeth N. Flint, Amanda S. Adams, Wesley J. Jolley, Benjamin G. Abbo, Hayden Hamby, Shane R. Siers, Steven C. Hess

**Affiliations:** 1 United States of America Department of Agriculture, Animal and Plant Health Inspection Services, Wildlife Services, National Wildlife Research Center, Hilo, Hawaiʻi, United States of America; 2 United States of America Fish & Wildlife Service, Midway Atoll National Wildlife Refuge, Waipahu, Hawaiʻi, United States of America; 3 United States of America Fish & Wildlife Service, Marine Monuments of the Pacific, Honolulu, Hawaiʻi, United States of America; 4 Island Conservation, Santa Cruz, California, United States of America; 5 United States Department of Agriculture, Animal and Plant Health Inspection Services, Wildlife Services, National Wildlife Research Center, Fort Collins, Colorado, United States of America; Universidade Federal de Minas Gerais, BRAZIL

## Abstract

The use of rodenticides is a primary method for eradicating rodents from islands for conservation purposes. Rodenticide residue monitoring is often incorporated into rodent eradication project planning to understand the potential effects on nontarget species, but robust long-term sampling is often challenging due to logistical and financial constraints. We documented more than two years of rodenticide residues at fine-scale intervals with over 570 samples associated with a rodent eradication attempt. Brodifacoum-25D Conservation was applied in an attempt to eradicate house mice (*Mus musculus*) from Midway Atoll National Wildlife Refuge in the Northwestern Hawaiian Islands. As a cooperating agency, USDA National Wildlife Research Center collected and tested environmental samples for brodifacoum residues, targeting compartments (invertebrates, vertebrates, water, soil, and plants) that may affect the health of humans and wildlife. Brodifacoum residues in invertebrates peaked immediately after bait application and persisted in low levels until becoming undetectable nine months after bait application. Brodifacoum residues decreased over time but persisted in some vertebrate species (geckos, fish, birds) throughout the one-year sampling period after bait applications. All soil and water environmental samples had either no detectable residues or were under method limit of quantitation. No detectable residues were found in drinking water systems or food plant samples. The adaptive environmental monitoring, which included rapid turnaround of analytical chemistry results, enabled real-time management decisions for nontarget species, mitigation approaches, and community action.

## Introduction

Islands across the world are particularly vulnerable to negative impacts from invasive species, especially invasive rodents [1–5]. On many islands, introduced rodents depredate native seabird species that have not evolved with mammalian predators [6–8]. Rodent eradications on islands yield substantial long-term conservation benefits by enabling the recovery of native species and the re-establishment of essential ecosystem processes [9,10]. A common technique to control and eliminate invasive rodents on islands is aerial broadcast of rodenticide across entire islands [11]. The majority of successful eradication attempts have used rodenticides that contain anticoagulants, such as brodifacoum [11,12]. Brodifacoum is a second-generation anticoagulant and requires fewer feedings for a lethal dose to rodents [13]. Nontarget impacts are of concern when applying brodifacoum because all vertebrates are potentially susceptible to the toxicant via primary exposure (direct consumption) and secondary exposure (consumption of organisms that have already consumed the toxicant) [14,15]. Many eradication projects choose to use anticoagulants because there is an antidote available, vitamin K, that can be administered on site by trained staff [16]. Most island eradications using rodenticides analyze nontarget species and environmental samples for anticoagulant residues to assess nontarget risk and mortality [16–18].

**Fig 1:**
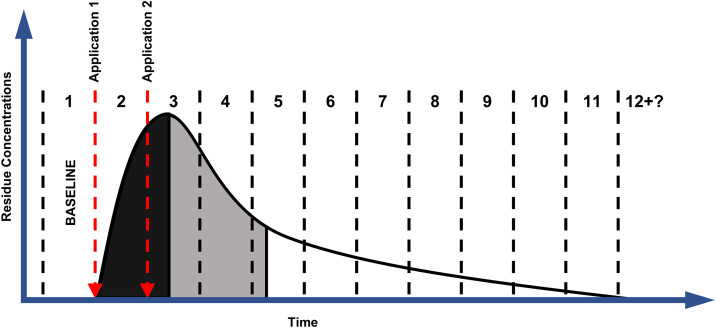
Conceptual biphasic decay of anticipated brodifacoum residues over time. Accumulation during bait applications (black), rapid decline following application peaks as residues are purged from the ecosystem by flushing, metabolism, etc. (gray), and long-term persistence of lower decay rates as bound residues (e.g., in liver tissues) decay naturally (white). Numbers represent sampling intervals over time.

Typically, as brodifacoum bait pellets are applied, the presence of brodifacoum residues throughout the environment increases rapidly and peaks shortly after the last bait application. Following the cessation of bait application and degradation, including some incorporation into the food web, residue levels decline rapidly as brodifacoum percolates into the soil, is degraded by bacterial action, is metabolized by consumers, and is otherwise flushed from the environment by various physical and chemical processes [19–21]. This period of rapid decline is followed by long-term persistence of trace residues that become bound to some environmental compartments (e.g., liver tissues of exposed vertebrates) which break down much more slowly on a time scale more closely associated with the half-life of the compound. This pattern is referred to as ‘biphasic decay’ (Fig 1). Environmental monitoring plans measuring brodifacoum residues attempt to estimate long-term persistence in various environmental compartments, but most are limited in their collection over time [17,22–25]. Remote islands, where most island-wide rodent eradications are implemented, are logistically difficult to access and samples are often only collected for a brief period or with large gaps between collection. To guide nontarget management during rodent eradication projects, greater understanding of how brodifacoum moves through food webs and the expected persistence within environments on a fine timescale may be needed.

Midway Atoll National Wildlife Refuge is home to nearly 6 million nesting seabirds, a small population of the endangered Laysan Duck (*Anas laysanensis*), and is an important migratory stopover location for shorebirds [26]. Midway Atoll hosts the largest colonies for Laysan (*Phoebastria immutabilis*) and Black-footed Albatross (*P. nigripes*) in the world [26]. Since 2015, nesting seabirds have been depredated by introduced house mice (*Mus musculus*) that attack adult albatross incubating eggs or brooding young [26]. These attacks were monitored and increased in number during 2016–2017 throughout Sand Island, one of three islands on Midway Atoll, then decreased to 0–3 attacks per year subsequently [26]. The United States Fish and Wildlife Service (USFWS) attempted to eradicate the *M. musculus* to protect the seabirds of Midway Atoll in July 2023 using the rodenticide Brodifacoum-25D Conservation, a pelleted rodenticide bait containing the active ingredient brodifacoum, intended for conservation purposes. The Brodifacoum-25D Conservation application target rate was 76 kg/ha across two primary and one partial supplemental aerial applications on Sand Island [27].

**Fig 2:**
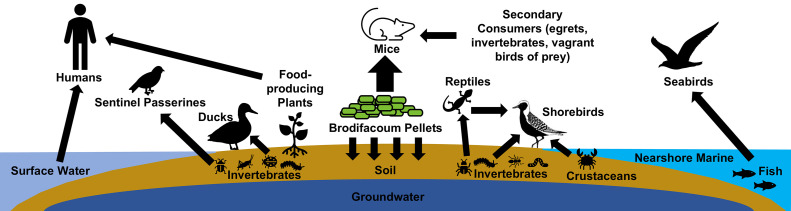
Food web documenting the potential focal pathways of brodifacoum transfer within the environment on Midway Atoll.

The nontarget species of greatest concern was *A. laysanensis*, which was temporarily translocated from Sand to Eastern Island during the eradication effort to minimize brodifacoum exposure [26]. Nontarget mitigation staff monitored ducks and migrating shorebirds throughout and following the eradication project. Although adverse effects to humans from rodent eradication programs have not been documented [28], the project preemptively minimized any potential brodifacoum exposure to humans using multiple measures [26]. We focused monitoring of brodifacoum levels on Sand Island in potential food web pathways of primary or secondary poisoning to *A. laysanensis* and humans (Fig 2). Our intent was to guide management decisions, such as the timing of the *A. laysanensis* release back to Sand Island and reopening the community garden, as well as monitoring the safety of potable water.

The purpose of environmental monitoring was to assess the presence and potential persistence of brodifacoum residues in environmental compartments that may possibly affect the health of humans and wildlife after rodenticide application for all concerned stakeholders. The main objectives of residue monitoring during and after *M. musculus* baiting efforts on Midway Atoll were as follows:

1) Invertebrate Sampling— document the decay of brodifacoum residues from invertebrates (terrestrial arthropods, ghost crabs) known to be constituents of *A. laysanensis* and shorebird diets, to inform nontarget management decisions about the release of captive ducks after rodenticide application;
2) Environmental Sampling— document the accumulation and clearance of brodifacoum residues from various environmental compartments (soil, seawater, freshwater, reptiles);
3) Human Exposure Sampling— evaluate potential brodifacoum exposure to humans (food-producing plants, soil in the community garden, drinking water);
4) Fish Sampling— evaluate brodifacoum exposure to fish from runoff and drift of bait into the nearshore marine environment and secondary exposure to *A. laysanensis* (via mosquitofish [*Gambusia affinis*]); and
5) Carcass Sampling— assess potential mortality of nontarget species resulting from brodifacoum exposure by conducting searches, collections, and chemical analyses of tissues from carcasses.

## Materials and methods

### Field collection methods

Caution was used while collecting samples to avoid cross-contamination by brodifacoum in the environment. Particularly during aerial bait applications, and while bait pellets persisted on the ground, samplers practiced “ultra-clean” methods and replaced sterile gloves for each sample. A baseline and pre-application sample was collected before treatments to establish that there were no pre-existing residues of the analyte in the system prior to the aerial bait application. Samples were collected under Papahānaumokuākea Marine National Monument Conservation and Management Permit (PMNM-2019-005), which authorized field site access by the joint managers of the monument (State of Hawaiʻi, USFWS, and National Oceanic and Atmospheric Administration). All research was reviewed and approved by the United States Department of Agriculture, Animal Plant Health Inspection Services, Wildlife Services, National Wildlife Research Center Institutional Animal Care and Use Committee (QA-3404).

### Invertebrate sampling

The primary purpose of monitoring brodifacoum residues in terrestrial invertebrates was to assess the ongoing risk of brodifacoum poisoning as *A. laysanensis* were being considered for return to Sand Island. Primary exposure (direct consumption of bait pellets and fragments by *A. laysanensis*) was prevented by timing duck release after all pellet material had degraded. Another risk to *A. laysanensis* was secondary exposure through the consumption of insects that fed on brodifacoum bait and contained the toxin. Holthuijzen [29] found that *A. laysanensis* diet comprises multiple arthropod orders but most frequently includes cockroaches (Blattaria), ostracods (Cypridadae), midges (Chironomidae), and isopods (Procellionidae). The invertebrate sample collection targeted these groups and focused on three sites on Sand Island with high duck and shorebird activity. The following sampling types of invertebrates were collected: pooled invertebrates, emerald beetles (*Protaetia pryeri*), cockroaches (*Blattaria* spp.), and pallid ghost crabs (*Ocypode pallidula*). Ghost crabs were collected on this same schedule to assess the risk to shorebirds. Samples were collected using pitfall traps and hand capture. High-resolution low-volume invertebrate samples were stored in small, transparent polyethylene bags (e.g., Whirl-Pak, Bioquip, Rancho Dominguez, CA). Invertebrates were euthanized first by chilling in coolers, and then by freezing.

### Environmental sampling

The following environmental compartments were tested for brodifacoum residues: soil, ocean water, freshwater, and reptiles. The primary goal of the environmental sampling was to assess the uptake of brodifacoum in the environment throughout the project. Soil samples were taken in areas with the highest *A. laysanensis* activity and in areas of high bait application. Water sampling was taken at ocean water and freshwater collection points. Ocean water was collected at shallow-water locations inside the fringing reef. Freshwater was collected for analysis from uncovered seeps as well as any other accessible freshwater sources as feasible throughout the project.

Reptiles, which included non-native geckos (*Lepidodactylus lugubris*, *Hemidactylus frenatus*, and *H. garnotii*), were hand-collected after sunset at three sites on Sand Island and placed in polyethylene bags. Geckos were euthanized by manually applied blunt force trauma followed by decapitation and pithing, an approved Institutional Animal Care and Use Committee method. Reptiles may serve as a pathway of long-term brodifacoum persistence in the environment due to their capability of carrying relatively high sublethal residue burdens and slow metabolic elimination of brodifacoum [30,31].

### Human exposure sampling

The primary goal of human exposure samples was to ensure human health and safety throughout brodifacoum application. Brodifacoum residues were measured within possible human health pathways: food-producing plants, soil, and drinking water. A hydroponic garden and all other gardens/orchards were temporarily deactivated to eliminate food sources for mice, but large fruit trees in orchard and community garden trees persisted. Although the uptake of brodifacoum into these trees was not expected [28,32], testing was conducted out of caution. As part of the eradication project, the three water tanks on Sand Island were filled during the prior rainy season and enclosed so they held sufficient water to supply all island residents and visitors during the project. Water samples collected after bait application helped managers determine when to start re-collecting water for the water tanks.

Fruit from the orchard and community garden, including citrus and papaya, was collected during baseline sampling and at later intervals in coordination with the managers. Soil samples were collected from the community garden and orchard. The community garden and orchard were closed during the project, but the sampling results guided managers in garden reconstruction. To confirm there were no human health threats associated with drinking water, water samples were collected from potable water collection sites in the treatment system on Sand Island.

### Fish sampling

Fish sampling evaluated brodifacoum exposure to marine fish, from drift of bait into the nearshore marine environment, and to mosquitofish, from drift of bait into the freshwater ponds and seeps on Sand Island. Mosquitofish are also known to be a food source for *A. laysanensis* which may increase their risk to secondary exposure. There was little to no risk of brodifacoum exposure to humans via fish on Midway Atoll. Fishing or take of seafood for human consumption within the Papahānaumokuākea Marine National Monument is not permitted except for a limited amount of sustenance fishing of only three species (*Acanthocybium solandri*, *Thunnus albacares*, and *Coryphaena hippurus*). Sustenance fishing was closed during and following baiting operations.

Multiple samples were obtained from the following classes of fish: 1) resident reef fish with highest potential for exposure: *Polydactylus sexfilis* (Pacific threadfin/moi), *Mulloidichthys flavolineatus* (yellowstripe goatfish/weke); 2) small bait fish, fish that are prey items of terns and noddies: *Kuhlia sandvicensis* (Hawaiian flagtail/āholehole) and Mugilidae (mullets/**ʻ**ama**ʻ**ama); and 3) freshwater fish, which includes only one species of mosquitofish (*G. affinis*) present in freshwater ponds.

Marine fish were collected from three main nearshore collection sites. If fish of the correct type were unavailable or unsafe to collect at these sites, alternates were chosen. Freshwater fish (*G. affinis*) were collected from freshwater ponds. Multiple smaller fish (*G. affinis*) and juvenile fish were tested together (i.e., more than one fish per sample) to meet biomass needed for analysis. Fish were collected by cast net, dip net, hook and line, and spear, with sampling efforts restricted to periods of safe weather conditions. Fish were euthanized by manually applied blunt force trauma followed by pithing, an approved Institutional Animal Care and Use Committee method.

### Carcass sampling

Throughout the course of field activities associated with eradication efforts, nontarget organisms (species other than mice, which included *A. laysanensis*, shorebirds, albatross, fish, and non-native birds) found dead were collected and a subset were submitted for chemical residue analysis to assess whether the organism had been exposed to rodenticide intoxication (with birds being the primary taxa of concern). If mass die-offs occurred, one to three fresh carcasses (no degradation observed) were sampled for chemical analysis within each time interval for each focal species.

Carcass transect surveys to address avian mortality (excluding albatross) were performed every two weeks in five areas on Sand Island, targeting shorebird habitat, beginning six days prior to the first bait application and continuing for four months. Each area was approx. 0.015km^2^ and was surveyed by transect lines spaced ten meters apart. All carcasses were collected and removed from the transect areas and a subset of the nontarget carcasses were processed for brodifacoum residue analysis.

### Analytical chemistry

Brodifacoum residues in collected samples were evaluated according to previously published methods [33]. All samples were frozen on Midway Atoll for short term storage and then shipped frozen to the analytical laboratory in Fort Collins, Colorado. Test portions were homogenized prior to analysis according to their sample size and type. Large samples (crustaceans, reptiles, livers, vegetation, fish, and large invertebrate collections) were homogenized using a liquid nitrogen freezer mill. Small invertebrate samples were homogenized by mortar and pestle under liquid nitrogen. Water and soil samples were mixed by stirring and/or shaking with no further comminution. Test portions of the homogenized samples were weighed directly into tubes prior to extraction. The extraction of brodifacoum from each matrix type was conducted according to methods described in LSSU Analytical Services Reports (S1 Appendix). Brodifacoum residues were assayed and quantified by liquid chromatography/tandem mass spectrometry (LC-MS/MS). Detection and quantitation limits were determined by comparing chromatographic noise in a blank control matrix to the peak height of brodifacoum in a fortified control matrix. The detection and quantitation limits are the concentration at which brodifacoum signal is greater than noise by factors of 3 and 10, respectively, and return qualitative (presence/absence) or quantitative values of residue in each matrix type.

## Results

A total of 575 samples were collected and analyzed for brodifacoum residues from one year prior to bait application to one year after bait application with interval periods ranging from two weeks to two months (Table 1). All residue data are provided in Supporting Information (S1 Appendix). Brodifacoum-25D Conservation was applied on Sand Island via aerial broadcast, hand broadcast, and bait stations starting on 1 July 2023. Brodifacoum was applied using pellets with brodifacoum concentration of 25 ppm or 25,000 ng/g. The last aerial broadcast that covered the entire island was on 21 July 2023 and the last hand broadcast was on 5 August 2023. Most bait stations were removed by 17 August 2023 and the last bait stations, roughly a dozen from one area, were removed on 4 September 2023.

**Table 1:** Summary table of environmental monitoring samples by compartment processed for brodifacoum residues over time within dedicated sampling intervals on Midway Atoll, Northwestern Hawaiian Islands, 2022–2024.

Compartment	Baseline	Pre-Application	Post 1	Post 2	Post 3	Post 4	Post 5	Post 6	Post 7	Post 8	Post 9	Post 10
Date Range	July 2022	June 2023	July 2023	Late July/Early Aug. 2023	Late Aug 2023	Sept. 2023	Oct. 2023	Dec. 2023	Feb. 2024	April 2024	June 2024	Aug. 2024
Invertebrates	18	9	18	20	20	24	24	6	6	6	6	12
Crustaceans	6	3	6	6	--	6	6	3	3	3	3	3
Reptiles	6	3	6	6	--	6	6	--	--	--	--	6
Soil	3	6	6	7	--	7	4	--	--	--	--	14
Drinking water	2	2	--	--	--	2	--	--	--	--	--	1
Water collection intakes	2	2	1	1	--	3	2	--	--	--	--	2
Ocean water	4	4	4	4	--	4	--	--	--	--	--	4
Freshwater	4	4	3	4	--	5	--	--	--	--	--	5
Food-producing plants	6	3	--	--	--	6	--	--	--	--	--	3
Fish	22	8	15	16	--	18	18	3	2	2	2	20
Fish carcasses	--	--	2	2	--	--	--	--	--	--	1	--
Duck/shorebird carcasses	2	--	2	1	6	5	2	1	--	1	--	3
Non-native bird carcasses	2	2	4	1	2	1	--	--	1	1	--	1
Seabird carcasses	1	2	2	1	--	2	2	--	--	--	--	2
Contingency	--	--	2	--	--	--	2	--	--	--	--	--
Subtotal	78	48	71	69	28	89	66	13	12	13	12	76

### Invertebrate sampling

#### Pooled invertebrates.

**Fig 3:**
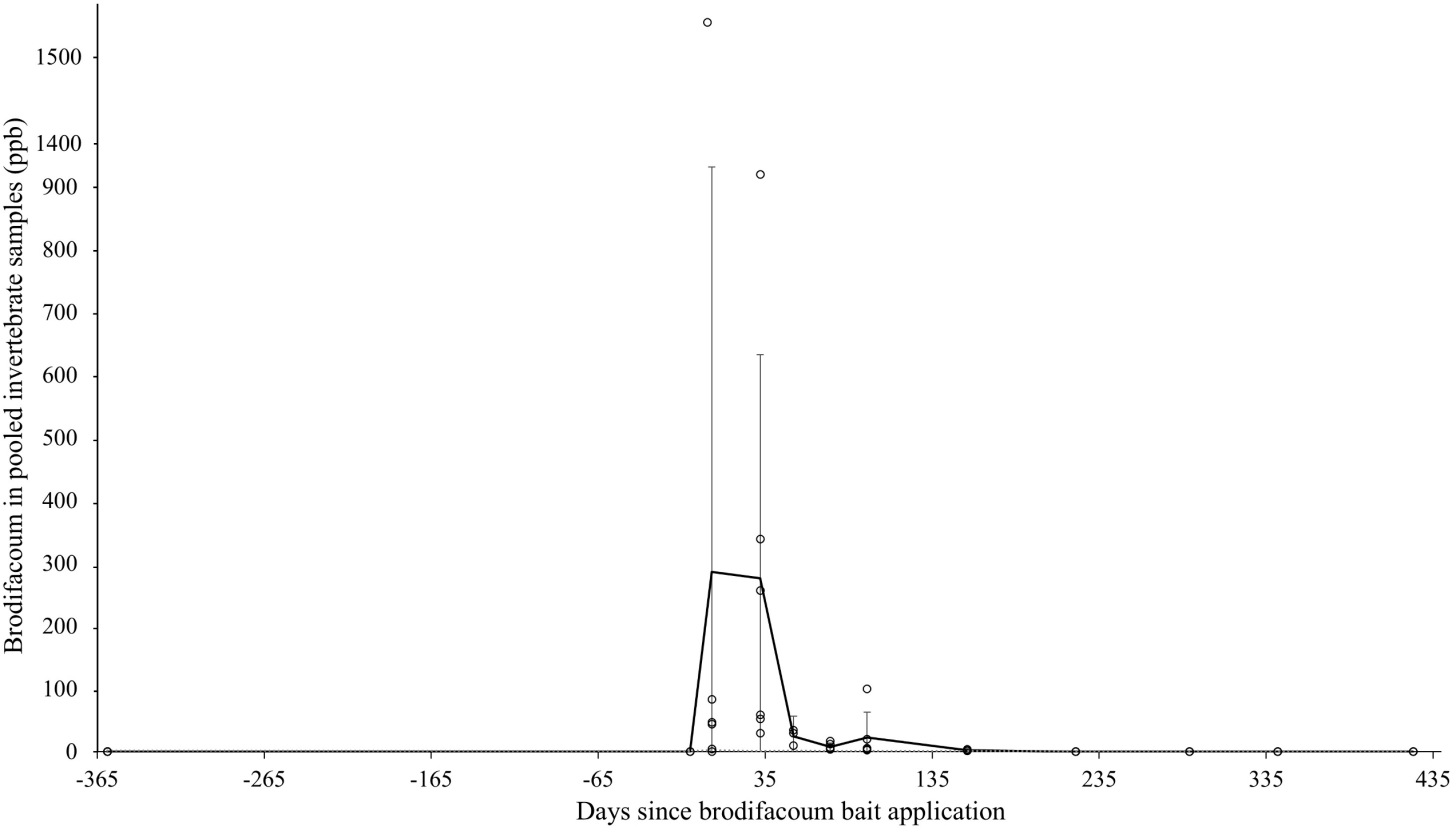
Mean brodifacoum residues in pooled invertebrate samples over time on Midway Atoll, Northwestern Hawaiian Islands, from July 2022 to August 2024. Error bars represent 95% confidence intervals within sample periods. Mean trendline is shown as a bold line. The analytical method limit of detection (range: 0.94–1.90 ng/g) is shown as a dotted line. Y-axis is broken to display high variation in values.

Pooled invertebrate samples, which included a mix of species (Diptera, Amphipoda, Isopoda, etc.) that varied by sample, were systematically collected one year before bait application to one year after bait application at dedicated intervals (Table 1). The highest mean detection of brodifacoum residues in pooled invertebrate samples occurred during the Post 1 interval (286.4 ng/g) (Table 2). Residue levels then decreased over time until becoming undetectable during the Post 7 interval, approximately seven months after the first bait application. Detectable residues followed the predicted gradual and consistent decrease over time (Fig 3).

**Table 2:** Mean brodifacoum residue levels in invertebrates (pooled collection, *Protaetia pryeri*, *Blattaria* spp., and *Ocypode pallidula*) collected systematically on Sand Island, Midway Atoll, Northwestern Hawaiian Islands, 2022–2024. MLOD is method limit of detection; MLOQ is method limit of quantitation; ND is not detected. N/A (not applicable) is listed where there was no brodifacoum detected or only one sample analyzed.

Sample type	Sampling Interval	Mean residue (ng/g)	Standard Deviation	Range	MLOD (ng/g)	MLOQ (ng/g)	# Samples tested	# Samples positive
Pooled	Baseline	ND	N/A	N/A	1.90	6.37	6	0
invertebrates	Pre-App	ND	N/A	N/A	1.20	3.97	3	0
	Post 1	286.37	614.92	<MLOD–1,540.00	2.40	7.98	6	5
	Post 2	276.10	339.72	29.70–920.00	1.20	3.97	6	6
	Post 3	24.41	12.78	9.92–34.10	1.40	4.69	3	3
	Post 4	7.88	5.53	3.90–17.00	1.20	3.97	6	6
	Post 5	22.59	38.49	2.70–100.00	1.20	3.97	6	6
	Post 6	2.33	1.02	1.60–3.50	1.20	3.97	3	3
	Post 7	ND	N/A	N/A	1.20	3.97	3	0
	Post 8	ND	N/A	N/A	1.20	3.97	3	0
	Post 9	ND	N/A	N/A	1.20	3.97	3	0
	Post 10	ND	N/A	N/A	0.94	3.12	3	0
*Blattaria* spp.	Baseline	ND	N/A	N/A	1.90	6.37	6	0
cockroach	Pre-App	ND	N/A	N/A	1.20	3.97	3	0
	Post 1	1,413.17	1,526.82	<MLOD–3,650.00	2.40	7.98	6	4
	Post 2	145.33	134.35	<MLOD–400.00	1.20	3.97	8	6
	Post 3	14.61	13.27	<MLOD–42.10	1.40	4.69	17	16
	Post 4	8.53	6.20	2.20–25.20	1.20	3.97	12	12
	Post 5	3.32	2.29	<MLOD–8.64	1.20	3.97	12	10
	Post 6	2.79	1.63	1.70–4.66	1.20	3.97	3	3
	Post 7	1.90	2.13	<MLOD–4.20	1.20	3.97	3	2
	Post 8	ND	N/A	N/A	1.20	3.97	3	0
	Post 9	ND	N/A	N/A	1.20	3.97	3	0
	Post 10	ND	N/A	N/A	0.94	3.12	3	0
*P. pryeri*	Baseline	ND	N/A	N/A	1.90	6.37	6	0
emerald	Pre-App	ND	N/A	N/A	1.20	3.97	3	0
beetle	Post 1	ND	N/A	N/A	2.40	7.98	6	0
	Post 2	10.72	5.16	3.70–16.80	1.20	3.97	6	6
	Post 4	4.69	3.13	<MLOD–7.87	1.20	3.97	6	5
	Post 5	5.05	3.35	1.80–11.30	1.20	3.97	6	6
	Post 10	ND	N/A	N/A	0.94	3.12	6	0
*O. pallidula*	Baseline	ND	N/A	N/A	1.90	6.37	6	0
pallid ghost	Pre-App	ND	N/A	N/A	0.94	3.12	3	0
crab	Post 1	16.99	17.85	<MLOD–41.10	1.30	4.25	6	4
	Post 2	28.54	17.02	12.70–51.20	0.94	3.12	6	6
	Post 4	9.62	3.22	6.06–15.10	0.94	3.12	6	6
	Post 5	6.99	4.65	2.50–14.90	0.94	3.12	6	6
	Post 6	3.74	3.52	<MLOD–6.99	0.94	3.12	3	2
	Post 7	2.15	3.72	<MLOD–6.50	0.94	3.12	3	1
	Post 8	ND	N/A	N/A	0.94	3.12	3	0
	Post 9	ND	N/A	N/A	0.94	3.12	3	0
	Post 10	ND	N/A	N/A	0.94	3.12	3	0

#### Cockroaches.

Cockroach samples *(Blattaria* spp.) were systematically collected at three main sites, with supplementary samples collected at additional sites of concern (areas of high bait application) on Sand Island. The highest mean detection of brodifacoum residues (1,413.2 ng/g or 1.4 ppm) in cockroach samples occurred during the Post 1 interval (Table 2). Residue levels then decreased over time until becoming undetectable during the Post 8 interval, approximately nine months after the first bait application.

#### Emerald beetles.

Emerald beetle (*P. pryeri*) grub samples were systematically collected at three sites on Sand Island (Table 1). In the one-year post sampling, adult emerald beetles were also sampled. The highest mean brodifacoum residues (10.7 ng/g) in emerald beetle grub samples occurred during the Post 2 interval (Table 2). Emerald beetle grub samples were not collected during the Post 6–9 intervals due to sample prioritization and low risk to *A. laysanensis*. During the Post 10 interval, one year after bait application, no brodifacoum residues were found in both adult and grub emerald beetle samples.

#### Ghost crabs.

**Fig 4:**
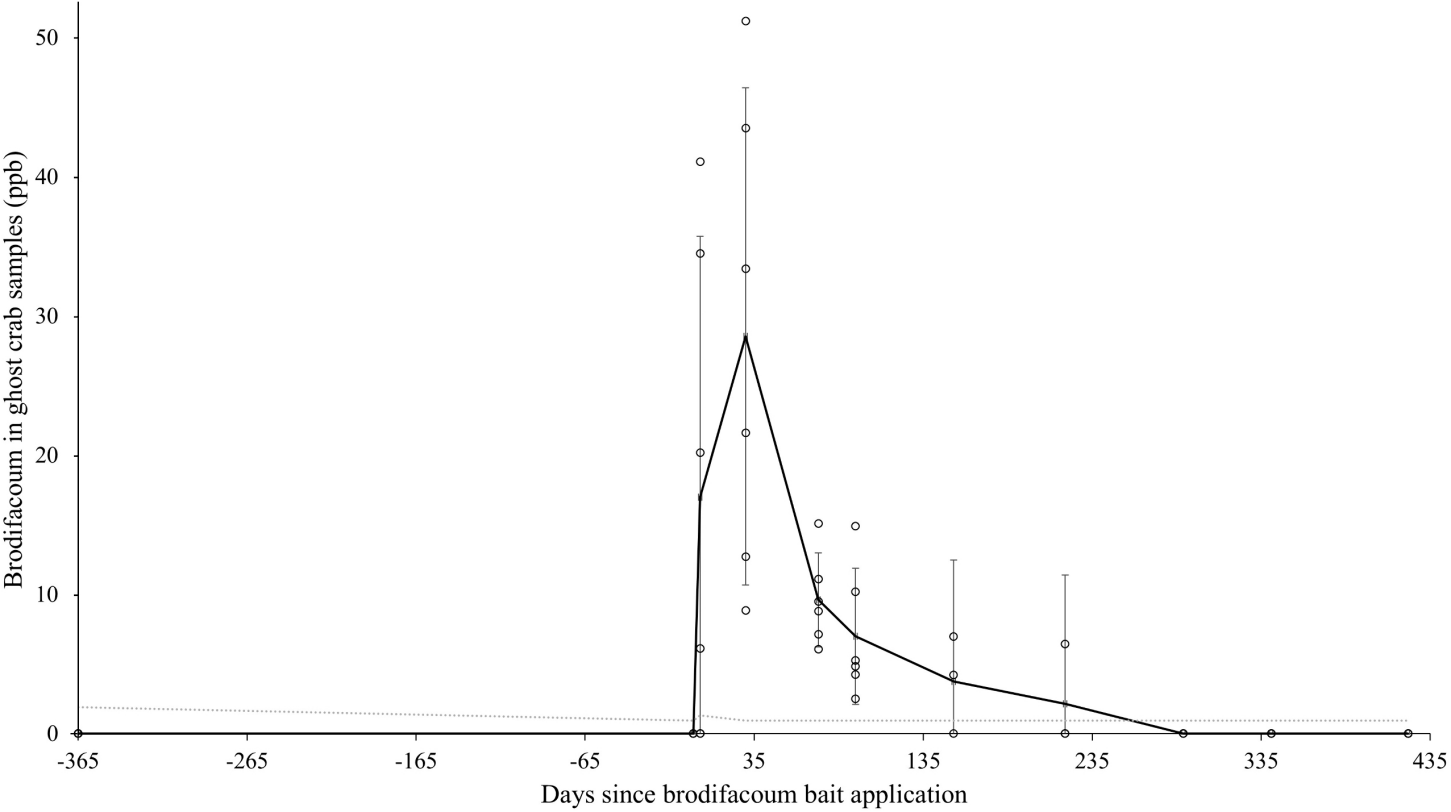
Mean brodifacoum residues with ghost crab (*Ocypode pallidula*) samples over time on Midway Atoll, Northwestern Hawaiian Islands, from July 2022 to August 2024. Error bars represent 95% confidence intervals within sample periods. Mean trendline is shown as a bold line. The analytical method limit of detection (range: 0.94–1.90 ng/g) is shown as a dotted line.

Pallid ghost crabs (*Ocypode pallidula*) were systematically collected at three sites on Sand Island (Table 1). The highest mean brodifacoum residues (28.5 ng/g) in ghost crab samples occurred during the Post 2 interval (Table 2). Residue levels then decreased over time until becoming undetectable during the Post 8 interval, approximately nine months after the first bait application. Detectable residues followed the predicted gradual and consistent decrease over time (Fig 4).

### Environmental sampling

#### Soil (environmental).

Soil samples were systematically collected at four sites on Sand Island (Table 1). All collected samples had no detectable levels of brodifacoum except for two soil samples collected in Post 4 and 5 intervals, both under the MLOQ (Table 3).

**Table 3:** Mean brodifacoum residue levels in the soil, water, reptile, and contingency samples collected systematically on Sand Island, Midway Atoll, Northwestern Hawaiian Islands, 2022–2024. MLOD is method limit of detection; MLOQ is method limit of quantitation; ND is not detected. N/A (not applicable) is listed where there was no brodifacoum detected or only one sample analyzed.

Sample type	Sampling Interval	Mean residue (ng/g)	Standard Deviation	Range	MLOD (ng/g or ng/mL)	MLOQ (ng/g or ng/mL)	# Samples tested	# Samples positive
Soil	Baseline	ND	N/A	N/A	1.90	6.37	2	0
	Pre-App	ND	N/A	N/A	1.30	4.33	3	0
	Post 1	ND	N/A	N/A	1.30	4.33	3	0
	Post 2	ND	N/A	N/A	1.30	4.29	4	0
	Post 4	0.85	1.70	<MLOD–3.40	1.30	4.29	4	1
	Post 5	0.80	1.13	<MLOD–1.60	1.30	4.29	2	1
	Post 10	ND	N/A	N/A	1.30	4.29	10	0
Ocean water	Baseline	ND	N/A	N/A	0.07	0.27	4	0
	Pre-App	ND	N/A	N/A	<0.01	0.01	4	0
	Post 1	ND	N/A	N/A	<0.01	0.01	4	0
	Post 2	ND	N/A	N/A	<0.01	0.01	4	0
	Post 4	ND	N/A	N/A	<0.01	0.01	4	0
	Post 10	ND	N/A	N/A	<0.01	0.01	4	0
Freshwater	Baseline	ND	N/A	N/A	0.10	0.32	4	0
	Pre-App	ND	N/A	N/A	<0.01	0.01	4	0
	Post 1	ND	N/A	N/A	<0.01	0.01	3	0
	Post 2	<0.01	<0.01	<MLOD–0.01	<0.01	0.01	4	2
	Post 4	ND	N/A	N/A	<0.01	0.02	5	0
	Post 10	ND	N/A	N/A	<0.01	0.01	5	0
*Hemidactylus*	Baseline	ND	N/A	N/A	1.90	6.37	5	0
*frenatus*	Pre-App	ND	N/A	N/A	1.00	3.46	3	0
common	Post 1	54.95	75.44	3.52–143.00	1.00	3.46	6	6
house gecko	Post 4	14.30	N/A	N/A	2.00	6.59	1	1
	Post 10	6.71	6.70	<MLOD–13.40	2.00	6.59	3	2
*Hemidactylus*	Post 2	111.95	55.23	72.90–151.00	2.00	6.59	4	4
*garnotii*	Post 4	38.20	16.55	13.40–47.40	2.00	6.59	4	4
Indo-Pacific	Post 5	38.78	6.91	30.80–46.80	2.00	6.59	4	4
gecko	Post 10	ND	N/A	N/A	2.00	6.59	1	0
*Lepidodactylus*	Baseline	ND	N/A	N/A	1.90	6.37	1	0
*lugubris*	Post 2	51.35	43.14	27.30–116.00	2.00	6.59	2	2
mourning	Post 4	57.10	N/A	N/A	2.00	6.59	1	1
gecko	Post 5	31.90	2.55	31.90–35.50	2.00	6.59	2	2
	Post 10	ND	N/A	N/A	2.00	6.59	2	0
Pellet samples	Post 5	17,150.00	7,000.36	12,200.00–22,100.00	2.70	8.95	2	2

#### Water (environmental).

Water was collected from four ocean water and five freshwater sites (Table 1). No detectable levels of brodifacoum were found in ocean water samples (Table 3). The only detectable brodifacoum levels were found in freshwater samples in R2 Ditch and Drainage Pond during the Post 2 interval, both under the MLOQ (Table 3).

#### Reptiles.

Non-native gecko samples were systematically collected at three sites on Sand Island (Table 1). The highest mean brodifacoum residues in geckos was found in the Indo-Pacific gecko (*H. garnotii*) samples during the Post 2 interval (112.0 ng/g; Table 3). Residue levels then decreased over time but were still detectable one year after bait application within common house gecko (*H. frenatus*) samples (Table 3).

#### Contingency samples (environmental).

Decaying moldy bait pellets (two samples of two pellets each) were collected with the Post 5 interval to assess the remaining levels of brodifacoum within the persisting pellets, which were collected on two beach site locations. The bait pellets had mean brodifacoum residues of 17,150.0 ng/g or 17.2 ppm (Table 3).

### Human exposure sampling

#### Food-producing plants.

Food-producing plants were systematically collected at two sites (community garden and orchard) on Sand Island (Table 1). All collected samples had no detectable brodifacoum levels (Table 4).

**Table 4:** Mean brodifacoum residue levels in the plant, soil, and water samples collected systematically on Sand Island, Midway Atoll, Northwestern Hawaiian Islands, 2022–2024. MLOD is method limit of detection; MLOQ is method limit of quantitation; ND is not detected. N/A (not applicable) is listed where there was no brodifacoum detected or only one sample analyzed.

Sample type	Sampling Interval	Mean residue (ng/g)	Standard Deviation	Range	MLOD (ng/g or ng/mL)	MLOQ (ng/g or ng/mL)	# Samples tested	# Samples positive
Plants	Baseline	ND	N/A	N/A	1.90	6.37	6	0
	Pre-App	ND	N/A	N/A	1.10	3.72	3	0
	Post 4	ND	N/A	N/A	1.10	3.72	6	0
	Post 10	ND	N/A	N/A	1.10	3.72	3	0
Soil	Baseline	ND	N/A	N/A	1.90	6.37	1	0
	Pre-App	ND	N/A	N/A	1.30	4.33	3	0
	Post 1	ND	N/A	N/A	1.30	4.33	3	0
	Post 2	ND	N/A	N/A	1.30	4.29	3	0
	Post 4	ND	N/A	N/A	1.30	4.29	3	0
	Post 5	1.10	1.56	<MLOD–2.20	1.30	4.29	2	1
	Post 10	ND	N/A	N/A	1.30	4.29	4	0
Potable water	Baseline	ND	N/A	N/A	0.01	0.32	1	0
	Pre-App	ND	N/A	N/A	<0.01	0.01	2	0
	Post 4	ND	N/A	N/A	<0.01	0.01	2	0
	Post 10	ND	N/A	N/A	<0.01	0.01	1	0
Water	Baseline	ND	N/A	N/A	0.01	0.02	2	0
collection	Pre-App	ND	N/A	N/A	<0.01	0.01	2	0
intakes	Post 1	ND	N/A	N/A	<0.01	0.01	1	0
	Post 2	ND	N/A	N/A	<0.01	0.01	1	0
	Post 4	ND	N/A	N/A	<0.01	0.02	3	0
	Post 5	ND	N/A	N/A	<0.01	0.01	2	0
	Post 10	ND	N/A	N/A	<0.01	0.01	2	0

#### Soil (human exposure).

Soil samples were systematically collected at two sites (community garden and orchard) on Sand Island (Table 1). Soil samples were collected from the base of citrus trees (covered or uncovered with shade cloth). All collected samples had no detectable levels of brodifacoum except for one soil sample (2.20 ng/g) under MLOQ found under an uncovered lime tree in the orchard during the Post 5 interval (Table 4).

#### Water collection and drinking water (human exposure).

Water was sampled at water collection sites throughout the water treatment system and drinking water at water fountains at dedicated intervals (Table 1). All collected samples had no detectable levels of brodifacoum (Table 4).

### Fish sampling

#### Marine fish.

Marine fish were systematically collected at three sites on Sand Island (Table 1). Species collected included Pacific threadfin (*P. sexfilis*), yellowstripe goatfish (*M. flavolineatus*), Hawaiian flagtail (*K. sandvicensis*), bluestriped snapper (*Lutjanus kasmira*), jack (*Caranx* sp.), mullet (*Mugil* sp.), and wrasse (*Thalassoma* spp. and *Bodianus bilumulatus*). Pacific threadfin (*P. sexfilis*), yellowstripe goatfish (*M. flavolineatus*), and Hawaiian flagtail (*K. sandvicensis*) were the most commonly sampled marine fish (Table 5).

**Table 5:** Mean brodifacoum residue levels in fish collected systematically on Sand Island on Midway Atoll, Northwestern Hawaiian Islands, 2022–2024. MLOD is method limit of detection; MLOQ is method limit of quantitation; ND is not detected. N/A (not applicable) is listed where there was no brodifacoum detected or only one sample analyzed.

Sample type	Sampling Interval	Mean residue (ng/g)	Standard Deviation	Range	MLOD (ng/g)	MLOQ (ng/g)	# Samples tested	# Samples positive
*Gambusia affinis*	Baseline	ND	N/A	N/A	1.90	6.37	2	0
western mosquitofish	Pre-App	ND	N/A	N/A	1.20	4.26	2	0
	Post 1	2.95	5.90	<MLOD–11.80	1.30	4.32	4	1
	Post 2	58.55	70.64	<MLOD–146.00	1.20	4.06	4	3
	Post 4	43.09	40.76	4.76–91.20	1.20	4.06	5	5
	Post 5	42.73	28.21	14.70–77.50	1.20	4.06	6	6
	Post 6	17.33	16.56	<MLOD–33.00	1.20	4.06	3	2
	Post 7	28.85	36.27	3.20–54.50	1.20	4.06	2	2
	Post 8	8.00	11.31	<MLOD–16.00	1.20	4.06	2	1
	Post 9	10.25	12.37	1.50–19.00	1.20	4.06	2	2
	Post 10	13.67	13.25	<MLOD–31.70	1.20	4.06	6	4
*Polydactylus sexfilis*	Baseline	ND	N/A	N/A	1.90	6.37	6	0
Pacific threadfin/moi	Pre-App	ND	N/A	N/A	1.20	4.26	2	0
	Post 1	ND	N/A	N/A	1.30	4.32	4	0
	Post 2	0.43	0.85	<MLOD–1.70	1.20	4.06	4	1
	Post 4	0.45	0.90	<MLOD–1.80	1.20	4.06	2	1
	Post 5	ND	N/A	N/A	1.20	4.06	2	0
	Post 10	ND	N/A	N/A	1.20	4.06	5	0
*Kuhlia sandvicensis*	Baseline	ND	N/A	N/A	1.90	6.37	5	0
Hawaiian flagtail/	Pre-App	ND	N/A	N/A	1.20	4.26	3	0
āholehole	Post 1	39.56	37.65	<MLOD–77.40	1.30	4.32	4	3
	Post 2	48.78	24.15	9.18–82.20	1.20	4.06	6	6
	Post 4	16.27	13.22	<MLOD–31.40	1.20	4.06	6	5
	Post 5	11.25	10.23	<MLOD–25.00	1.20	4.06	6	4
	Post 10	ND	N/A	N/A	1.20	4.06	6	0
*Mulloidichthys*	Baseline	ND	N/A	N/A	1.90	6.37	4	0
*flavolineatus*	Pre-App	ND	N/A	N/A	1.20	4.26	1	0
yellowstripe goatfish/	Post 1	0.80	1.60	<MLOD–3.20	1.30	4.32	3	1
weke	Post 2	ND	N/A	N/A	1.20	4.06	2	0
	Post 4	ND	N/A	N/A	1.20	4.06	4	0
	Post 5	ND	N/A	N/A	1.20	4.06	4	0
	Post 10	ND	N/A	N/A	1.20	4.06	1	0
*Lutjanus kasmira*	Baseline	ND	N/A	N/A	1.90	6.37	1	0
bluestriped snapper/ta**ʻ**ape	Post 4	ND	N/A	N/A	1.20	4.06	1	0
*Mugil* sp. mullet/**ʻ**ama**ʻ**ama	Baseline	ND	N/A	N/A	1.90	6.37	1	0
*Caranx* sp. jack/ulua	Baseline	ND	N/A	N/A	1.90	6.37	1	0
*Bodianus bilumulatus* Hawaiian hogfish/**ʻ**a**ʻ**awa	Baseline	ND	N/A	N/A	1.90	6.37	1	0
*Thalassoma duperrey* saddle wrasse/hīnālea lauwili	Post 10	ND	N/A	N/A	1.20	4.06	1	0
*Thalassoma purpureum* surge wrasse/hīnālea hou	Post 10	ND	N/A	N/A	1.20	4.06	1	0

The highest mean brodifacoum residues were found in *K. sandvicensis* (Table 5). Residue levels peaked within *K. sandvicensis* during the Post 2 interval (48.8 ng/g) then decreased over time and was undetectable one year after bait application. *P. sexfilis* had undetectable brodifacoum levels except for one sample in the Post 2 and another sample in the Post 4 interval, both were under MLOQ. *M. flavolineatus* had no detectable levels except for one sample in the Post 1 interval (3.2 ng/g,) which was under the MLOQ. All other alternate species sampled had no detectable levels of brodifacoum. All marine fish samples in the Post 10 interval, one year after bait application, had no detectable brodifacoum residues.

#### Freshwater fish.

**Fig 5:**
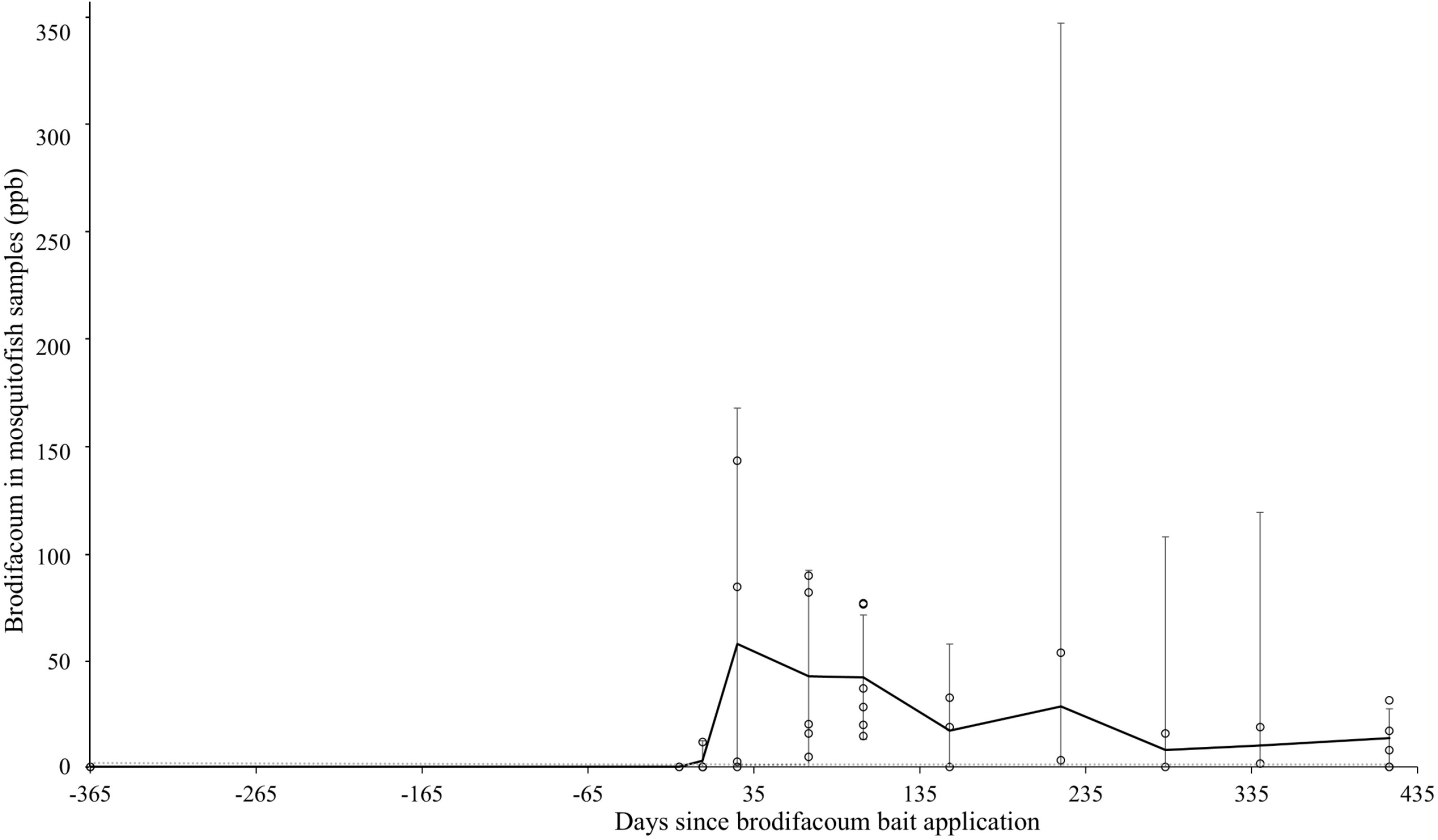
Mean brodifacoum residues with western mosquitofish (*Gambusia affinis*) samples over time on Midway Atoll, Northwestern Hawaiian Islands, from July 2022 to August 2024. Error bars represent 95% confidence intervals within sample periods. Mean trendline is shown as a bold line. The analytical method limit of detection (range: 1.20–1.90 ng/g) is shown as a dotted line.

Mosquitofish (*G. affinis*) were collected at accessible freshwater pond sites on Sand Island at dedicated intervals (Table 1). The highest mean detection of brodifacoum residues in *G. affinis* samples occurred during the Post 2 interval (58.6 ng/g) (Table 5). Residue levels then decreased over time but persisted throughout all sampling intervals. The detectable residues followed the predicted gradual and consistent decrease over time (Fig 5).

### Carcass sampling

#### Seabirds.

Species collected included White Tern (*Gygis alba*), Laysan Albatross (*P. immutabilis*), Red-tailed Tropicbird (*Phaethon rubricauda*), and Bonin Petrel (*Pterodroma hypoleuca*) (Table 1). All seabird carcasses had no detectable brodifacoum levels, except for one *G. alba* carcass collected during the Post 1 interval with a residue level of 2.0 ng/g, under the MLOQ (Table 6). The carcass was from a *G. alba* chick with noticeable mouse bite marks found on the carcass post-mortem.

**Table 6:** Mean brodifacoum residue levels in carcasses collected (shorebirds, seabirds, ducks, egrets, passerines, fish, and invertebrates) on Midway Atoll, Northwestern Hawaiian Islands. Livers were analyzed for all avian samples. MLOD is method limit of detection; MLOQ is method limit of quantitation; ND is not detected. N/A (not applicable) is listed where there was no brodifacoum detected or only one sample analyzed.

Sample type	Sampling Interval	Mean residue (ng/g)	Standard Deviation	Range	MLOD (ng/g)	MLOQ (ng/g)	# Samples tested	# Samples positive
*Gygis alba*	Post 1	2.00	N/A	N/A	1.50	4.97	1	1
White Tern	Post 4	ND	N/A	N/A	1.30	4.26	1	0
	Post 5	ND	N/A	N/A	1.30	4.26	1	0
	Post 10	ND	N/A	N/A	1.30	4.26	1	0
*Phoebastria immutabilis*	Pre-App	ND	N/A	N/A	1.30	4.26	1	0
Laysan Albatross	Post 1	ND	N/A	N/A	1.50	4.97	1	0
	Post 2	ND	N/A	N/A	1.30	4.26	1	0
	Post 10	ND	N/A	N/A	1.30	4.26	1	0
*Phaethon rubricauda* Red-tailed Tropicbird	Post 4	ND	N/A	N/A	1.30	4.26	1	0
*Pterodroma hypoleuca*	Baseline	ND	N/A	N/A	1.90	6.37	1	0
Bonin Petrel	Pre-App	ND	N/A	N/A	1.30	4.26	1	0
	Post 5	ND	N/A	N/A	1.30	4.26	1	0
*Anas laysanesis*	Baseline	ND	N/A	N/A	1.90	6.37	1	0
Laysan Duck	Post 1	1,160.00	N/A	N/A	1.50	4.97	1	1
	Post 2	2,050.00	N/A	N/A	1.30	4.26	1	1
	Post 3	550.60	663.83	81.20–1,020.00	1.50	4.97	3	3
	Post 4	19.35	24.68	1.90–36.80	1.30	4.26	2	2
	Post 8	2.10	N/A	N/A	1.30	4.26	1	1
	Post 10	157.20	170.93	45.80–354.00	1.30	4.26	3	3
*Pluvialis fulva*	Baseline	ND	N/A	N/A	1.90	6.37	1	0
Pacific Golden-Plover	Post 1	468.00	N/A	N/A	1.50	4.97	1	1
	Post 3	744.00	N/A	N/A	1.50	4.97	1	1
	Post 4	1,280.00	N/A	N/A	1.30	4.26	1	1
	Post 5	762.00	379.00	494.00–1,030.00	1.30	4.26	2	2
	Post 6	93.50	N/A	N/A	1.30	4.26	1	1
*Arenaria interpres*	Post 3	886.50	28.99	866.00–907.00	1.50	4.97	2	2
Ruddy Turnstone	Post 4	117.00	N/A	N/A	1.30	4.26	1	1
*Numenius tahitiensis* Bristle-thighed Curlew	Post 4	102.00	N/A	N/A	1.30	4.26	1	1
*Acridotheres tristis*	Baseline	4.70	N/A	N/A	1.90	6.37	1	1
Common Myna	Pre-App	ND	N/A	N/A	1.30	4.26	1	0
	Post 1	803.00	589.73	386.00–1,220.00	1.50	4.97	2	2
	Post 3	684.00	N/A	N/A	1.50	4.97	1	1
	Post 4	961.00	N/A	N/A	1.30	4.26	1	1
	Post 8	226.00	N/A	N/A	1.30	4.26	1	1
*Serinus canaria*	Baseline	ND	N/A	N/A	1.90	6.37	1	0
Island Canary	Pre-App	ND	N/A	N/A	1.30	4.26	1	0
	Post 1	2910.00	N/A	N/A	1.50	4.97	1	1
	Post 7	2.00	N/A	N/A	1.30	4.26	1	1
	Post 10	12.30	N/A	N/A	1.30	4.26	1	1
*Ardea ibis*	Post 1	860.00	N/A	N/A	1.50	4.97	1	1
Western Cattle-Egret	Post 2	937.00	N/A	N/A	1.30	4.26	1	1
	Post 3	910.00	N/A	N/A	1.50	4.97	1	1
*Gambusia affinis*	Post 1	ND	N/A	N/A	1.30	4.32	2	0
western mosquitofish	Post 2	71.00	48.15	36.90–105.00	1.20	4.06	2	2
*Selar crumenophthalmus* bigeye scad/akule	Post 9	ND	N/A	N/A	1.20	4.06	1	0
*Pherecardia striata* lined fireworm	Post 1	ND	N/A	N/A	2.40	7.98	2	0

#### Laysan ducks.

*A. laysanensis* carcasses were collected opportunistically and during dedicated monitoring activities on Sand Island (Table 1). The highest mean detection of brodifacoum residues in *A. laysanensis* samples occurred during the Post 2 interval (2,050.0 ng/g or 2.1 ppm) (Table 6). Residue levels in *A. laysanensis* decreased overall over time but persisted until the last sampling interval.

#### Shorebirds.

Shorebird carcasses were collected on dedicated carcass transects and monitoring activities on Sand Island (Table 1). Species collected included Pacific Golden-Plover (*Pluvialis fulva*), Ruddy Turnstone (*Arenaria interpres*), and Bristle-thighed Curlew (*Numenius tahitiensis*). All shorebirds collected after bait application had detectable levels. The highest mean brodifacoum found in a shorebird was in one *P. fulva* (1,280.0 ng/g or 1.3 ppm) collected in the Post 4 interval on 14 September 2023, almost two and a half months after the first bait application (Table 6). The lowest brodifacoum level (93.5 ng/g) was found in the last shorebird collected, one *P. fulva* on 14 November 2023, almost four and a half months after the first bait application (Table 6). No dead shorebirds were found after November 2024, which may be due to mass migration away from Midway Atoll at that time. Carcass monitoring continued the following year as shorebirds returned (September–November 2024), but no shorebird carcasses were found.

#### Non-native birds.

Non-native bird carcasses were collected on dedicated carcass transects and other monitoring activities on Sand Island (Table 1). Species collected included Common Myna (*Acridotheres tristis*), Island Canary (*Serinus canaria*), and Western Cattle-Egret (*Ardea ibis*). Baseline and Pre-Application samples had no detectable levels of brodifacoum, except for one Common Myna (*A. tristis*), collected one year prior to bait application that had a mean residue level of 4.7 ng/g, which was under the MLOQ (Table 6). All non-native bird samples collected after bait application had detectable brodifacoum levels (Table 6). The highest brodifacoum concentration was found in one *S. canaria* liver sample in the Post 1 interval (2,910 ng/g or 2.9 ppm). Residue levels in non-native birds then decreased over the following sampling intervals.

#### Fish (carcass).

Fish carcasses were collected after fish mortality events were discovered. Mosquitofish (*G. affinis*) associated with a mortality event at R2 Ditch were collected after the first bait application (Post 1 interval). R2 Ditch was covered by a black tarp during the bait application, and when the tarp was removed, *G. affinis* were found dead underneath, presumably due to heat stress but two samples were sent for brodifacoum testing. No detectable brodifacoum residues were found in these samples collected during the Post 1 interval. *G. affinis* were again collected after a mortality event that occurred in the Post 2 interval at the Drainage Pond, which varies in water level and was uncovered during the bait application, and again at R2 Ditch. All samples tested contained brodifacoum residues, with a mean of 71.0 ng/g. Cause of death was inconclusive, as several live *G. affinis* collected had higher brodifacoum residues (77.5–146.0 ng/g). Another mortality event occurred in the Post 9 interval, when multiple bigeye scad (*Selar crumenophthalmus*) were stranded on South Beach, Sand Island. No detectable brodifacoum residues were found in *S. crumenophthalmus* samples.

#### Contingency samples (carcass).

Marine lined fireworm (*Pherecardia striata*) carcasses were collected within the Post 1 interval after several individuals were stranded during a mortality event at Cargo Pier. No detectable levels of brodifacoum were found in the *P. striata* samples (Table 6).

#### Carcass transects.

**Fig 6:**
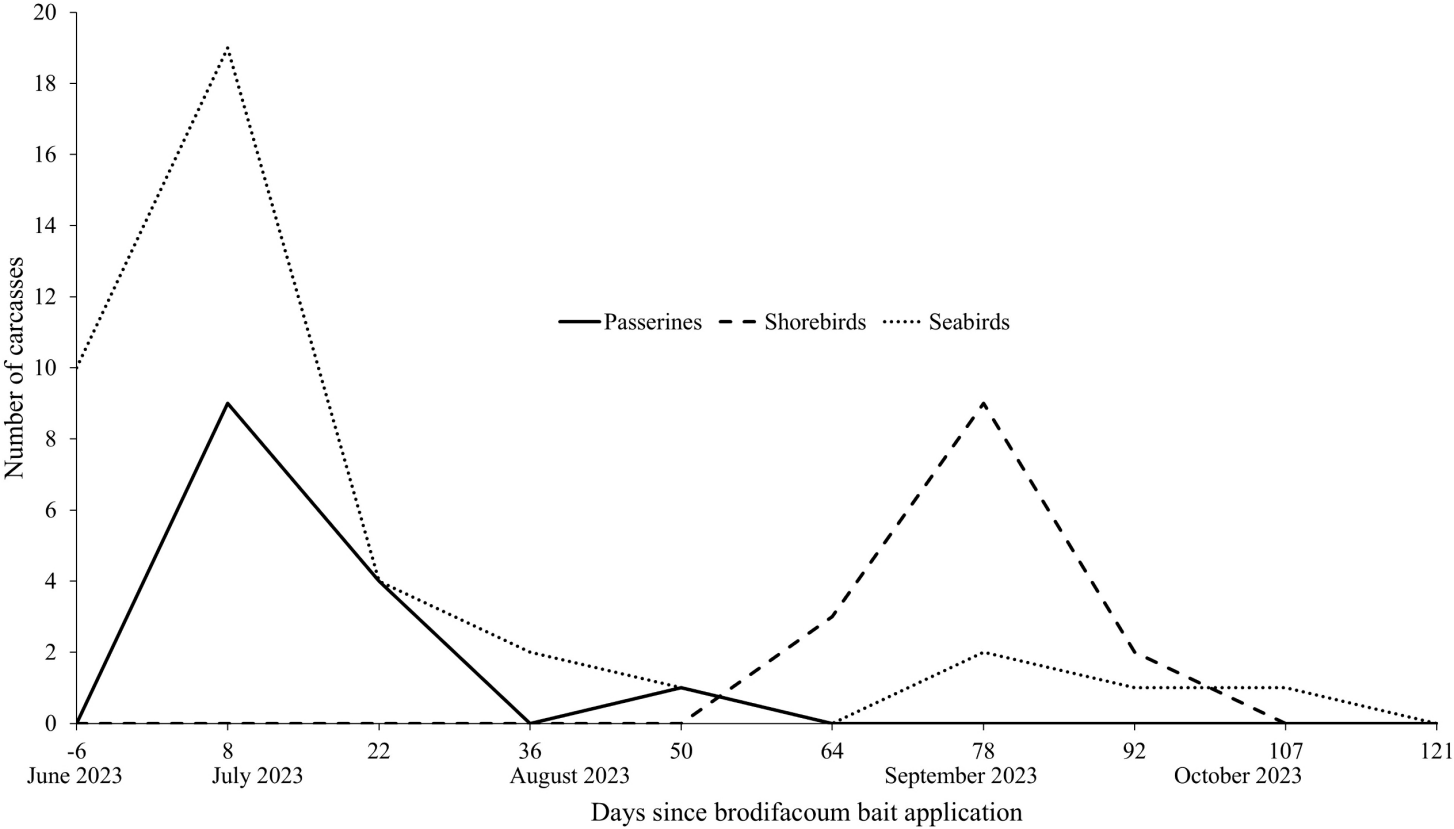
Number of bird carcasses (excluding albatross species) found on standardized carcass transects on Midway Atoll, Northwestern Hawaiian Islands, from June 2023 to October 2023.

All avian carcasses were recorded on dedicated carcass transects except albatross species due to the large number of natural chick mortalities every summer. The majority of passerine carcasses were found within the first month after the first bait application, while shorebirds carcasses were found in higher numbers two months after the first bait application (Fig. 5). Most seabirds found were natural mortalities of Bonin Petrel (*P. hypoleuca*) chicks in June and July (Fig 6).

## Discussion

Due to nontarget species concerns, especially shorebirds and endangered *A. laysanensis* on Midway Atoll during rodenticide bait application, sampling effort was focused on environmental compartments that pose risk of primary and secondary poisoning to nontarget avian species. The environmental monitoring team worked closely in collaboration with nontarget mitigation staff and USFWS managers to adjust timing, sampling type, sampling location, and prioritization of brodifacoum testing to obtain samples of concern. The adaptive environmental monitoring aided real-time management decisions by USFWS for nontarget species, mitigation approaches, and community action.

### Invertebrate sampling

Brodifacoum is not toxic to invertebrates, but invertebrates that consume the brodifacoum bait serve as a pathway of secondary exposure to vertebrate consumers (*A. laysanensis*, shorebirds, etc.) [34]. The main diet items of *M. musculus* on Midway Atoll are invertebrates [35]. Secondary poisoning via invertebrates to target species (mice) may augment eradication goals but also produces a risk to nontarget species (birds). The longest observed persistence of brodifacoum in invertebrate samples was seven months after bait application. The brodifacoum residue levels in the majority of samples tested were very low relative to the bait concentration, with all averages a thousand times less than that of the starting bait concentration two months after bait application.

Following bait application, brodifacoum was found in 124 out of 183 invertebrate samples collected (67.8%). Pooled invertebrate samples, representing a diverse number of arthropod species found in the *A. laysanensis* diet, peaked in brodifacoum residues immediately following the first bait application (Post 1 interval), and steadily declined until no detectable levels were found after seven months from the first bait application (Post 7 interval). Samples were under the MLOQ in December 2023 (five months after bait application), indicating low risk of secondary poisoning from invertebrates. Brodifacoum residues in cockroach samples, an important prey item in the *A. laysanensis* diet [29], peaked at the Post 1 interval as well, then declined rapidly but persisted in low levels until undetectable nine months following the first bait application (Post 8 interval). Ghost crab samples followed a similar trend, peaking after the second aerial bait application, then declined and persisted until seven months from the first bait application (Post 7 interval).

USFWS released *A. laysanensis* back to Sand Island in January 2024, six months after the first bait application. USFWS determined the time to return *A. laysanensis* back to Sand Island based on multiple factors: no observed mortality events in other bird species (sentinel mynas or canaries monitored with VHF transmitters), no pellets observed on the ground, prior safe release of a few VHF monitored *A. laysanensis* individuals, consideration of risk of continued crowded duck population conditions on Eastern Island, and low levels of residues observed within *A. laysanensis* diet samples. Residues within their main invertebrate prey items (pooled invertebrate and cockroach samples) were less than the MLOQ a month before the *A. laysanensis* release back to Sand Island.

### Environmental sampling

Environmental sampling assessed different biotic and abiotic compartments of Midway’s ecosystem to document accumulation and clearance of brodifacoum residues within the environment. Brodifacoum entered the soil and water in very low amounts. Only 2 of 23 (8.7%) soil samples tested after bait application had detectable residues, and both were under the MLOQ and in areas of high bait application. Only 2 of 17 (11.7%) freshwater samples tested post bait application had detectable residues, both under the MLOQ, collected from drainage accumulation areas. No brodifacoum was detected entering the ocean water. Undetectable levels in ocean water are likely due to the high dilution and flush rate and well as relative insolubility of brodifacoum in water [36]. The majority (26 of 30; 86.7%) of non-native geckos, that feed on invertebrates and can bioaccumulate the toxin in their livers, had detectable residues (above MLOQ) after bait application. All decaying pellet samples tested in the Post 5 interval, three months after bait application, contained high brodifacoum levels (mean 17.2 ppm). Following this result, USFWS made the remaining pellets on the beach inaccessible to foraging shorebirds.

### Human exposure sampling

All samples tested for potential human exposure (plants, soil, drinking water, and water collection intakes) had no detectable levels of brodifacoum, except for one soil sample under MLOQ, 1 of 33 (3.0%). These results demonstrate that brodifacoum applications performed on Midway Atoll had low risk to human health which is consistent with other island rodent eradication attempts using rodenticides [21,28]. Following the results of continued no detectable brodifacoum levels in the water collection system in October 2023, USFWS started collecting water again to refill the water tanks for island residents. Additionally, following the results of no detectable levels in food-producing papaya and lime plants, island residents were allowed to collect fruits and plants for consumption at the community garden.

### Fish sampling

The marine fish sampling evaluated brodifacoum exposure to fish from runoff and drift of bait into the nearshore marine environment. Only 21 of 62 (33.9%) marine fish tested after bait application had detectable residues and no detectable residues were found one year after bait application. These findings indicate low exposure to reef fish and bioaccumulation of brodifacoum within the food chain in the nearshore marine environment on Midway Atoll.

Freshwater mosquitofish (*G. affinis*), a minor but known prey item of *A. laysanensis*, assessed secondary exposure. Mean brodifacoum residues in mosquitofish peaked in the Post 2 interval (one month after the first bait application) then decreased but remained over the MLOQ one year following bait application. 26 of 34 (76.5%) mosquitofish samples detected residues after bait application. Mosquitofish presented low but potential secondary exposure risk to *A. laysanensis* during their release back to Sand Island.

### Carcass sampling

Carcass sampling assessed the mortality of nontarget vertebrates resulting from brodifacoum exposure by standardized searches and chemical analysis of tissues from bird and fish carcasses. Second-generation anticoagulants are highly toxic to vertebrates, and especially to birds [37,38]. Avian mortalities are often reported after attempted rodent eradication using brodifacoum [12,14,39–41]. A residue threshold of 250 ng/g brodifacoum is considered lethal exposure for birds [42]; however, it should also be noted that LD50 values are highly variable within species [43,44]. The LD50 for the Mallard Duck (*Anas platyrhynchos*), a surrogate species for the Laysan Duck, is 0.00026 mg/g body weight [45]. At peak brodifacoum concentrations observed in cockroaches (0.00141 mg/g), an adult duck weighing 500 g would need to eat 92.2 g of cockroaches to approach a lethal dose.

No seabird carcasses (*n* = 9) had detectable brodifacoum levels or were over the MLOQ, indicating mortalities due to brodifacoum was unlikely among seabirds after bait application. Seabird carcasses collected on carcass transects indicated only natural moralities (Fig 6). All shorebird carcasses (*n* = 10) tested following bait applications had detectable levels of brodifacoum, and the majority were considered in the lethal range (range: 93.5–1,280.0 ng/g). All shorebirds collected on carcass transects were presumed to have died from brodifacoum poisoning, peaking two months after the first bait application in September 2023 which corresponds to peak migration period (Fig 6). No dead shorebirds were found after November 2023. All non-native passerine and egret bird carcasses (*n* = 11) tested following the bait application until two months after the first bait application had potentially lethal levels of brodifacoum (range: 684.0–2,910.0 ng/g). Non-native bird carcasses collected two months to one year after bait application (*n* = 3) continued to have detectable residues but less than the presumed lethal threshold (range: 2.0–226.0 ng/g). Passerines collected on carcass transects were inferred to have died from brodifacoum poisoning, peaking in July 2023 (within the first month after the first bait application; Fig 6).

All *A. laysanensis* carcasses tested following the bait application until late-August (under two months after the first bait application), had potentially lethal levels of brodifacoum from primary and secondary poisoning (*n* = 5; mean range: 550.6–2,050.0 ng/g). Carcasses tested 2–12 months after the application contained less than presumed lethal levels (*n* = 3; range: 2.1–36.8 ng/g). One anomaly was an adult *A. laysanensis* found in the Post 10 interval, one year after bait application, which had a residue level of brodifacoum of 354.0 ng/g. This duck may have been disproportionately exposed to brodifacoum during the application period as there were multiple adult ducks that swam back to Sand Island that were subsequently captured, administered vitamin K, and translocated back to Eastern Island. Alternatively, brodifacoum persistence in mosquitofish may have served as a secondary poisoning pathway if this bird consumed these vertebrate prey (e.g., [37]). Of the *A. laysanensis* carcasses tested after *A. laysanensis* were brought back to Sand Island, only 1 of 4 carcasses had potentially lethal levels of brodifacoum.

### Comparative/wider context

To our knowledge, this is the most robust (575 samples) and fine-scale documentation of brodifacoum persistence in the environment, with sampling at consistent intervals for one year prior to one year after anticoagulant bait application. Sampling targeted invertebrates, fish, water, and soil for assessing secondary exposure risk to inform the timing of release of endangered *A. laysanensis* back to Sand Island and step-down mitigation measures for shorebirds. The eradication project did not exceed the permitted take of the endangered *A. laysanensis* and there has been no documented significant change in the *A. laysanensis* or shorebird populations on Midway Atoll in the year after the baiting operations. Additionally, the comprehensive sampling provides fine scale knowledge on how brodifacoum moves throughout environmental compartments in a subtropical coral atoll. Brodifacoum residues in invertebrates peaked immediately after bait application and persisted until seven months after bait application. Brodifacoum persisted in vertebrate species (geckos, freshwater fish, birds) for over a year after the first bait application. All soil and water samples (environmental samples) had either no detectable residues or were under MLOQ during the entire sampling period. No detectable residues were found in the drinking water system or plants (human exposure samples) during the entire sampling period.

Comparing the results of rodenticide residues collected in this study to other studies was difficult due to differences in methodology, climate, ecology, and application rates. However, three major projects in the United States using brodifacoum had similar broadcast techniques. A project on Hawadax Island, Alaska, in 2008 used two applications of Brodifacoum 25D: Conservation at a rate of 17 kg/ha [46]. A project on Palmyra Atoll in the Tropical Pacific in 2011 used two applications with a supplemental hand broadcast Brodifacoum 25W: Conservation at a rate of 77.5 kg/ha [24]. A project on Wake Atoll in the tropical Pacific in 2012 used two applications of Brodifacoum 25W: Conservation at a rate of 22.5 kg/ha [25].

On Hawadax Island, monitoring was limited to the collection of water samples within 48 hours of bait application, with no other samples collected until the following Spring due to poor winter weather excluding crews from the island [46]. Brodifacoum residues were not detected in any of the marine water samples and in only 2 of 10 freshwater samples initially collected, and no brodifacoum was detectable in soil and water samples the following Spring, eight months after bait application, which is similar to this study’s findings on Midway Atoll [22,46]. Detectable brodifacoum residues were found in 35 of 36 bird carcasses (range: 27–4,000 ng/g) tested for residues eight months after bait application on Hawadax Island [22]. Brodifacoum residues in liver tissues samples on Hawadax Island were higher overall than those found on Midway Atoll, likely due to nontarget birds scavenging on rat carcasses and lack of extensive nontarget mitigation measures [22].

On Palmyra Atoll, monitoring consisted of sampling water, soil, crabs, terrestrial invertebrates, geckos, fish, and carcasses found, which was similar to Midway Atoll’s environmental monitoring plan but only tested up to two months after the first bait application, with the exception of one bird carcass collected more than seven months after the first bait application [24]. Most animal samples contained brodifacoum residues (84.3%), similar to animal samples collected on Midway Atoll (76.5%) at the same time interval within two months after bait application [24]. Three years later, brodifacoum was tested in mullet, geckos, cockroaches, and crabs, and no detectable residues were found on Palmyra Atoll [47]. On Midway Atoll, marine fish, cockroaches, and crabs had no detectable residues one year after bait application but brodifacoum residues were still present in geckos, freshwater fish, and bird (passerine and duck) carcasses. Continued sampling at two and three years after bait application could determine when brodifacoum residues become undetectable in geckos, freshwater fish, and bird carcasses on Midway Atoll.

On Wake Atoll, monitoring consisted of testing fish and crabs up to three months after bait application [48]. No brodifacoum was documented in eels or crabs, and detectable brodifacoum was found only in 5 of 40 marine fish samples, which included bluefin trevally (*Caranx melampygus*) and blacktail snapper (*L. fulvus*), all within the lagoon [48]. Three years later, 2 of 69 fish samples had detectable brodifacoum residues, all in blacktail snapper (*L. fulvus*) and all within the lagoon on Wake Atoll [25]. On Midway Atoll, a bluestriped snapper (*L. kasmira*), in the same family as *L. fulvus* (Lutjanidae), was tested in the Post 4 interval (two months after bait application). No brodifacoum was found in the bluestriped snapper (*L. kasmira*) sample on Midway Atoll. One year after bait application, none of the 20 marine fish samples tested had detectable brodifacoum residues on Midway Atoll. Overall, potentially due to lower drift of bait into the marine environment and the environmental topography (no semi-enclosed lagoon), less long-term accumulation of brodifacoum occurred in marine fish on Midway Atoll than on Wake Atoll.

## Conclusions

Brodifacoum residues were still detectable in invertebrates, a primary diet item of the endangered *A. laysanensis*, collected up to seven months following the first bait application on Midway Atoll. Future projects may consider including monitoring for toxicant residues in invertebrate prey items of species of concern for at least seven months following rodenticide broadcast. Additionally, vertebrate prey items, like mosquitofish had detectable brodifacoum more than one year after bait application. Thus, the risk of exposure to other organisms may persist for over one year following application of brodifacoum bait. If there is a species of concern that consumes vertebrate prey, it may be appropriate to continue residue testing for at least one year and consider sampling up to three years after brodifacoum bait application [25].

Rodent eradications using brodifacoum must balance the risk of nontarget mortality and residue persistence in the environment with the potential conservation benefits and likelihood of successfully removing the target invasive rodent species [9,10,16,40,49]. Rodent eradication management plans including long-term rodenticide residue monitoring with a collaborative approach (i.e., nontarget mitigation decisions driven by collected environmental residue data) can minimize the potential of secondary exposure to reduce nontarget mortality and enhance risk evaluations [50]. We demonstrated how these data can be used to mitigate nontarget endangered species mortality and to ensure the safety of potable water for human consumption by monitoring and reopening water collection sources after extensive negative test results.

## Supporting information

S1 Appendix All final chemistry reports. (ZIP)
