# Supplementary material for: Anticoagulant residues associated with an attempted rodent eradication from a subtropical coral atoll
Source: PLoS One. 2026 Mar 23;21(3):e0344972. doi: 10.1371/journal.pone.0344972 (PMC13008109; doi:10.1371/journal.pone.0344972)
Supplement: S1 Appendix — (ZIP) [file pone.0344972.s001.zip › Supporting Information S1/22-031 Baseline Midway Island Brodifacoum Report.pdf]

Wildlife Services **NWRC** National Wildlife Research Center<br>Analytical Services Report	United States Department of Agriculture<br>Animal Plant Health Inspection Service<br>Wildlife Services<br>National Wildlife Research Center<br>Laboratory Support Services Unit	Invoice #: 22-031<br>Date: 01/10/23<br>Page: 1 of 15

To: Carmen Antaky
Biologist
NWRC Hawai'i Field Station

Subject: Determination of brodifacoum in various matrices from Midway Island (QA-3404)

Methods: 188A-"Determination of Multiple Rodenticide Residues in Avian Liver by dSPE and LC-MS/MS" -Non-GLP
"Determination of Brodifacoum Residues in Water"-Non-GLP

Analysis Dates: 12/02/22, 12/16/22, 12/20/22, 12/27/22, 12/28/22, 12/30/22

Notebook References: AC165, pp.144-165
QC35, p.68

Analyst: Ben Abbo, Hayden Hamby

Sample Description:

Samples of invertebrates (n=18), fish (n=22), crustaceans (n=6), reptiles (n=6), food producing plants (n=6), soil (n=3), water (n-12) and birds (n=7) were submitted on 08/01/22. See sample descriptions on pp.4-12.

Additional Comments:

- The bird carcasses were dissected, and the livers were taken for sampling.
- Sample S220801-75 was decayed; thus, no liver tissue could be obtained from this sample.
- Sample S220801-80 was lost during the homogenization process when its canister ruptured, causing the loss of the sample.
- Tissue and soil samples were sampled in triplicate, except where noted. Water samples were sampled in duplicate.
- Samples S220801-74, -78, and -79 were small, allowing for only one, two, and two replicates respectively.
- The control matrices used for QC analysis were as follows:

Sample Matrix	Control Matrix
Invertebrates	Crickets (S221018-01)
Fish	Sea bass (S220906-01)
Crustaceans	Crickets (S221013-01)
Reptiles	Rat liver (S221018-02)
Food producing plants	Limes (S221013-01)
Soil	Soil (S090107-04B)
Bird livers	Bird liver (S221018-03)
Fresh Water	Poudre River water (S200609-09)
Sea Water	Synthetic sea water (NWRC #7262)

Contact the author for further details on QA/QC certification at Carmen.Antaky@usda.gov

Analyst	Date	QC Specialist	Date	Reviewer	Date

Sample Preparation and Extraction:

Homogenization:

Baits were homogenized in 10-in^3 canisters, transferred immediately to vacuum sealable bags, and stored at -20°C.

Tissue and Soil Extraction Procedure:

1. Weighed 0.075-0.125g of sample into 1.5-mL microcentrifuge tubes.
2. Added 0.100 mL ultrapure water and vortex mixed to form a slurry.
3. Added 0.020 mL of 75X d_4-Brodifacoum Surrogate Stock to each sample.
4. Added 1.180 mL acetonitrile to each sample and vortex mixed for 10-15 seconds.
5. Added 150 mg sodium chloride to each sample and vortex mixed twice for 2-3 seconds.
6. Centrifuged @14,000 x g for 1 minute.
7. Transferred the 0.900 mL of the acetonitrile layer to a clean 1.5-mL dSPE tube.
8. Vortexed samples for 1-2 seconds.
9. Centrifuged @14,000 x g for 1 minute.
10. Transferred the 0.400 mL of the supernatant to a clean 1.5-mL microfuge tube.
11. Evaporated off solvent under a gentle stream of nitrogen gas in a 50°C water bath.
12. Added 0.100 mL acetonitrile.
13. Vortexed for 8-10 seconds.
14. Added 0.400 mL pH 9.5, 20-mM ammonium acetate buffer.
15. Vortexed for 8-10 seconds.
16. Centrifuged @14,000 x g for 1 minute.
17. Transferred to HPLC vials for analysis.

Water Extraction Procedure:

1. Add 60 mL of seawater to a 125-mL separatory funnel.
2. Add 0.020 mL surrogate to all and 0.040 mL of acetonitrile or 75X brodifacoum stock as indicated.
3. Add 20 mL chloroform.
4. Add ~8.5g sodium chloride.
5. Add 10 mL 1M hydrochloric acid.
6. Cap and shake for 8-10s, let set 1 minute. Repeat 2X.
7. Dispense chloroform phase into 25-mL glass tube taking care to not transfer any water phase.
8. Remove solvent in a 60°C N-Evap with a gentle flow of nitrogen gas.
9. Add 0.300 mL acetonitrile, vortex thoroughly, wetting as much of the inside surface of the tube as possible.
10. Add 1.200 mL pH 9.5 20-mM ammonium acetate buffer and vortex thoroughly.
11. Transfer the sample to an autosampler vial and assay by LC-MS/MS.

Instrument Method:

Agilent 1290 Infinity II HPLC with G6470A QQQ

Column	Waters Xbridge BEH C18, 2.5-µm, 2.1 x 50 mm,
Mobile phase A	90%(pH 9.5 20-mM ammonium acetate)/10%(Acetonitrile)
Mobile phase B	Acetonitrile
Flow rate	0.900 mL/min
Column temp.	60°C
Injection volume	7.5 µL
Run time	5.25 min
Source	AJS ESI, negative mode
Gas temp.	225°C
Gas flow	6 L/min
Nebulizer	40 psi
Sheath gas	375°C, 12 L/min
Capillary	-3500 V
Nozzle	0 V

Time (min)	%A	%B
0.00	100%	0%
0.20	100%	0%
4.50	40%	60%
4.51	0%	100%
4.90	0%	100%
4.91	100%	0%

Analyte	Precursor Ion (m/z)	Product Ion (m/z)	Fragmentor (V)	Collision Energy (V)
Brodifacoum	521.1	**78.9**	213	52
		135		44
d_4-Brodifacoum	525.1	78.9	212	50

Bold=Transition used for quantitation

Method Limit of Detection/Quantitation (MLOD/MLOQ) Values:

Method detection and quantitation limits were determined from kestrel liver during method development. No matrix specific detection or quantitation limits were determined for the different matrices in this invoice. Evaluation of the chromatography indicates that the method detection and quantitation limits are representative for all tissue and soil matrices tested in this invoice.

Method detection and quantitation limits for water samples were determined during sample analysis.

Method Limit of Detection (MLOD)

Matrix	Detection Limit
Tissues and Soils	1.9 ng/g
Fresh Water	0.095 ng/mL
Sea Water	0.073 ng/mL

Method Limit of Quantitation (MLOQ)

Matrix	Quantitation Limit
Tissues and Soils	6.37 ng/g
Fresh Water	0.316 ng/mL
Sea Water	0.271 ng/mL

Results:

Invertebrates:

Sample ID	Sample Description	Analysis Date	Brodifacoum (ng/g)	Descriptive Statistics	
S220801-01-A	Invertebrates; A-I-Baseline-Grubs; A -	12/27/22	ND	$Mean_3$=	ND
S220801-01-B	Radar; Grubs ; Emerald beetle – Protaetia	12/27/22	ND	sd=	N/A
S220801-01-C	pryeri (2); 7/6/22 CA	12/27/22	ND	cv=	N/A
S220801-02-A	Invertebrates; A-II-Baseline-Grubs; A -	12/27/22	ND	$Mean_3$=	ND
S220801-02-B	Radar; Grubs; Emerald beetle – Protaetia	12/27/22	ND	sd=	N/A
S220801-02-C	pryeri (2); 7/6/22 CA	12/27/22	ND	cv=	N/A
S220801-03-A	Invertebrates; B-I-Baseline-Grubs; B -	12/27/22	ND	$Mean_3$=	ND
S220801-03-B	Brackish; Grubs; Emerald beetle – Protaetia	12/27/22	ND	sd=	N/A
S220801-03-C	pryeri (2); 7/6/22 CA	12/27/22	ND	cv=	N/A
S220801-04-A	Invertebrates; B-II-Baseline-Grubs; B -	12/27/22	ND	$Mean_3$=	ND
S220801-04-B	Brackish; Grubs; Emerald beetle – Protaetia	12/27/22	ND	sd=	N/A
S220801-04-C	pryeri (2); 7/6/22 CA	12/27/22	ND	cv=	N/A
S220801-05-A	Invertebrates; C-I-Baseline-Grubs; C – Rusty	12/27/22	ND	$Mean_3$=	ND
S220801-05-B	Bucket; Grubs; Emerald beetle – Protaetia	12/27/22	ND	sd=	N/A
S220801-05-C	pryeri (2); 7/6/22 CA	12/27/22	ND	cv=	N/A
S220801-06-A	Invertebrates; C-II-Baseline-Grubs; C –	12/27/22	ND	$Mean_3$=	ND
S220801-06-B	Rusty Bucket; Grubs; Emerald beetle –	12/27/22	ND	sd=	N/A
S220801-06-C	Protaetia pryeri (2); 7/6/22 CA	12/27/22	ND	cv=	N/A
S220801-07-A	Invertebrates; A-I-Baseline-Roaches; A -	12/27/22	ND	$Mean_3$=	ND
S220801-07-B	Radar; Roaches; Cockroaches - Blattodea sp.	12/27/22	ND	sd=	N/A
S220801-07-C	(7); 7/7/22 CA	12/27/22	ND	cv=	N/A
S220801-08-A	Invertebrates; A-II-Baseline-Roaches; A -	12/27/22	ND	$Mean_3$=	ND
S220801-08-B	Radar; Roaches; Cockroaches - Blattodea sp.	12/27/22	ND	sd=	N/A
S220801-08-C	(4); 7/7/22 CA	12/27/22	ND	cv=	N/A
S220801-09-A	Invertebrates; B-I-Baseline-Roaches; B -	12/27/22	ND	$Mean_3$=	ND
S220801-09-B	Brackish; Roaches; Cockroaches - Blattodea	12/27/22	ND	sd=	N/A
S220801-09-C	sp. (4); 7/7/22 CA	12/27/22	ND	cv=	N/A
S220801-10-A	Invertebrates; B-II-Baseline-Roaches; B -	12/27/22	ND	$Mean_3$=	ND
S220801-10-B	Brackish; Roaches; Cockroaches - Blattodea	12/27/22	ND	sd=	N/A
S220801-10-C	sp. (3); 7/7/22 CA	12/27/22	ND	cv=	N/A
S220801-11-A	Invertebrates; C-I-Baseline-Roaches; C –	12/27/22	ND	$Mean_3$=	ND
S220801-11-B	Rusty Bucket; Roaches; Cockroaches -	12/27/22	ND	sd=	N/A
S220801-11-C	Blattodea sp. (7); 7/7/22 CA	12/27/22	ND	cv=	N/A
S220801-12-A	Invertebrates; C-II-Baseline-Roaches; C –	12/27/22	ND	$Mean_3$=	ND
S220801-12-B	Rusty Bucket; Roaches; Cockroaches -	12/27/22	ND	sd=	N/A
S220801-12-C	Blattodea sp. (7); 7/7/22 CA	12/27/22	ND	cv=	N/A

ND = Not Detected.

Invertebrates:

Sample ID	Sample Description	Analysis Date	Brodifacoum (ng/g)	Descriptive Statistics	
S220801-13-A	Invertebrates; A-I-Baseline-Pitfall; A -	12/28/22	ND	$Mean_3$=	ND
S220801-13-B	Radar; Pitfall; Pooled - Arthropoda spp.;	12/28/22	ND	sd=	N/A
S220801-13-C	7/6/22 CA	12/28/22	ND	cv=	N/A
S220801-14-A	Invertebrates; A-II-Baseline-Pitfall; A -	12/28/22	ND	$Mean_3$=	ND
S220801-14-B	Radar; Pitfall; Pooled - Arthropoda spp.;	12/28/22	ND	sd=	N/A
S220801-14-C	7/7/22 CA	12/28/22	ND	cv=	N/A
S220801-15-A	Invertebrates; B-I-Baseline-Pitfall; B -	12/28/22	ND	$Mean_3$=	ND
S220801-15-B	Brackish; Pitfall; Pooled - Arthropoda spp.;	12/28/22	ND	sd=	N/A
S220801-15-C	7/6/22 CA	12/28/22	ND	cv=	N/A
S220801-16-A	Invertebrates; B-II-Baseline-Pitfall; B -	12/28/22	ND	$Mean_3$=	ND
S220801-16-B	Brackish; Pitfall; Pooled - Arthropoda spp.;	12/28/22	ND	sd=	N/A
S220801-16-C	7/7/22 CA	12/28/22	ND	cv=	N/A
S220801-17-A	Invertebrates; C-I-Baseline-Pitfall; C – Rusty	12/28/22	ND	$Mean_3$=	ND
S220801-17-B	Bucket; Pitfall; Pooled - Arthropoda spp.;	12/28/22	ND	sd=	N/A
S220801-17-C	7/6/22 CA	12/28/22	ND	cv=	N/A
S220801-18-A	Invertebrates; C-II-Baseline-Pitfall; C –	12/28/22	ND	$Mean_3$=	ND
S220801-18-B	Rusty Bucket; Pitfall; Pooled - Arthropoda	12/28/22	ND	sd=	N/A
S220801-18-C	spp.; 7/7/22 CA	12/28/22	ND	cv=	N/A

ND = Not Detected.

Fish:

Sample ID	Sample Description	Analysis Date	Brodifacoum (ng/g)	Descriptive Statistics	
S220801-19-A	Fish; A-I-Baseline-Mosquitofish; A – R2;	12/02/22	ND	$Mean_3$=	ND
S220801-19-B	Mosquito-fish; Mosquito Fish – Gambusia	12/02/22	ND	sd=	N/A
S220801-19-C	affinus (10); 7/1/22 CA	12/02/22	ND	cv=	N/A
S220801-20-A	Fish; A-II-Baseline-Mosquitofish; A – R2;	12/02/22	ND	$Mean_3$=	ND
S220801-20-B	Mosquito-fish; Mosquito Fish – Gambusia	12/02/22	ND	sd=	N/A
S220801-20-C	affinus (10); 7/1/22 CA	12/02/22	ND	cv=	N/A
S220801-21-A	Fish; A-I-Baseline-Bait Fish; A – Cargo Pier;	12/02/22	ND	$Mean_3$=	ND
S220801-21-B	Bait Fish; Flagtail – Kuhlia sandvicensis (1);	12/02/22	ND	sd=	N/A
S220801-21-C	6/30/22 CA	12/02/22	ND	cv=	N/A
S220801-22-A	Fish; A-II-Baseline-Bait Fish; A – Cargo	12/02/22	ND	$Mean_3$=	ND
S220801-22-B	Pier; Bait Fish; Flagtail – Kuhlia	12/02/22	ND	sd=	N/A
S220801-22-C	sandvicensis (1); 6/30/22 CA	12/02/22	ND	cv=	N/A
S220801-23-A	Fish; B-I-Baseline-Bait Fish; B – Hale Honu;	12/02/22	ND	$Mean_3$=	ND
S220801-23-B	Bait Fish; Mullet – Mugil sp. (3); 6/30/22	12/02/22	ND	sd=	N/A
S220801-23-C	CA	12/02/22	ND	cv=	N/A
S220801-24-A	Fish; B-II-Baseline-Bait Fish; B – Hale	12/02/22	ND	$Mean_3$=	ND
S220801-24-B	Honu; Bait Fish; Flagtail – Kuhlia	12/02/22	ND	sd=	N/A
S220801-24-C	sandvicensis (10); 6/30/22 CA	12/02/22	ND	cv=	N/A
S220801-25-A	Fish; C-I-Baseline-Bait Fish; C – Rusty	12/02/22	ND	$Mean_3$=	ND
S220801-25-B	Bucket; Bait Fish; Flagtail – Kuhlia	12/02/22	ND	sd=	N/A
S220801-25-C	sandvicensis (2); 7/1/22 CA	12/02/22	ND	cv=	N/A
S220801-26-A	Fish; C-II-Baseline-Bait Fish; C – Rusty	12/02/22	ND	$Mean_3$=	ND
S220801-26-B	Bucket; Bait Fish; Flagtail – Kuhlia	12/02/22	ND	sd=	N/A
S220801-26-C	sandvicensis (2); 7/1/22 CA	12/02/22	ND	cv=	N/A
S220801-27-A	Fish; A-I-Baseline-Reef Fish; A – Cargo	12/02/22	ND	$Mean_3$=	ND
S220801-27-B	Pier; Reef Fish; Moi – Polydactylys sexfilis	12/02/22	ND	sd=	N/A
S220801-27-C	(1); 6/30/22 CA	12/02/22	ND	cv=	N/A
S220801-28-A	Fish; A-II-Baseline-Reef Fish; A – Cargo	12/02/22	ND	$Mean_3$=	ND
S220801-28-B	Pier; Reef Fish; Moi – Polydactylys sexfilis	12/02/22	ND	sd=	N/A
S220801-28-C	(1); 6/30/22 CA	12/02/22	ND	cv=	N/A
S220801-29-A	Fish; A-I-Baseline-Reef Fish2; A – Cargo	12/02/22	ND	$Mean_3$=	ND
S220801-29-B	Pier; Reef Fish; Trevally – Caranx sp. (1);	12/02/22	ND	sd=	N/A
S220801-29-C	6/30/22 CA	12/02/22	ND	cv=	N/A
S220801-30-A	Fish; A-II-Baseline-Reef Fish2; A – Cargo	12/02/22	ND	$Mean_3$=	ND
S220801-30-B	Pier; Reef Fish; Trevally – Caranx sp. (1);	12/02/22	ND	sd=	N/A
S220801-30-C	6/30/22 CA	12/02/22	ND	cv=	N/A

ND = Not Detected.

Fish:

Sample ID	Sample Description	Analysis Date	Brodifacoum (ng/g)	Descriptive Statistics	
S220801-31-A	Fish; A-I-Baseline-Reef Fish3; A – Cargo	12/02/22	ND	$Mean_3$=	ND
S220801-31-B	Pier; Reef Fish; Hogfish – Bodianus	12/02/22	ND	sd=	N/A
S220801-31-C	bilunulatus (1); 7/8/22 CA	12/02/22	ND	cv=	N/A
S220801-32-A	Fish; B-I-Baseline-Reef; B – Hale Honu;	12/02/22	ND	$Mean_3$=	ND
S220801-32-B	Reef Fish; Moi – Polydactylys sexfilis (1);	12/02/22	ND	sd=	N/A
S220801-32-C	7/1/22 CA	12/02/22	ND	cv=	N/A
S220801-33-A	Fish; B-II-Baseline-Reef; B – Hale Honu;	12/02/22	ND	$Mean_3$=	ND
S220801-33-B	Reef Fish; Moi – Polydactylys sexfilis (1);	12/02/22	ND	sd=	N/A
S220801-33-C	7/1/22 CA	12/02/22	ND	cv=	N/A
S220801-34-A	Fish; B-I-Baseline-Reef2; B – Hale Honu;	12/02/22	ND	$Mean_3$=	ND
S220801-34-B	Reef Fish; Goatfish -Mulloidichtys	12/02/22	ND	sd=	N/A
S220801-34-C	flavolineatus (1); 7/1/22 CA	12/02/22	ND	cv=	N/A
S220801-35-A	Fish; B-II-Baseline-Reef2; B – Hale Honu;	12/02/22	ND	$Mean_3$=	ND
S220801-35-B	Reef Fish; Goatfish -Mulloidichtys	12/02/22	ND	sd=	N/A
S220801-35-C	flavolineatus (1); 7/1/22 CA	12/02/22	ND	cv=	N/A
S220801-36-A	Fish; C-I-Baseline-Reef; C – Rusty Bucket;	12/02/22	ND	$Mean_3$=	ND
S220801-36-B	Reef Fish; Moi – Polydactylys sexfilis (1);	12/02/22	ND	sd=	N/A
S220801-36-C	7/1/22 CA	12/02/22	ND	cv=	N/A
S220801-37-A	Fish; C-II-Baseline-Reef; C – Rusty Bucket;	12/02/22	ND	$Mean_3$=	ND
S220801-37-B	Reef Fish; Moi – Polydactylys sexfilis (1);	12/02/22	ND	sd=	N/A
S220801-37-C	7/1/22 CA	12/02/22	ND	cv=	N/A
S220801-38-A	Fish; C-I-Baseline-Reef2; C – Rusty Bucket;	12/02/22	ND	$Mean_3$=	ND
S220801-38-B	Reef Fish; Goatfish -Mulloidichtys	12/02/22	ND	sd=	N/A
S220801-38-C	flavolineatus (1); 7/1/22 CA	12/02/22	ND	cv=	N/A
S220801-39-A	Fish; C-II-Baseline-Reef2; C – Rusty	12/02/22	ND	$Mean_3$=	ND
S220801-39-B	Bucket; Reef Fish; Goatfish -Mulloidichtys	12/02/22	ND	sd=	N/A
S220801-39-C	flavolineatus (1); 7/1/22 CA	12/02/22	ND	cv=	N/A
S220801-40-A	Fish; C-I-Baseline-Reef3; C – Rusty Bucket;	12/02/22	ND	$Mean_3$=	ND
S220801-40-B	Reef Fish; Taape – Lutjans kasmira (1);	12/02/22	ND	sd=	N/A
S220801-40-C	7/8/22 CA	12/02/22	ND	cv=	N/A

ND = Not Detected.

Crustaceans:

Sample ID	Sample Description	Analysis Date	Brodifacoum (ng/g)	Descriptive Statistics	
S220801-41-A	Crustaceans; A-I-Baseline-Ghostcrabs; A –	12/28/22	ND	$Mean_3$=	ND
S220801-41-B	Cargo Pier; Ghostcrabs; Ghostcrab - Ocypode	12/28/22	ND	sd=	N/A
S220801-41-C	pallidula (4); 6/30/22 CA	12/28/22	ND	cv=	N/A
S220801-42-A	Crustaceans; A-II-Baseline-Ghostcrabs; A –	12/28/22	ND	$Mean_3$=	ND
S220801-42-B	Cargo Pier; Ghostcrabs; Ghostcrab - Ocypode	12/28/22	ND	sd=	N/A
S220801-42-C	pallidula (4); 6/30/22 CA	12/28/22	ND	cv=	N/A
S220801-43-A	Crustaceans; B-I-Baseline-Ghostcrabs; B –	12/28/22	ND	$Mean_3$=	ND
S220801-43-B	Hale Honu; Ghostcrabs; Ghostcrab - Ocypode	12/28/22	ND	sd=	N/A
S220801-43-C	pallidula (4); 6/30/22 CA	12/28/22	ND	cv=	N/A
S220801-44-A	Crustaceans; B-II-Baseline- Ghostcrabs; B –	12/28/22	ND	$Mean_3$=	ND
S220801-44-B	Hale Honu; Ghostcrabs; Ghostcrab - Ocypode	12/28/22	ND	sd=	N/A
S220801-44-C	pallidula (4); 6/30/22 CA	12/28/22	ND	cv=	N/A
S220801-45-A	Crustaceans; C-I-Baseline-Ghostcrabs; C –	12/28/22	ND	$Mean_3$=	ND
S220801-45-B	Rusty Bucket; Ghostcrabs; Ghostcrab -	12/28/22	ND	sd=	N/A
S220801-45-C	Ocypode pallidula (4); 7/1/22 CA	12/28/22	ND	cv=	N/A
S220801-46-A	Crustaceans; C-II-Baseline-Ghostcrabs; C –	12/28/22	ND	$Mean_3$=	ND
S220801-46-A	Rusty Bucket; Ghostcrabs; Ghostcrab -	12/28/22	ND	sd=	N/A
S220801-46-A	Ocypode pallidula (4); 7/1/22 CA	12/28/22	ND	cv=	N/A

ND = Not Detected.

Reptiles:

Sample ID	Sample Description	Analysis Date	Brodifacoum (ng/g)	Descriptive Statistics	
S220801-47-A	Reptiles; A-I-Baseline-Geckos; A – Clipper	12/20/22	ND	$Mean_3$=	ND
S220801-47-B	House; Geckos; Common House Gecko –	12/20/22	ND	sd=	N/A
S220801-47-C	Hemidactylus frenatus (2); 6/30/22 CA	12/20/22	ND	cv=	N/A
S220801-48-A	Reptiles; A-II-Baseline-Geckos; A – Clipper	12/20/22	ND	$Mean_3$=	ND
S220801-48-B	House; Geckos; Mourning Gecko –	12/20/22	ND	sd=	N/A
S220801-48-C	Lepidodactylus lugubris (2); 6/30/22 CA	12/20/22	ND	cv=	N/A
S220801-49-A	Reptiles; B-I-Baseline-Geckos; B –	12/20/22	ND	$Mean_3$=	ND
S220801-49-B	Gym/Store; Geckos; Common House Gecko –	12/20/22	ND	sd=	N/A
S220801-49-C	Hemidactylus frenatus (2); 7/5/22 CA	12/20/22	ND	cv=	N/A
S220801-50-A	Reptiles; B-II-Baseline-Geckos; B –	12/20/22	ND	$Mean_3$=	ND
S220801-50-B	Gym/Store; Geckos; Common House Gecko –	12/20/22	ND	sd=	N/A
S220801-50-C	Hemidactylus frenatus (2); 7/5/22 CA	12/20/22	ND	cv=	N/A
S220801-51-A	Reptiles; C-I-Baseline- Geckos; C – FWS	12/20/22	ND	$Mean_3$=	ND
S220801-51-B	Office; Geckos; Common House Gecko –	12/20/22	ND	sd=	N/A
S220801-51-C	Hemidactylus frenatus (2); 7/5/22 CA	12/20/22	ND	cv=	N/A
S220801-52-A	Reptiles; C-II-Baseline- Geckos; C – FWS	12/20/22	ND	$Mean_3$=	ND
S220801-52-B	Office; Geckos; Common House Gecko –	12/20/22	ND	sd=	N/A
S220801-52-C	Hemidactylus frenatus (2); 7/5/22 CA	12/20/22	ND	cv=	N/A

ND = Not Detected.

Food Producing Plants:

Sample ID	Sample Description	Analysis Date	Brodifacoum (ng/g)	Descriptive Statistics	
S220801-53-A	Food-producing plants; A-I-Baseline-Plants;	12/16/22	ND	$Mean_3$=	ND
S220801-53-B	A – Community Garden; Fruit; Papaya –	12/16/22	ND	sd=	N/A
S220801-53-C	Carica papaya (1); 7/4/22 CA	12/26/22	ND	cv=	N/A
S220801-54-A	Food-producing plants; A-II-Baseline-Plants;	12/16/22	ND	$Mean_3$=	ND
S220801-54-B	A – Community Garden; Fruit; Papaya –	12/16/22	ND	sd=	N/A
S220801-54-C	Carica papaya (1); 7/4/22 CA	12/26/22	ND	cv=	N/A
S220801-55-A	Food-producing plants; B-I-Baseline-Plants; B	12/16/22	ND	$Mean_3$=	ND
S220801-55-B	– Community Garden; Fruit; Lime – Citrus sp.	12/16/22	ND	sd=	N/A
S220801-55-C	(1); 7/4/22 CA	12/26/22	ND	cv=	N/A
S220801-56-A	Food-producing plants; B-II-Baseline-Plants;	12/16/22	ND	$Mean_3$=	ND
S220801-56-B	B – Community Garden; Fruit; Lime – Citrus	12/16/22	ND	sd=	N/A
S220801-56-C	sp. (1); 7/4/22 CA	12/26/22	ND	cv=	N/A
S220801-57-A	Food-producing plants; C-I-Baseline-Plants; C	12/16/22	ND	$Mean_3$=	ND
S220801-57-B	– Orchard; Fruit; Orange – Citrus sp. (1);	12/16/22	ND	sd=	N/A
S220801-57-C	7/4/22 CA	12/26/22	ND	cv=	N/A
S220801-58-A	Food-producing plants; C-II-Baseline-Plants;	12/16/22	ND	$Mean_3$=	ND
S220801-58-B	C - Orchard; Fruit; Orange – Citrus sp. (1);	12/16/22	ND	sd=	N/A
S220801-58-C	7/4/22 CA	12/26/22	ND	cv=	N/A

ND = Not Detected.

Soils:

Sample ID	Sample Description	Analysis Date	Brodifacoum (ng/g)	Descriptive Statistics	
S220801-59-A	Soil; A-I-Baseline-Soil; A -Radar; Soil; ;	12/20/22	ND	$Mean_3$=	ND
S220801-59-B	7/5/22 CA	12/20/22	ND	sd=	N/A
S220801-59-C		12/20/22	ND	cv=	N/A
S220801-60-A	Soil; B-I-Baseline-Soil; B – Brackish; Soil; ;	12/20/22	ND	$Mean_3$=	ND
S220801-60-B	7/5/22 CA	12/20/22	ND	sd=	N/A
S220801-60-C		12/20/22	ND	cv=	N/A
S220801-61-A	Soil; C-I-Baseline-Soil; C – Community	12/20/22	ND	$Mean_3$=	ND
S220801-61-B	Garden; Soil; ; 7/4/22 CA	12/20/22	ND	sd=	N/A
S220801-61-C		12/20/22	ND	cv=	N/A

ND = Not Detected.

Waters:

Sample ID	Sample Description	Analysis Date	Brodifacoum (ng/g)	Descriptive Statistics	
S220801-62-A	Drinking Water; A-I-Baseline-	12/30/22	ND	$Mean_2$=	ND
S220801-62-B	DrinkingWater; A – FWS Office; Water; ;	12/30/22	ND	sd=	N/A
	7/7/22 CA			cv=	N/A
S220801-63-A	Drinking Water; B-I-Baseline-	12/30/22	ND	$Mean_2$=	ND
S220801-63-B	DrinkingWater; B – Charlie Barracks; Water;	12/30/22	ND	sd=	N/A
	; 7/7/22 CA			cv=	N/A
S220801-64-A	Water Collection; A-I-Baseline-	12/30/22	ND	$Mean_2$=	ND
S220801-64-B	WaterCollection; A – Catchment drainage	12/30/22	ND	sd=	N/A
	access; Water; ; 7/7/22 CA			cv=	N/A
S220801-65-A	Water Collection; B-I-Baseline-	12/30/22	ND	$Mean_2$=	ND
S220801-65-B	WaterCollection; B – Bowling Alley; Water;	12/30/22	ND	sd=	N/A
	; 7/7/22 CA			cv=	N/A
S220801-66-A	Ocean Water; A-I-Baseline-OceanWater; A –	12/30/22	ND	$Mean_2$=	ND
S220801-66-B	Cargo Pier; Water; ; 6/30/22 CA	12/30/22	ND	sd=	N/A
				cv=	N/A
S220801-67-A	Ocean Water; B-I-Baseline-OceanWater; B –	12/30/22	ND	$Mean_2$=	ND
S220801-67-B	Hale Honu; Water; ; 6/30/22 CA	12/30/22	ND	sd=	N/A
				cv=	N/A
S220801-68-A	Ocean Water; C-I-Baseline-OceanWater; C –	12/30/22	ND	$Mean_2$=	ND
S220801-68-B	Rusty Bucket; Water; ; 7/1/22 CA	12/30/22	ND	sd=	N/A
				cv=	N/A
S220801-69-A	Ocean Water; D-I-Baseline-OceanWater; D –	12/30/22	ND	$Mean_2$=	ND
S220801-69-B	Harbor; Water; ; 7/1/22 CA	12/30/22	ND	sd=	N/A
				cv=	N/A
S220801-70-A	Fresh Water; A-I-Baseline-FreshWater; A –	12/30/22	ND	$Mean_2$=	ND
S220801-70-B	Radar; Water; ; 7/5/22 CA	12/30/22	ND	sd=	N/A
				cv=	N/A
S220801-71-A	Fresh Water; B-I-Baseline-FreshWater; B –	12/30/22	ND	$Mean_2$=	ND
S220801-71-B	Brackish; Water; ; 7/5/22 CA	12/30/22	ND	sd=	N/A
				cv=	N/A
S220801-72-A	Fresh Water; C-I-Baseline- FreshWater; C –	12/30/22	ND	$Mean_2$=	ND
S220801-72-B	R2; Water; ; 7/5/22 CA	12/30/22	ND	sd=	N/A
				cv=	N/A
S220801-73-A	Fresh Water; D-I-Baseline- FreshWater; D –	12/30/22	ND	$Mean_2$=	ND
S220801-73-B	Office Guzzler; Water; ; 7/5/22 CA	12/30/22	ND	sd=	N/A
				cv=	N/A

ND = Not Detected.

Bird Livers:

Sample ID	Sample Description	Analysis Date	Brodifacoum (ng/g)	Descriptive Statistics	
S220801-74-A	Duck Carcasses; A-I-Baseline-Duck;	12/16/22	ND	$Mean_2$=	ND
S220801-74-B	Catchment; Duck; Laysan Duck - Anas	12/16/22	ND	sd=	N/A
S220801-74-C	laysanensis (1); 6/9/22 FWS	Insufficient Sample		cv=	N/A
S220801-75-A	Duck Carcasses; A-II-Baseline-Duck;				
S220801-75-B	Catchment; Duck; Laysan Duckling - Anas	No Sample			
S220801-75-C	laysanensis (1); 7/5/22 FWS				
S220801-76-A	Shorebird Carcass; A-I-Baseline-Shorebird;	12/16/22	ND	$Mean_3$=	ND
S220801-76-B	Catchment; Shorebird; Pacific Golden Plover	12/16/22	ND	sd=	N/A
S220801-76-C	– Pluvialis fulva (1); 1/20/22 FWS	12/16/22	ND	cv=	N/A
S220801-77-A	Myna Carcass; A-I-Baseline-Myna;	12/16/22	4.8*	$Mean_3$=	4.7*
S220801-77-B	Brackish; Passerines; Myna – Acridotheres	12/16/22	4.5*	sd=	0.17
S220801-77-C	tristis (1); 2/18/21 FWS	12/16/22	4.8*	cv=	3.7%
S220801-78-A	Canary Carcass; A-I-Baseline-Canary; NAF	12/16/22	ND	$Mean_1$=	ND
S220801-78-B	Hanger; Passerines; Canary – Serinus canaria	Insufficient Sample		sd=	N/A
S220801-78-C	(1); 6/29/22 CA	Insufficient Sample		cv=	N/A
S220801-79-A	Seabird Carcass; A-I-Baseline-Seabird;	12/16/22	ND	$Mean_2$=	ND
S220801-79-B	Cargo Pier; Seabird; Bonin Petrel –	12/16/22	ND	sd=	N/A
S220801-79-C	Pterodroma hypoleauca (1); 6/30/22 CA	Insufficient Sample		cv=	N/A
S220801-80-A	Seabird Carcass; A-II-Baseline-Seabird;				
S220801-80-B	South Beach/Iwa Point; Seabird; Laysan	No Sample			
S220801-80-C	Albatross – Phoebastria immutabilis (Liver Tissue Sample); 7/11/22 CA				

ND = Not Detected.

*-Value is below the method quantitation limit of 6.37 ng/g. Result should be considered qualitative only.

QC Results:

Invertebrates/Crustaceans:

ID	Analysis Date	Theoretical Brodifacoum Concentration (ng/g)	Observed Brodifacoum Concentration (ng/g)	% Recovery
QC-41	12/27/22	Control	ND	N/A
QC-42	12/27/22	Control	ND	N/A
QC-49	12/28/22	Control	ND	N/A
QC-50	12/28/22	Control	ND	N/A
QC-43	12/27/22	54.1	54.5	101
QC-44	12/27/22	54.0	53.8	99.6
QC-51	12/28/22	57.4	56.8	99.0
QC-52	12/28/22	49.3	48.6	98.6
QC-45	12/27/22	555	532	95.9
QC-46	12/27/22	571	573	100
QC-53	12/28/22	507	512	101
QC-54	12/28/22	646	646	100
QC-47	12/27/22	2240	2220	99.1
QC-48	12/27/22	2230	2200	98.7
QC-55	12/28/22	2170	2160	99.5
QC-56	12/28/22	2030	2020	99.5

ND = Not Detected.

Fish:

ID	Analysis Date	Theoretical Brodifacoum Concentration (ng/g)	Observed Brodifacoum Concentration (ng/g)	% Recovery
QC-01	12/02/22	Control	ND	N/A
QC-02	12/02/22	Control	ND	N/A
QC-03	12/02/22	50.9	51.8	102
QC-04	12/02/22	55.8	57.7	103
QC-05	12/02/22	544	541	99.4
QC-06	12/02/22	548	549	100
QC-07	12/02/22	1850	1820	98.4
QC-08	12/02/22	1880	1830	97.3

ND = Not Detected.

Reptiles:

ID	Analysis Date	Theoretical Brodifacoum Concentration (ng/g)	Observed Brodifacoum Concentration (ng/g)	% Recovery
QC-25	12/20/22	Control	ND	N/A
QC-26	12/20/22	Control	ND	N/A
QC-27	12/20/22	51.7	50.8	98.3
QC-28	12/20/22	46.3	46.2	99.8
QC-29	12/20/22	541	525	97.0
QC-30	12/20/22	579	565	97.6
QC-31	12/20/22	2000	2060	103
QC-32	12/20/22	2170	2150	99.1

ND = Not Detected.

Food Producing Plants:

ID	Analysis Date	Theoretical Brodifacoum Concentration (ng/g)	Observed Brodifacoum Concentration (ng/g)	% Recovery
QC-09	12/16/22	Control	ND	N/A
QC-10	12/16/22	Control	ND	N/A
QC-11	12/16/22	55.7	62.3	112
QC-12	12/16/22	47.2	46.2	97.9
QC-13	12/16/22	551	541	98.2
QC-14	12/16/22	648	638	98.5
QC-15	12/16/22	2230	2200	98.7
QC-16	12/16/22	2160	2100	97.2

ND = Not Detected.

Soils:

ID	Analysis Date	Theoretical Brodifacoum Concentration (ng/g)	Observed Brodifacoum Concentration (ng/g)	% Recovery
QC-33	12/20/22	Control	ND	N/A
QC-34	12/20/22	Control	ND	N/A
QC-35	12/20/22	56.5	55.2	97.7
QC-36	12/20/22	56.3	54.9	97.5
QC-37	12/20/22	545	532	97.6
QC-38	12/20/22	620	604	97.4
QC-39	12/20/22	2230	2200	98.7
QC-40	12/20/22	2290	2260	98.7

ND = Not Detected.

Fresh Water:

ID	Analysis Date	Theoretical Brodifacoum Concentration (ng/mL)	Observed Brodifacoum Concentration (ng/mL)	% Recovery
QC-57	12/30/22	Control	ND	N/A
QC-58	12/30/22	Control	ND	N/A
QC-59	12/30/22	0.192	0.187*	97.4
QC-60	12/30/22	0.192	0.184*	95.8
QC-61	12/30/22	1.73	1.68	97.1
QC-62	12/30/22	1.73	1.70	98.3
QC-63	12/30/22	5.20	5.26	101
QC-64	12/30/22	5.20	5.28	102

ND = Not Detected.

*-Value is below the method quantitation limit of 0.316 ng/mL.

Sea Water:

ID	Analysis Date	Theoretical Brodifacoum Concentration (ng/mL)	Observed Brodifacoum Concentration (ng/mL)	% Recovery
QC-65	12/30/22	Control	ND	N/A
QC-66	12/30/22	Control	ND	N/A
QC-67	12/30/22	0.192	0.174*	90.6
QC-68	12/30/22	0.192	0.175*	91.1
QC-69	12/30/22	1.73	1.71	98.8
QC-70	12/30/22	1.73	1.68	97.1
QC-71	12/30/22	5.20	4.98	95.8
QC-72	12/30/22	5.20	4.99	96.0

ND = Not Detected.

*-Value is below the method quantitation limit of 0.271 ng/mL.

Bird Livers:

ID	Analysis Date	Theoretical Brodifacoum Concentration (ng/g)	Observed Brodifacoum Concentration (ng/g)	% Recovery
QC-17	12/16/22	Control	ND	N/A
QC-18	12/16/22	Control	ND	N/A
QC-19	12/16/22	49.0	49.0	100
QC-20	12/16/22	44.7	44.7	100
QC-21	12/16/22	551	553	100
QC-22	12/16/22	565	560	99.1
QC-23	12/16/22	2310	2290	99.1
QC-24	12/16/22	1930	1890	97.9

ND = Not Detected.
