# Supplementary material for: Anticoagulant residues associated with an attempted rodent eradication from a subtropical coral atoll
Source: PLoS One. 2026 Mar 23;21(3):e0344972. doi: 10.1371/journal.pone.0344972 (PMC13008109; doi:10.1371/journal.pone.0344972)

To: Carmen Antaky
Biologist
NWRC Hawai'i Field Station

Subject: Determination of brodifacoum in invertebrate matrices from Midway Island (QA-3404)

Methods: 188A "Determination of Multiple Rodenticide Residues in Avian Liver by dSPE and LC-MS/MS" -Non-GLP

Analysis Dates: 08/07/23

Notebook References: AC165, pp.186-187, 190-193
QC35, p.68

Analyst: Ben Abbo

Sample Description:

Twenty invertebrate samples were submitted on 08/03/23. See sample descriptions on pp.3-4.

Additional Comments:

- During the homogenization process, three of the roach samples (S230803-09, -10, and -12) showed a green coloration consistent with the color of the bait. A piece of bait was observed to be part of S230803-10 prior to homogenization. This was documented by photographs attached in an appendix to this report.
- Three replicates of each sample were analyzed unless there was insufficient sample to weigh out three. These are marked as INS in the report. The mean brodifacoum concentration and standard deviation are reported for each sample, with the exceptions of samples S230803-15, -16, and -18. These samples only had sufficient sample to run a single sample.
- Sample S230803-02 had two replicates with brodifacoum at detectable levels, while the third replicate did not have detectable levels of brodifacoum. A value of one-half of the detection limit (0.7 ng/g) was assigned to the non-detected replicate and this value was used with the values of the two positive replicates to calculate a mean and standard deviation for the sample.
- Control crickets (S221018-01) were used as the matrix for QC samples.

Method Limit of Detection/Quantitation (MLOD/MLOQ) Values:

Method detection and quantitation limits were determined from by comparing the noise at the analyte retention in five unfortified control cricket samples to the peak height of brodifacoum in five control cricket samples fortified to ~50 ng/g brodifacoum. The detection limit was determined to be 3X the noise and the quantitation limit was determined to be 10X the noise found in the unfortified samples.

Method Limit of Detection (MLOD)

Matrix	Detection Limit
Invertebrates	1.4 ng/g

Method Limit of Quantitation (MLOQ)

Matrix	Quantitation Limit
Invertebrates	4.69 ng/g

Results:

Sample ID	Sample Description	Brodifacoum Conc (ng/g)	Descriptive Statistics	
S230803-01-A	Invertebrates, A-I-Post1-G, A - Radar,	ND		
S230803-01-B	Grubs, Emerald beetle, 7/6/2023	ND		
S230803-01-C		ND		
S230803-02-A	Invertebrates, A-II-Post1-G, A - Radar,	ND	$Mean_3$=	1.5*
S230803-02-B	Grubs, Emerald beetle, 7/6/2023	1.9*	sd=	0.69
S230803-02-C		1.9*	cv=	46%
S230803-03-A	Invertebrates, B-I-Post1-G, B - Brackish,	ND		
S230803-03-B	Grubs, Emerald beetle, 7/6/2023	ND		
S230803-03-C		ND		
S230803-04-A	Invertebrates, B-II-Post1-G, B - Brackish,	ND		
S230803-04-B	Grubs, Emerald beetle, 7/6/2023	ND		
S230803-04-C		ND		
S230803-05-A	Invertebrates, C-I-Post1-G, C – Rusty	ND		
S230803-05-B	Bucket, Grubs, Emerald beetle, 7/6/2023	ND		
S230803-05-C		ND		
S230803-06-A	Invertebrates, C-II-Post1-G, C – Rusty	ND		
S230803-06-B	Bucket, Grubs, Emerald beetle, 7/6/202	ND		
S230803-06-C		ND		
S230803-07-A	Invertebrates, A-I-Post1-R, A - Radar,	ND		
S230803-07-B	Roaches, Blattodea, 7/6/2023	INS		
S230803-07-C		INS		
S230803-08-A	Invertebrates, A-II-Post1-R, A - Radar,	ND		
S230803-08-B	Roaches, Blattodea, 7/6/2023	ND		
S230803-08-C		INS		
S230803-09-A	Invertebrates, B-I-Post1-R, B - Brackish,	2670	$Mean_3$=	2420
S230803-09-B	Roaches, Blattodea, 7/6/2023	2130	sd=	270
S230803-09-C		2470	cv=	11%
S230803-10-A	Invertebrates, B-II-Post1-R, B - Brackish,	3770	$Mean_3$=	3650
S230803-10-B	Roaches, Blattodea, 7/6/2023	3250	sd=	350
S230803-10-C		3920	cv=	9.6%
S230803-11-A	Invertebrates, C-I-Post1-R, C – Rusty	338	Avg_2=	322
S230803-11-B	Bucket, Roaches, Blattodea, 7/6/2023	306	sd=	23
S230803-11-C		INS	cv=	7.1%
S230803-12-A	Invertebrates, C-II-Post1-R, C – Rusty	2010	$Mean_3$=	2090
S230803-12-B	Bucket, Roaches, Blattodea, 7/6/2023	1890	sd=	240
S230803-12-C		2360	cv=	11%

ND = Not Detected.
*-Value is below the method quantitation limit of 4.69 ng/g
INS = Insufficient Sample

Results:

Sample ID	Sample Description	Brodifacoum (ng/g)	Descriptive Statistics	
S230803-13-A	Invertebrates, A-I-Post1-P, A - Radar, Pitfall,	4.2*	Avg_2=	4.2*
S230803-13-B	Pooled, 7/4/2023	4.1*	sd=	0.071
S230803-13-C		INS	cv=	1.7%
S230803-14-A	Invertebrates, A-II-Post1-P, A - Radar,	1.5*	$Mean_3$=	1.6*
S230803-14-B	Pitfall, Pooled, 7/4/2023	1.7*	sd=	0.14
S230803-14-C		INS	cv=	8.8%
S230803-15-A	Invertebrates, B-I-Post1-P, B - Brackish,	83.4	Value=	83.4
S230803-15-B	Pitfall, Pooled, 7/4/2023	INS		
S230803-15-C		INS		
S230803-16-A	Invertebrates, B-II-Post1-P, B - Brackish,	43.6	Value=	43.6
S230803-16-B	Pitfall, Pooled, 7/4/2023	INS		
S230803-16-C		INS		
S230803-17-A	Invertebrates, C-I-Post1-P, C – Rusty	48.1	$Mean_3$=	47.0
S230803-17-B	Bucket, Pitfall, Pooled, 7/4/2023	52.8	sd=	6.4
S230803-17-C		40.2	cv=	14%
S230803-18-A	Invertebrates, C-II-Post1-P, C – Rusty	1540	Value=	1540
S230803-18-B	Bucket, Pitfall, Pooled, 7/4/2023	INS		
S230803-18-C		INS		
S230803-19-A	Invertebrates, A-I-Post1-Con, Cargo Pier,	ND		
S230803-19-B	Marine Invert, Lined fireworm, 7/4/2023	ND		
S230803-19-C		ND		
S230803-20-A	Invertebrates, A-II-Post1-Con, Cargo Pier,	ND		
S230803-20-B	Marine Invert, Lined fireworm, 7/4/2023	ND		
S230803-20-C		ND		

ND = Not Detected.
*-Value is below the method quantitation limit of 4.69 ng/g
INS = Insufficient Sample

QC Results:

ID	Theoretical Brodifacoum Concentration (ng/g)	Observed Brodifacoum Concentration (ng/g)	% Recovery	Descriptive Statistics	
QC-01	Control	ND	N/A		
QC-02	Control	ND	N/A		
QC-03	Control	ND	N/A		
QC-04	Control	ND	N/A		
QC-05	Control	ND	N/A		
QC-06	49.3	46.4	94.1		
QC-07	47.8	46.4	97.1	$Mean_5$=	92.8%
QC-08	53.8	50.3	93.5	sd=	3.7%
QC-09	45.5	39.6	87.0	cv=	4.0%
QC-10	58.8	54.3	92.3		
QC-11	549	531	96.7		
QC-12	580	557	96.0	$Mean_5$=	95.5%
QC-13	638	603	94.5	sd=	0.87%
QC-14	611	581	95.1	cv=	0.91%
QC-15	534	508	95.1		
QC-16	1840	1840	100		
QC-17	2110	1970	93.4	$Mean_5$=	95.3%
QC-18	1990	1900	95.5	sd=	2.7%
QC-19	2060	1930	93.7	cv=	2.8%
QC-20	2020	1900	94.1		

ND = Not Detected.

Appendix:

Figure 1: Roach sample after homogenization showing signs of the presence of bait material.

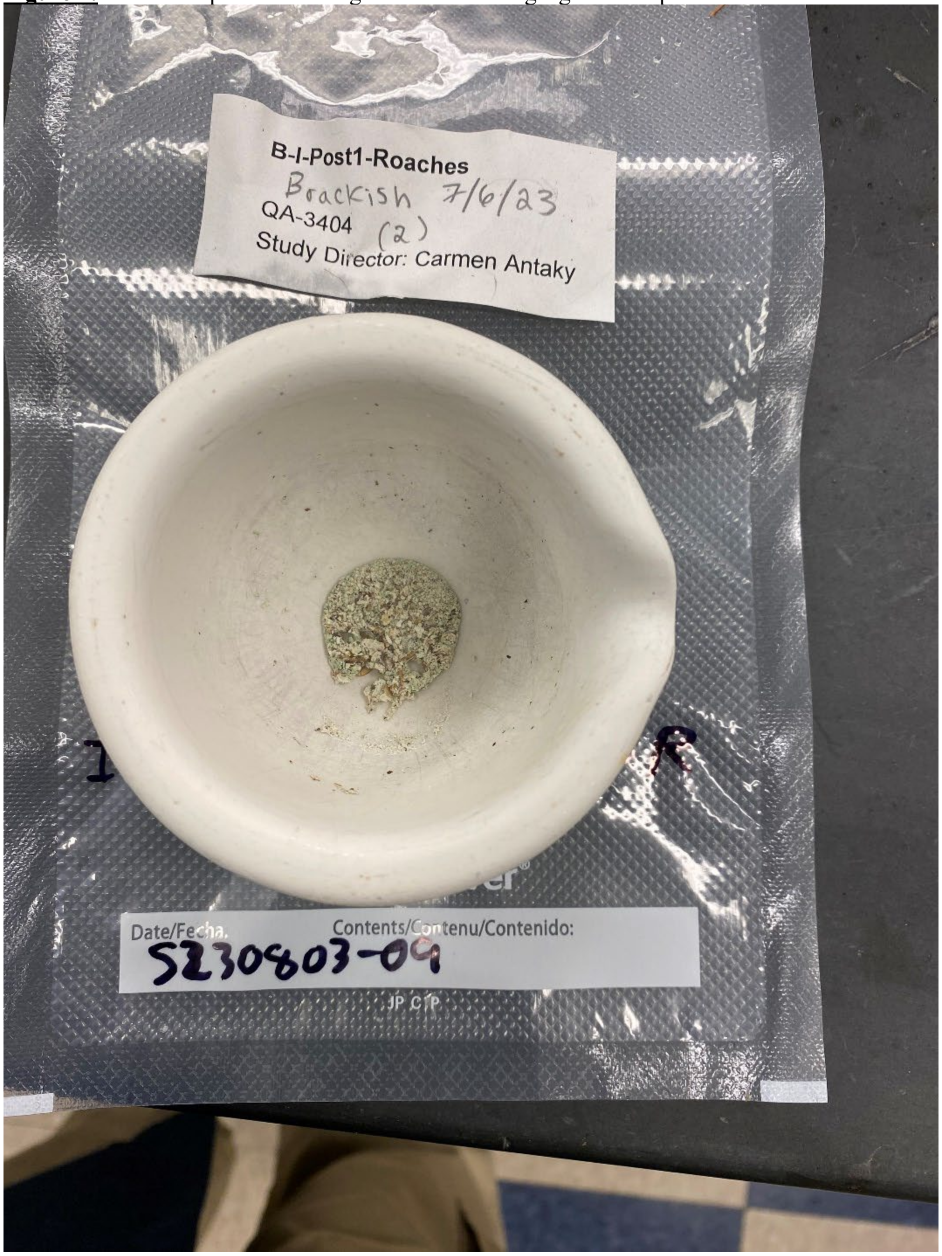

Appendix:

Figure 2: Homogenized roach sample S230803-09.

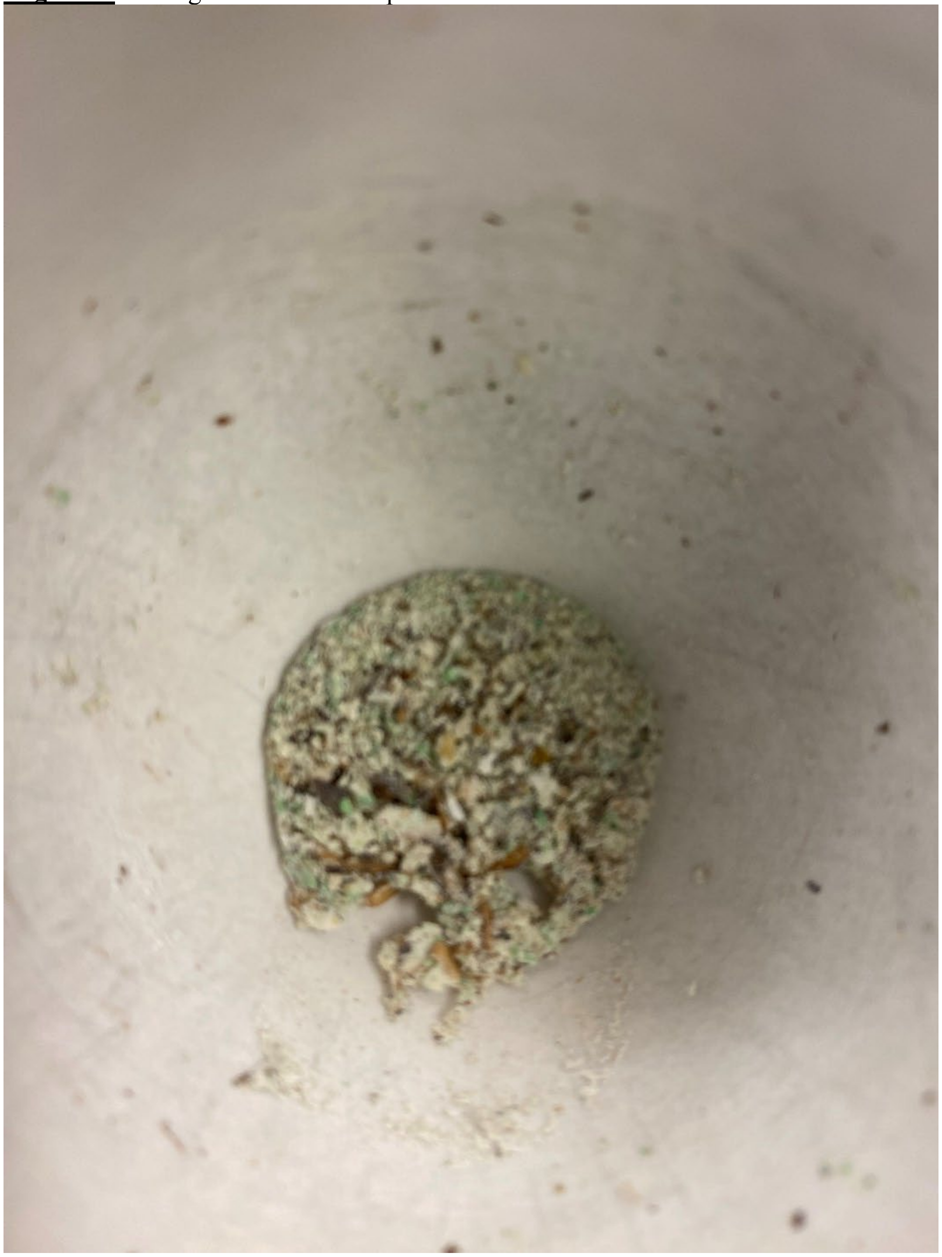

Appendix:

Figure 3: Roach sample S230803-10 showing the presence of a piece of bait material.

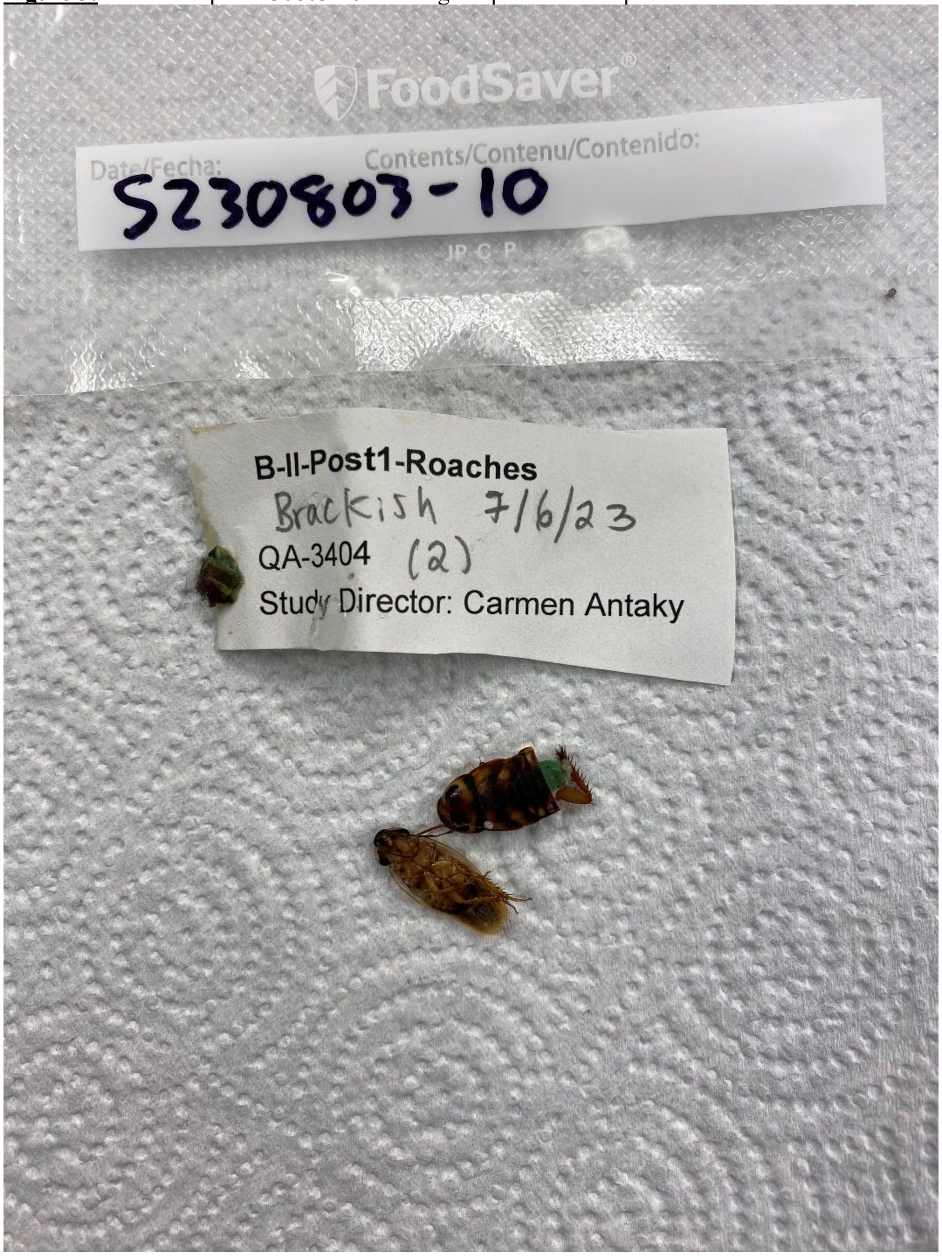

Appendix:

Figure 4: Roach sample S230803-10 showing the presence of a piece of bait material.

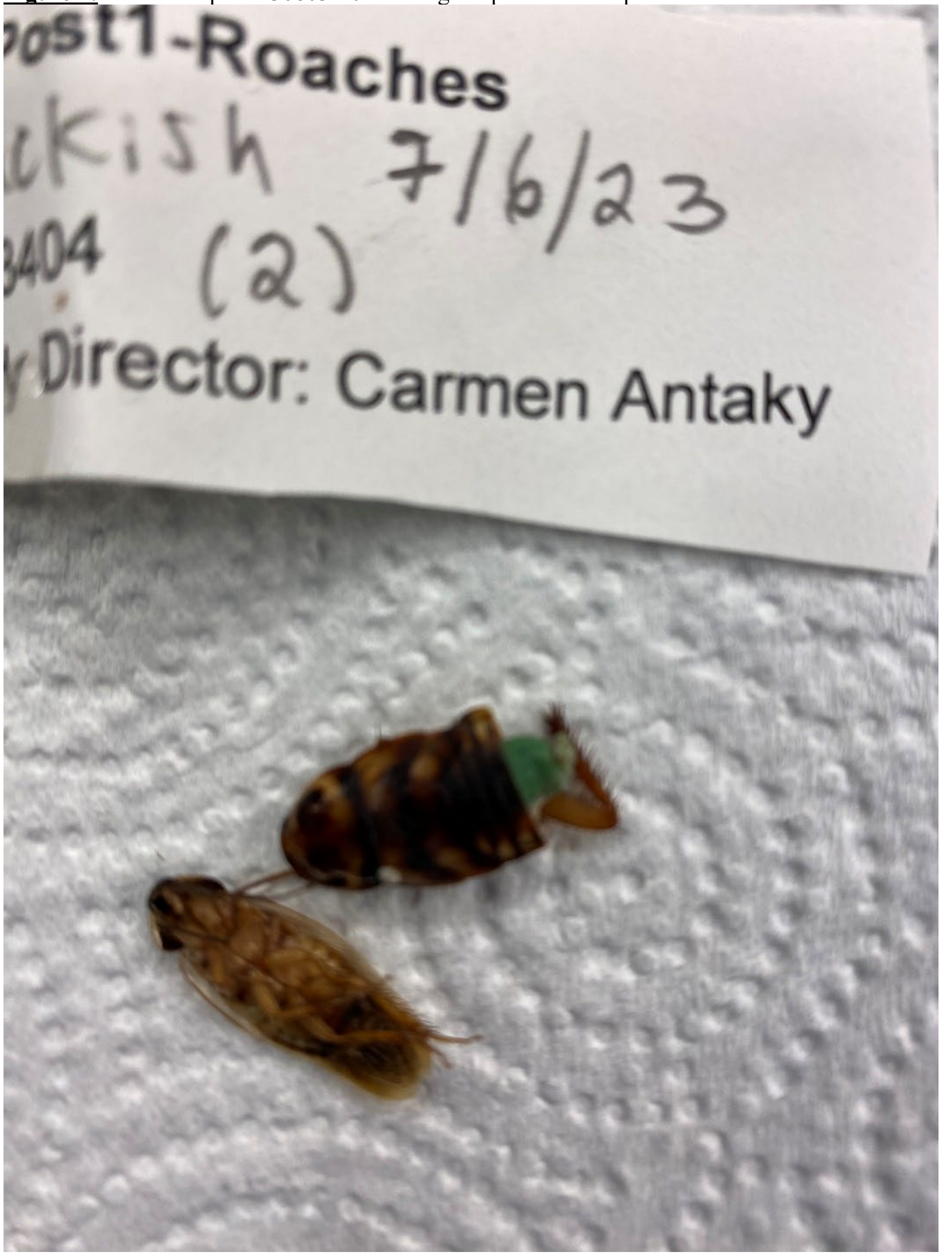

Appendix:

Figure 5: Roach sample S230803-10 showing the presence of a piece of bait material.

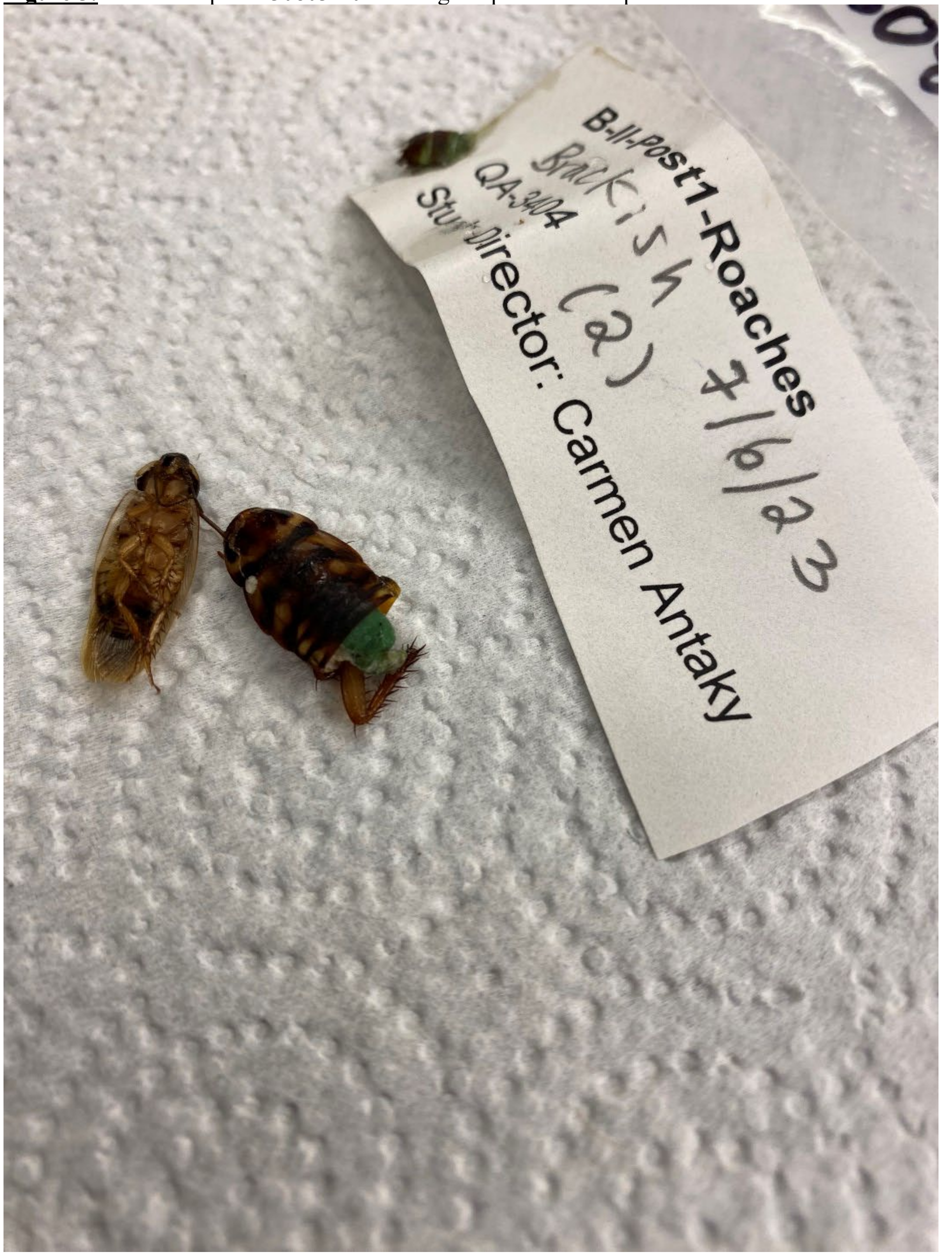

Appendix:

Figure 6: Roach sample S230803-10 showing the presence of a piece of bait material.

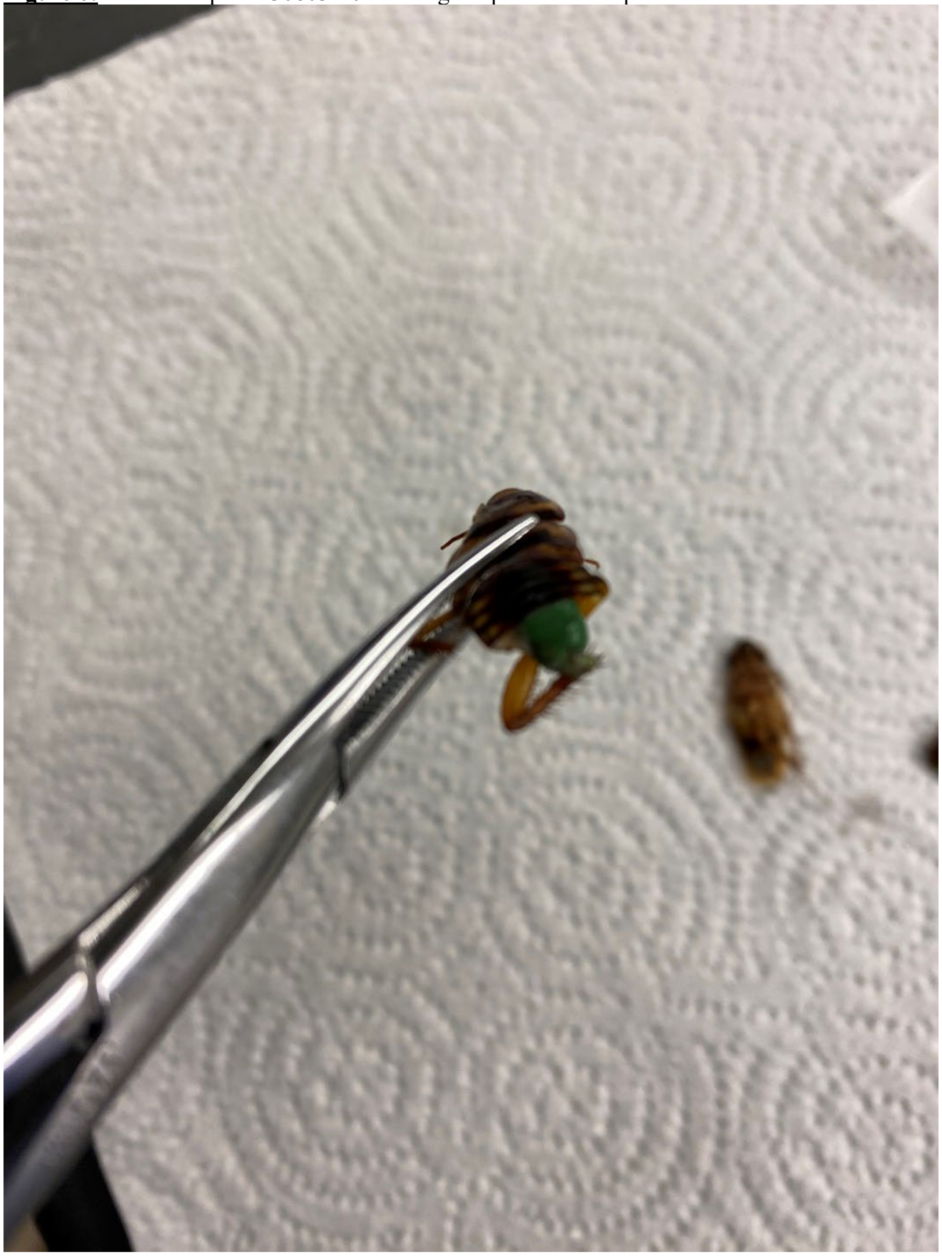

Supplement: S1 Appendix — (ZIP) [file pone.0344972.s001.zip › Supporting Information S1/23-021 Post 1 Midway Island Invertebrates Brodifacoum Report.pdf]
