# Supplementary material for: Anticoagulant residues associated with an attempted rodent eradication from a subtropical coral atoll
Source: PLoS One. 2026 Mar 23;21(3):e0344972. doi: 10.1371/journal.pone.0344972 (PMC13008109; doi:10.1371/journal.pone.0344972)
Supplement: S1 Appendix — (ZIP) [file pone.0344972.s001.zip › Supporting Information S1/23-021 Post 3 Midway Island Invertebrates Brodifacoum Report.pdf]

To:	Carmen Antaky<br>Biologist<br>NWRC Hawai'i Field Station
Subject:	Determination of brodifacoum in various matrices from Midway Island (QA-3404)
Methods:	188A-"Determination of Multiple Rodenticide Residues in Avian Liver by dSPE and LC-MS/MS" -Non-GLP
Analysis Dates:	09/13/23
Notebook References:	AC165, pp.186-187, 202<br>AC169, pp.8-11<br>QC35, p.68
Analyst:	Ben Abbo

Sample Description:

Eighteen invertebrate samples were submitted on 09/05/23. Nine invertebrate samples were submitted on 09/07/23. See sample descriptions on pp.3-5.

Additional Comments:

- The initial batch of eighteen invertebrate samples received on 09/05/23 had been held up during shipping and arrived at NWRC thawed and at room temperature. The samples were examined upon arrival and we determined that the roach samples (S230905-01 to -12) were still viable and processed for analysis. We determined that the pitfall samples (S230905-13 to -18) were degraded and would not yield useable data. We have retained the samples in the freezer.
- After processing, there was not enough of sample S230905-01 to analyze. This sample is marked as No Sample in the report.
- Samples S230905-02 to -12 had strong odors of decomposition, indicating that while the exoskeletons remained intact, the viscera had broken down. This led to having a smaller amount of sample tissue than anticipated. We decided to analyze a single replicate for these samples.
- Three replicates each of samples S230907-01 to -09 were analyzed unless there was insufficient sample to weigh out. These are marked as INS in the report.
- Control crickets (S221018-01) were used as the matrix for QC samples.

Method Limit of Detection/Quantitation (MLOD/MLOQ) Values:

Method detection and quantitation limits were determined from by comparing the noise at the analyte retention in five unfortified control cricket samples to the peak height of brodifacoum in five control cricket samples fortified to ~50 ng/g brodifacoum. The detection limit was determined to be 3X the noise and the quantitation limit was determined to be 10X the noise found in the unfortified samples.

Method Limit of Detection (MLOD)

Matrix	Detection Limit
Invertebrates	1.4 ng/g

Method Limit of Quantitation (MLOQ)

Matrix	Quantitation Limit
Invertebrates	4.69 ng/g

Results:

Sample ID	Sample Description	Brodifacoum (ng/g)
S230905-01	Invertebrates, A-I-Post3b-R, A - Radar, Roaches, Blattodea (3), 8/22/2023	No Sample
S230905-02	Invertebrates, A-II-Post3b-R, A - Radar, Roaches, Blattodea (3), 8/22/2023	2.3*
S230905-03	Invertebrates, B-I-Post3b-R, B - Brackish, Roaches, Blattodea (3), 8/22/2023	12.8
S230905-04	Invertebrates, B-II-Post3b-R, B - Brackish, Roaches, Blattodea (3), 8/22/2023	9.85
S230905-05	Invertebrates, C-I-Post3b-R, C – Rusty Bucket, Roaches, Blattodea (3), 8/22/2023	42.4
S230905-06	Invertebrates, C-II-Post3b-R, C – Rusty Bucket, Roaches, Blattodea (3), 8/22/2023	41.4
S230905-07	Invertebrates, D-I-Post3b-R, D – Ballfield, Roaches, Blattodea (3), 8/22/2023	9.83
S230905-08	Invertebrates, D-II-Post3b-R, D – Ballfield, Roaches, Blattodea (3), 8/22/2023	4.90
S230905-09	Invertebrates, E-I-Post3b-R, E – Ballfield, Roaches, Blattodea (3), 8/22/2023	ND
S230905-10	Invertebrates, E-II-Post3b-R, E – Ballfield, Roaches, Blattodea (3), 8/22/2023	6.48
S230905-11	Invertebrates, F-I-Post3b-R, F – SE Corner Radar Hill, Roaches, Blattodea (3), 8/22/2023	13.7
S230905-12	Invertebrates, F-II-Post3b-R, F – SE Corner Radar Hill, Roaches, Blattodea (3), 8/22/2023	31.4

ND = Not Detected.
*-Value is below the method quantitation limit of 4.69 ng/g

Results:

Sample ID	Sample Description	Brodifacoum (ng/g)
S230905-13	Invertebrates, A-I-Post3-P, A - Radar, Pitfall, Pooled, 8/22/2023	No Sample
S230905-14	Invertebrates, A-II-Post3-P, A - Radar, Pitfall, Pooled, 8/22/2023	No Sample
S230905-15	Invertebrates, B-I-Post3-P, B - Brackish, Pitfall, Pooled, 8/22/2023	No Sample
S230905-16	Invertebrates, B-II-Post3-P, B - Brackish, Pitfall, Pooled, 8/22/2023	No Sample
S230905-17	Invertebrates, C-I-Post3-P, C – Rusty Bucket, Pitfall, Pooled, 8/22/2023	No Sample
S230905-18	Invertebrates, C-II-Post3-P, C – Rusty Bucket, Pitfall, Pooled, 8/22/2023	No Sample

Results:

**Sample ID**	**Sample Description**	**Brodifacoum (ng/g)**	Descriptive Statistics	
S230907-01-A	Invertebrates, A-III-Post3b-R, A - Radar,	4.0*	Avg_2=	3.7*
S230907-01-B	Roaches, Blattodea (3), 8/22/2023	3.3*	sd=	0.50
S230907-01-C		INS	cv=	14%
S230907-02-A	Invertebrates, B-III-Post3b-R, B - Brackish,	24.4	$Mean_3$=	23.5
S230907-02-B	Roaches, Blattodea (3), 8/22/2023	23.3	sd=	0.82
S230907-02-C		22.8	cv=	3.5%
S230907-03-A	Invertebrates, C-III-Post3b-R, C – Rusty	24.2	Value=	24.2
S230907-03-B	Bucket, Roaches, Blattodea (3), 8/22/2023	INS	sd=	-----
S230907-03-C		INS	cv=	-----
S230907-04-A	Invertebrates, D-III-Post3b-R, D – Ballfield,	8.34	Value=	8.34
S230907-04-B	Roaches, Blattodea (3), 8/22/2023	INS	sd=	-----
S230907-04-C		INS	cv=	-----
S230907-05-A	Invertebrates, E-III-Post3b-R, E – Parade	3.9*	Value=	3.9*
S230907-05-B	Ground, Roaches, Blattodea (3), 8/22/2023	INS	sd=	-----
S230907-05-C		INS	cv=	-----
S230907-06-A	Invertebrates, F-III-Post3b-R, F – SE Corner	9.11	$Mean_3$=	9.62
S230907-06-B	Radar Hill, Roaches, Blattodea (3),	10.4	sd=	0.69
S230907-06-C	8/22/2023	9.35	cv=	7.2%
S230907-07-A	Invertebrates, A-III-Post3-P, A - Radar,	36.5	$Mean_3$=	29.2
S230907-07-B	Pitfall, Pooled, 8/22/2023	24.4	sd=	6.4
S230907-07-C		26.8	cv=	22%
S230907-08-A	Invertebrates, B-III-Post3-P, B - Brackish,	33.8	$Mean_3$=	34.1
S230907-08-B	Pitfall, Pooled, 8/22/2023	35.3	sd=	1.1
S230907-08-C		33.1	cv=	3.2%
S230907-09-A	Invertebrates, C-III-Post3-P, C – Rusty	10.1	$Mean_3$=	9.92
S230907-09-B	Bucket, Pitfall, Pooled, 8/22/2023	10.5	sd=	0.69
S230907-09-C		9.15	cv=	7.0%

ND = Not Detected.
*-Value is below the method quantitation limit of 4.69 ng/g
INS = Insufficient Sample

QC Results:

ID	Theoretical Brodifacoum Concentration (ng/g)	Observed Brodifacoum Concentration (ng/g)	% Recovery	Descriptive Statistics	
QC-21	Control	ND	N/A	$Mean_3$=	ND
QC-22	Control	ND	N/A	sd=	-----
QC-23	Control	ND	N/A	cv=	-----
QC-24	48.2	47.1	97.7	$Mean_3$=	98.7%
QC-25	51.5	52.1	101	sd=	2.0%
QC-26	48.7	47.5	97.5	cv=	2.0%
QC-27	624	626	100	$Mean_3$=	101%
QC-28	628	642	102	sd=	1.2%
QC-29	548	550	100	cv=	1.2%
QC-30	2230	2270	102	$Mean_3$=	103%
QC-31	1990	2070	104	sd=	1.2%
QC-32	1950	2020	104	cv=	1.2%

ND = Not Detected.
