# Supplementary material for: Anticoagulant residues associated with an attempted rodent eradication from a subtropical coral atoll
Source: PLoS One. 2026 Mar 23;21(3):e0344972. doi: 10.1371/journal.pone.0344972 (PMC13008109; doi:10.1371/journal.pone.0344972)
Supplement: S1 Appendix — (ZIP) [file pone.0344972.s001.zip › Supporting Information S1/23-028 Post 1 Midway Island Crustaceans Brodifacoum Report.pdf]

To: Carmen Antaky
Biologist
NWRC Hawai'i Field Station

Subject: Determination of brodifacoum in crustaceans from Midway Island (QA-3404)

Methods: 188A "Determination of Multiple Rodenticide Residues in Avian Liver by dSPE and LC-MS/MS" -Non-GLP

Analysis Dates: 08/09/23

Notebook References: AC165, pp.186-187, 194-196
QC35, p.68

Analyst: Ben Abbo

Sample Description:

Six samples of crustaceans were submitted on 08/03/23. See sample descriptions on p.3.

Additional Comments:

- Three replicates of each sample were analyzed. The mean brodifacoum concentration and standard deviation are reported for each sample.
- A ghost crab sample from Midway Island (S220801-41) that had been previously determined to have no detectable levels of brodifacoum was used as the matrix for QC samples.
- In samples that were positive for brodifacoum, two other peaks were also observed. These peaks were not observed in the brodifacoum fortified QC samples nor were they seen in the field samples that did not have detectable levels of brodifacoum, indicating that these peaks are not inherently part of the matrix and are related to the uptake of brodifacoum by the field samples. Examination of the mass spectra for these peaks showed that these peaks were consistent with brodifacoum. These peaks may be metabolites of brodifacoum, although we were not able to identify which compounds these peaks represent. A chromatogram showing these peaks has been attached as an appendix to this report.

Method Limit of Detection/Quantitation (MLOD/MLOQ) Values:

Method detection and quantitation limits were determined from by comparing the noise at the analyte retention in five unfortified control crustacean samples to the peak height of brodifacoum in five control crustacean samples fortified to ~50 ng/g brodifacoum. The detection limit was determined to be 3X the noise and the quantitation limit was determined to be 10X the noise found in the unfortified samples.

Method Limit of Detection (MLOD)

Matrix	Detection Limit
Crustaceans	1.3 ng/g

Method Limit of Quantitation (MLOQ)

Matrix	Quantitation Limit
Crustaceans	4.25 ng/g

Results:

Sample ID	Sample Description	Brodifacoum Conc (ng/g)	Descriptive Statistics	
S230803-38 A	Crustaceans, A-I-Post1-Gh, A – Cargo Pier,	ND		
S230803-38 B	Ghostcrabs, Ocypode pallidula, 7/4/2023	ND		
S230803-38 C		ND		
S230803-39 A	Crustaceans, A-II-Post1-Gh, A – Cargo Pier,	ND		
S230803-39 B	Ghostcrabs, Ocypode pallidula, 7/4/2023	ND		
S230803-39 C		ND		
S230803-40 A	Crustaceans, B-I-Post1-Gh, B – Hale Honu,	37.8	$Mean_3$=	41.1
S230803-40 B	Ghostcrabs, Ocypode pallidula, 7/4/2023	42.8	sd=	2.8
S230803-40 C		42.6	cv=	6.8%
S230803-41 A	Crustaceans, B-II-Post1-Gh, B – Hale Honu,	33.6	$Mean_3$=	34.5
S230803-41 B	Ghostcrabs, Ocypode pallidula, 7/4/2023	34.9	sd=	0.75
S230803-41 C		34.9	cv=	2.2%
S230803-42 A	Crustaceans, C-I-Post1-Gh, C – Rusty	19.6	$Mean_3$=	20.2
S230803-42 B	Bucket, Ghostcrabs, Ocypode pallidula,	20.4	sd=	0.53
S230803-42 C	7/4/2023	20.6	cv=	2.6%
S230803-43 A	Crustaceans, C-II-Post1-Gh, C – Rusty	6.13	$Mean_3$=	6.13
S230803-43 B	Bucket, Ghostcrabs, Ocypode pallidula,	6.96	sd=	0.84
S230803-43 C	7/4/2023	5.29	cv=	14%

ND = Not Detected

QC Results:

ID	Theoretical Brodifacoum Concentration (ng/g)	Observed Brodifacoum Concentration (ng/g)	% Recovery	Descriptive Statistics	
QC-01	Control	ND	N/A		
QC-02	Control	ND	N/A	$Mean_5$=	ND
QC-03	Control	ND	N/A	sd=	-----
QC-04	Control	ND	N/A	cv=	-----
QC-05	Control	ND	N/A		
QC-06	59.4	57.8	97.3		
QC-07	44.5	43.6	98.0	$Mean_5$=	97.9%
QC-08	46.9	44.7	95.3	sd=	2.0%
QC-09	48.8	49.3	101	cv=	2.0%
QC-10	48.0	47.1	98.1		
QC-11	565	551	97.5		
QC-12	563	557	98.9	$Mean_5$=	98.5%
QC-13	498	494	99.2	sd=	0.81%
QC-14	549	544	99.1	cv=	0.82%
QC-15	571	558	97.7		
QC-16	2030	2000	98.5		
QC-17	1900	1910	101	$Mean_5$=	99.1%
QC-18	1910	1900	99.5	sd=	1.3%
QC-19	1960	1940	99.0	cv=	1.3%
QC-20	2300	2240	97.4		

ND = Not Detected

Appendix:

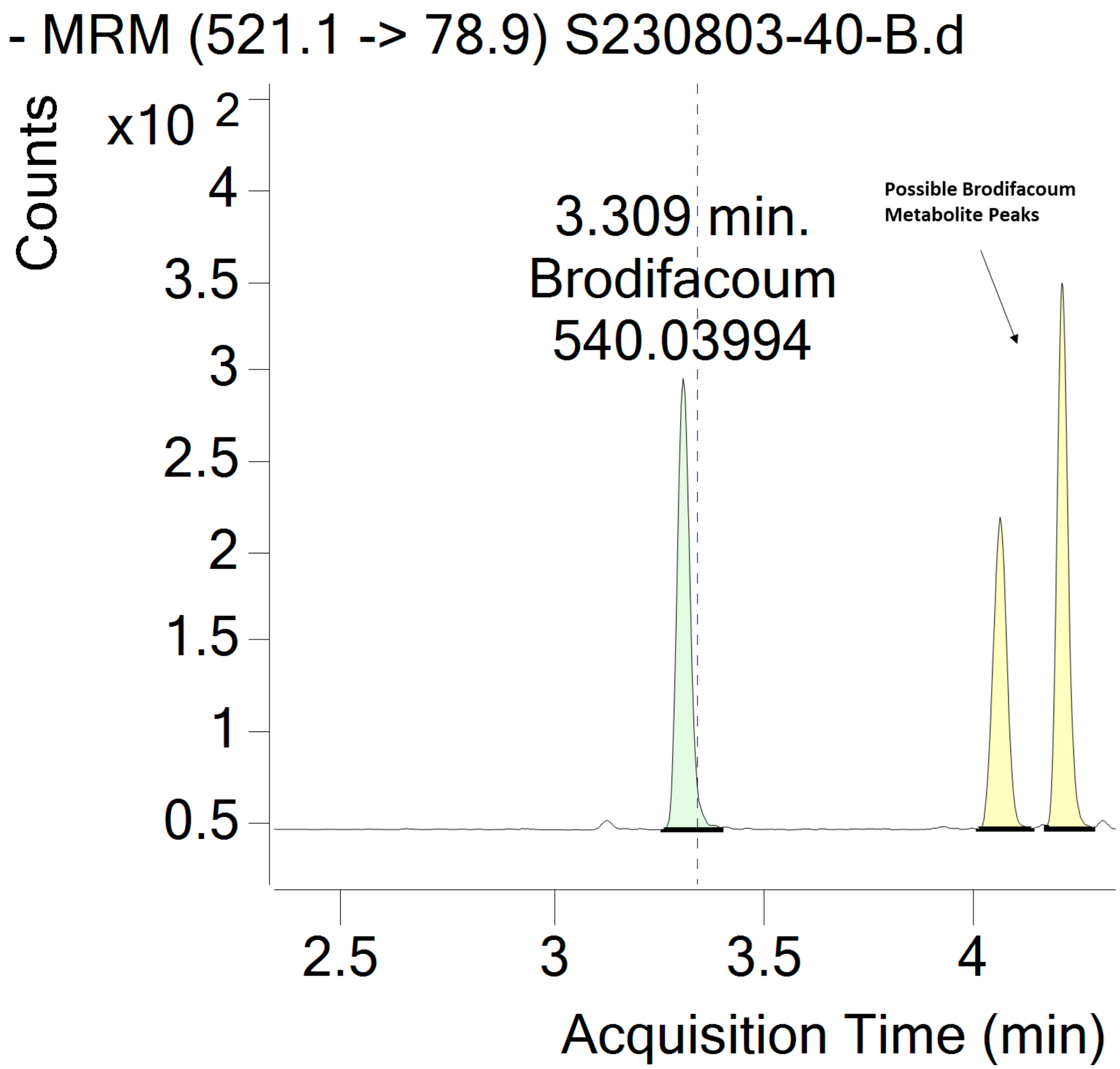

Figure 1: Chromatogram showing Brodifacoum (green peak) and possible metabolite peaks (yellow peaks)
