# Supplementary material for: Anticoagulant residues associated with an attempted rodent eradication from a subtropical coral atoll
Source: PLoS One. 2026 Mar 23;21(3):e0344972. doi: 10.1371/journal.pone.0344972 (PMC13008109; doi:10.1371/journal.pone.0344972)
Supplement: S1 Appendix — (ZIP) [file pone.0344972.s001.zip › Supporting Information S1/23-029 Post 1 Midway Island Reptiles Brodifacoum Report.pdf]

To: Carmen Antaky
Biologist
NWRC Hawai'i Field Station

Subject: Determination of brodifacoum in reptiles from Midway Island (QA-3404)

Methods: 188A "Determination of Multiple Rodenticide Residues in Avian Liver by dSPE and LC-MS/MS" -Non-GLP

Analysis Dates: 08/11/23

Notebook References: AC165, pp.186-187, 197-199
QC35, p.68

Analyst: Ben Abbo

Sample Description:

Six samples of reptiles were submitted on 08/03/23. See sample descriptions on p.3.

Additional Comments:

- Three replicates of each sample were analyzed. The mean brodifacoum concentration and standard deviation are reported for each sample.
- A reptile sample from Midway Island (S220801-47) that had been previously determined to have no detectable levels of brodifacoum was used as the matrix for QC samples.
- In samples S230803-44 and -48, two other peaks were also observed. These peaks were not observed in the brodifacoum fortified QC samples nor were they seen in the other field samples that had much lower levels of brodifacoum, indicating that these peaks are not inherently part of the matrix and are related to the uptake of brodifacoum by the field samples. These peaks were likely not seen in the other samples that were positive for brodifacoum due to the low concentration of brodifacoum in these samples. Examination of the mass spectra for these peaks showed that these peaks were consistent with brodifacoum. These peaks may be metabolites of brodifacoum, although we were not able to identify which compounds these peaks represent. A chromatogram showing these peaks has been attached as an appendix to this report.

Method Limit of Detection/Quantitation (MLOD/MLOQ) Values:

Method detection and quantitation limits were determined from by comparing the noise at the analyte retention in five unfortified control reptile samples to the peak height of brodifacoum in five control reptile samples fortified to ~50 ng/g brodifacoum. The detection limit was determined to be 3X the noise and the quantitation limit was determined to be 10X the noise found in the unfortified samples.

Method Limit of Detection (MLOD)

Matrix	Detection Limit
Reptiles	1.0 ng/g

Method Limit of Quantitation (MLOQ)

Matrix	Quantitation Limit
Reptiles	3.46 ng/g

Results:

Sample ID	Sample Description	Brodifacoum Conc (ng/g)	Descriptive Statistics	
S230803-44 A	Reptiles, A-I-Post1-Ge, A – Clipper, Geckos,	169	$Mean_3$=	161
S230803-44 B	House gecko, 7/5/2023	153	sd=	8.0
S230803-44 C		161	cv=	5.0%
S230803-45 A	Reptiles, A-II-Post1-Ge, A – Clipper,	4.06	$Mean_3$=	3.92
S230803-45 B	Geckos, House gecko, 7/5/2023	3.74	sd=	0.16
S230803-45 C		3.96	cv=	4.1%
S230803-46 A	Reptiles, B-I-Post1-Ge, B – Gym/Store,	6.51	$Mean_3$=	6.67
S230803-46 B	Geckos, House gecko, 7/5/2023	6.40	sd=	0.38
S230803-46 C		7.10	cv=	5.7%
S230803-47 A	Reptiles, B-II-Post1-Ge, B – Gym/Store,	11.4	$Mean_3$=	11.6
S230803-47 B	Geckos, House gecko, 7/5/2023	11.3	sd=	0.38
S230803-47 C		12.0	cv=	3.3%
S230803-48 A	Reptiles, C-I-Post1-Ge, C – FWS Office,	137	$Mean_3$=	143
S230803-48 B	Geckos, House gecko, 7/5/2023	138	sd=	9.0
S230803-48 C		153	cv=	6.3%
S230803-49 A	Reptiles, C-II-Post1-Ge, C – FWS Office,	3.3*	$Mean_3$=	3.51
S230803-49 B	Geckos, House gecko, 7/5/2023	3.53	sd=	0.20
S230803-49 C		3.70	cv=	5.7%

ND = Not Detected
* = Value is below the method quantitation limit of 3.46 ng/g

QC Results:

ID	Theoretical Brodifacoum Concentration (ng/g)	Observed Brodifacoum Concentration (ng/g)	% Recovery	Descriptive Statistics	
QC-01	Control	ND	N/A		
QC-02	Control	ND	N/A	$Mean_5$=	ND
QC-03	Control	ND	N/A	sd=	-----
QC-04	Control	ND	N/A	cv=	-----
QC-05	Control	ND	N/A		
QC-06	52.0	51.0	98.1		
QC-07	45.5	44.0	96.7	$Mean_5$=	96.3%
QC-08	46.9	44.0	93.8	sd=	1.7%
QC-09	45.6	43.6	95.6	cv=	1.8%
QC-10	52.9	51.4	97.2		
QC-11	517	516	99.8		
QC-12	592	582	98.3	$Mean_5$=	98.3%
QC-13	584	564	96.6	sd=	1.2%
QC-14	568	561	98.8	cv=	1.2%
QC-15	595	582	97.8		
QC-16	2140	2100	98.1		
QC-17	2390	2370	99.2	$Mean_5$=	98.7%
QC-18	1880	1870	99.5	sd=	0.62%
QC-19	1940	1910	98.5	cv=	0.63%
QC-20	2240	2200	98.2		

ND = Not Detected

Appendix:

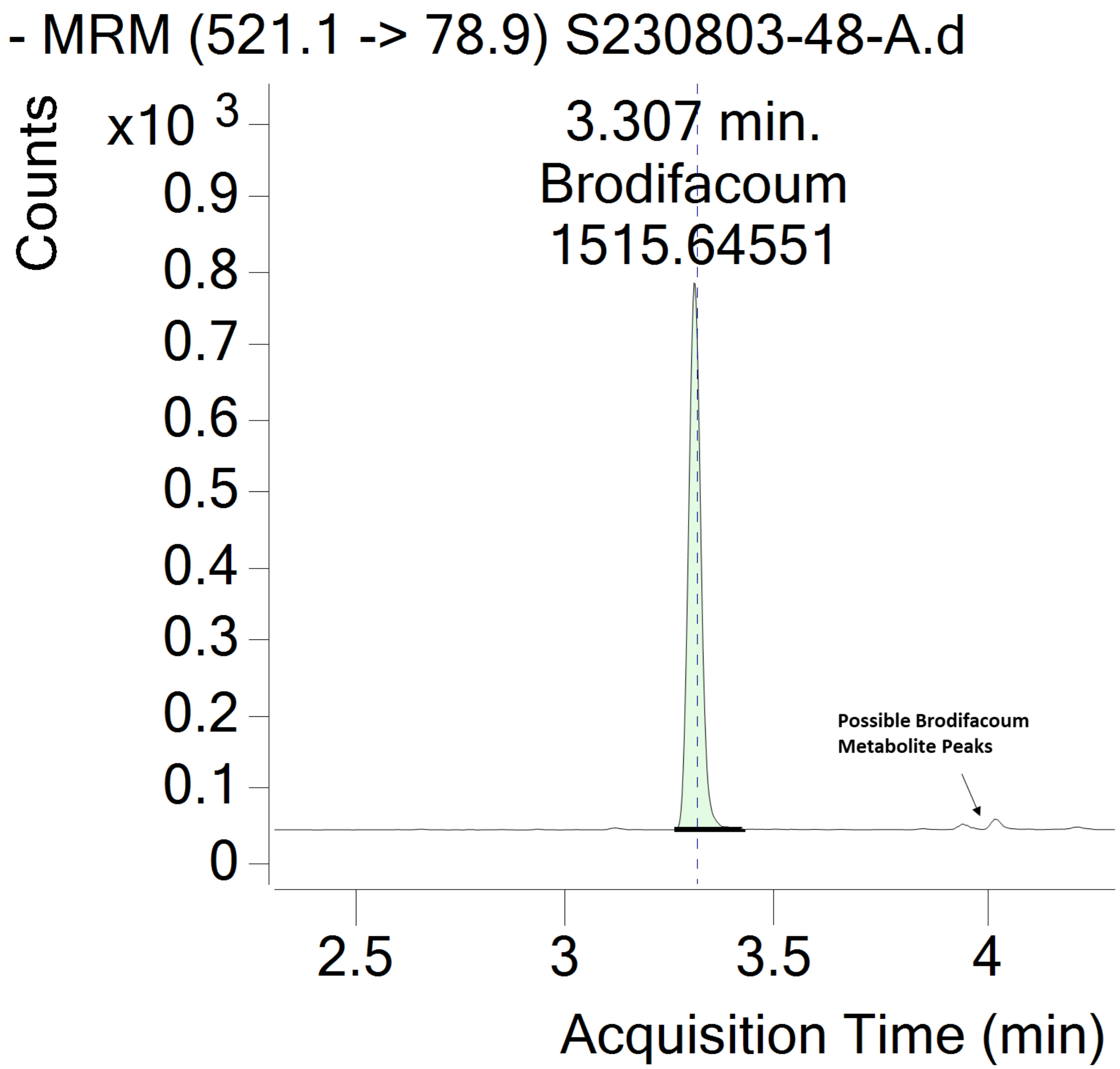

Figure 1: Chromatogram showing Brodifacoum (green peak) and possible metabolite peaks (yellow peaks)
