# Supplementary material for: Anticoagulant residues associated with an attempted rodent eradication from a subtropical coral atoll
Source: PLoS One. 2026 Mar 23;21(3):e0344972. doi: 10.1371/journal.pone.0344972 (PMC13008109; doi:10.1371/journal.pone.0344972)
Supplement: S1 Appendix — (ZIP) [file pone.0344972.s001.zip › Supporting Information S1/23-030 Post 1 Midway Island Fish Brodifacoum Report.pdf]

Wildlife Services NWRC National Wildlife Research Center<br>Analytical Services Report	United States Department of Agriculture<br>Animal Plant Health Inspection Service<br>Wildlife Services<br>National Wildlife Research Center<br>Laboratory Support Services Unit	Invoice #: 23-030<br>Date: 08/29/2023<br>Page: 1 of 5

To: Carmen Antaky
Biologist
NWRC Hawai'i Field Station

Subject: Determination of brodifacoum in fish matrices from Midway Island (QA-3404)

Methods: 188A "Determination of Multiple Rodenticide Residues in Avian Liver by dSPE and LC-MS/MS" -Non-GLP

Analysis Dates: 08/16/23

Notebook References: AC165, pp.186-187, 200-203
QC35, p.68

Analyst: Ben Abbo

Sample Description:

Seventeen fish samples were submitted on 08/03/23. See sample descriptions on pp.3-4.

Additional Comments:

- Three replicates of each sample were analyzed unless there was insufficient sample to weigh out three. These are marked as INS in the report. The mean brodifacoum concentration and standard deviation are reported for each sample, with the exceptions of samples S230803-22 and -37. These samples only had sufficient sample to run a single sample.
- Control Chilean Sea Bass (S220906-01) was used as the matrix for QC samples.

Method Limit of Detection/Quantitation (MLOD/MLOQ) Values:

Method detection and quantitation limits were determined by comparing the noise at the analyte retention in five unfortified control fish samples to the peak height of brodifacoum in five control fish samples fortified to ~50 ng/g brodifacoum. The detection limit was determined to be 3X the noise and the quantitation limit was determined to be 10X the noise found in the unfortified samples.

Method Limit of Detection (MLOD)

Matrix	Detection Limit
Fish	1.3 ng/g

Method Limit of Quantitation (MLOQ)

Matrix	Quantitation Limit
Fish	4.32 ng/g

Results:

Sample ID	Sample Description	Brodifacoum Conc (ng/g)	Descriptive Statistics	
S230803-21-A	Fish, A-I-Post1-M, A – R2, Mosquito Fish,	ND	Avg_2=	ND
S230803-21-B	Gambusia, 7/5/2023	ND	sd=	------
S230803-21-C		INS	cv=	------
S230803-22-A	Fish, A-II-Post1-M, A – R2, Mosquito Fish,	ND	Value=	ND
S230803-22-B	Gambusia, 7/5/2023	INS	sd=	------
S230803-22-C		INS	cv=	------
S230803-23-A	Fish, B-I-Post1-M, B - Catchment,	11.3	Avg_2=	11.8
S230803-23-B	Mosquito Fish, Gambusia, 7/5/2023	12.3	sd=	0.71
S230803-23-C		INS	cv=	6.0%
S230803-24-A	Fish, B-II-Post1-M, B - Catchment,	ND	$Mean_3$=	ND
S230803-24-B	Mosquito Fish, Gambusia, 7/5/2023	ND	sd=	------
S230803-24-C		ND	cv=	------
S230803-25-A	Fish, A-I-Post1-Bait, A – Cargo Pier, Bait	48.6	$Mean_3$=	48.9
S230803-25-B	Fish, Flagtail, 7/4/2023	46.5	sd=	2.5
S230803-25-C		51.5	cv=	5.1%
S230803-26-A	Fish, A-II-Post1-Bait, A – Cargo Pier, Bait	ND	$Mean_3$=	ND
S230803-26-B	Fish, Flagtail, 7/4/2023	ND	sd=	------
S230803-26-C		ND	cv=	------
S230803-27-A	Fish, B-I-Post1-Bait, B – Hale Honu, Bait	80.2	$Mean_3$=	77.4
S230803-27-B	Fish, Flagtail, 7/4/2023	76.5	sd=	2.5
S230803-27-C		75.5	cv=	3.2%
S230803-28-A	Fish, B-II-Post1-Bait, B – Hale Honu, Bait	72.0	$Mean_3$=	71.5
S230803-28-B	Fish, Flagtail, 7/4/2023	71.3	sd=	0.47
S230803-28-C		71.1	cv=	0.66%
S230803-29-A	Fish, C-I-Post1-Reefv2, C – Rusty Bucket,	2.7*	$Mean_3$=	3.2*
S230803-29-B	Bait Fish, Goatfish, 7/3/2023	3.7*	sd=	0.50
S230803-29-C		3.1*	cv=	16%
S230803-30-A	Fish, A-I-Post1-Reef, A – Cargo Pier, Reef	ND	$Mean_3$=	ND
S230803-30-B	Fish, Moi, 7/4/2023	ND	sd=	------
S230803-30-C		ND	cv=	------
S230803-31-A	Fish, A-II-Post1-Reef, A – Cargo Pier, Reef	ND	$Mean_3$=	ND
S230803-31-B	Fish, Moi, 7/4/2023	ND	sd=	------
S230803-31-C		ND	cv=	------
S230803-32-A	Fish, B-I-Post1-Reef, B – Hale Honu, Reef	ND	$Mean_3$=	ND
S230803-32-B	Fish, Goatfish, 7/4/2023	ND	sd=	------
S230803-32-C		ND	cv=	------

ND = Not Detected
* = Value is below the method quantitation limit of 4.32 ng/g
INS = Insufficient Sample

Results:

Sample ID	Sample Description	Brodifacoum (ng/g)	Descriptive Statistics	
S230803-33-A	Fish, B-II-Post1-Reef, B – Hale Honu, Reef	ND	$Mean_3$=	ND
S230803-33-B	Fish, Goatfish, 7/4/2023	ND	sd=	------
S230803-33-C		ND	cv=	------
S230803-34-A	Fish, C-I-Post1-Reef, C – Rusty Bucket,	ND	$Mean_3$=	ND
S230803-34-B	Reef Fish, Moi, 7/3/2023	ND	sd=	------
S230803-34-C		ND	cv=	------
S230803-35-A	Fish, C-II-Post1-Reef, C – Rusty Bucket,	ND	$Mean_3$=	ND
S230803-35-B	Reef Fish, Moi, 7/3/2023	ND	sd=	------
S230803-35-C		ND	cv=	------
S230803-36-A	Fish, A-IV-Post1-Con, R2, Freshwater fish	ND	Avg_2=	ND
S230803-36-B	(dead), Mosquitofish, 7/5/2023	ND	sd=	------
S230803-36-C		INS	cv=	------
S230803-37-A	Fish, A-V-Post1-Con, R2, Freshwater fish	ND	Value=	ND
S230803-37-B	(dead), Mosquitofish, 7/5/2023	INS	sd=	------
S230803-37-C		INS	cv=	------

ND = Not Detected
INS = Insufficient Sample

QC Results:

ID	Theoretical Brodifacoum Concentration (ng/g)	Observed Brodifacoum Concentration (ng/g)	% Recovery	Descriptive Statistics	
QC-01	Control	ND	N/A		
QC-02	Control	ND	N/A		
QC-03	Control	ND	N/A		
QC-04	Control	ND	N/A		
QC-05	Control	ND	N/A		
QC-06	44.7	41.9	93.7		
QC-07	52.0	50.9	97.9	$Mean_5$=	95.8%
QC-08	53.2	50.6	95.1	sd=	1.9%
QC-09	48.1	47.0	97.7	cv=	2.0%
QC-10	49.7	47.0	94.6		
QC-11	577	556	96.4		
QC-12	546	527	96.5	$Mean_5$=	96.3%
QC-13	555	536	96.6	sd=	0.40%
QC-14	586	560	95.6	cv=	0.42%
QC-15	572	551	96.3		
QC-16	2140	2040	95.3		
QC-17	2180	2090	95.9	$Mean_5$=	95.5%
QC-18	2260	2150	95.1	sd=	0.46%
QC-19	1990	1890	95.0	cv=	0.48%
QC-20	2260	2170	96.0		

ND = Not Detected
