# Supplementary material for: Anticoagulant residues associated with an attempted rodent eradication from a subtropical coral atoll
Source: PLoS One. 2026 Mar 23;21(3):e0344972. doi: 10.1371/journal.pone.0344972 (PMC13008109; doi:10.1371/journal.pone.0344972)
Supplement: S1 Appendix — (ZIP) [file pone.0344972.s001.zip › Supporting Information S1/23-031 Post 1 Midway Island Avian Livers Brodifacoum Report.pdf]

Wildlife Services **NWRC** National Wildlife Research Center<br>Analytical Services Report	United States Department of Agriculture<br>Animal Plant Health Inspection Service<br>Wildlife Services<br>National Wildlife Research Center<br>Laboratory Support Services Unit	Invoice #: 23-031<br>Date: 08/29/2023<br>Page: 1 of 4

To: Carmen Antaky
Biologist
NWRC Hawai'i Field Station

Subject: Determination of brodifacoum in avian livers from Midway Island (QA-3404)

Methods: 188A "Determination of Multiple Rodenticide Residues in Avian Liver by dSPE and LC-MS/MS" -Non-GLP

Analysis Dates: 08/21/23

Notebook References: AC165, pp.186-187, 204-206
QC35, p.68

Analyst: Ben Abbo

<u>Sample Description:</u>

Eight samples of birds or bird livers were submitted on 08/03/23. See sample descriptions on p.3.

<u>Additional Comments:</u>

- Necropsies were performed on carcass samples to recover the liver for analysis.
- Three replicates of each sample were analyzed, except when there was not sufficient sample for three replicates as noted in the report. The mean brodifacoum concentration and standard deviation are reported for each sample.
- Control quail liver (S221018-03) was used as the matrix for QC samples.

Method Limit of Detection/Quantitation (MLOD/MLOQ) Values:

Method detection and quantitation limits were determined from by comparing the noise at the analyte retention in five unfortified control avian liver samples to the peak height of brodifacoum in five control avian liver samples fortified to ~50 ng/g brodifacoum. The detection limit was determined to be 3X the noise and the quantitation limit was determined to be 10X the noise found in the unfortified samples.

Method Limit of Detection (MLOD)

Matrix	Detection Limit
Avian Livers	1.5 ng/g

Method Limit of Quantitation (MLOQ)

Matrix	Quantitation Limit
Avian Livers	4.97 ng/g

Results:

Sample ID	Sample Description	Brodifacoum Conc (ng/g)	Descriptive Statistics	
S230803-64-A	Duck Carcasses, A-I-Post1-Du, Catchment	1140	$Mean_3$=	1160
S230803-64-B	(died in captivity), Duck, Liver (Laysan	1150	sd=	21
S230803-64-C	Duck) LADU-23-061, 7/6/2023	1180	cv=	1.8%
S230803-65-A	Shorebird Carcass, A-I-Post1-Sh,	465	$Mean_3$=	468
S230803-65-B	Catchment (died in captivity), Shorebird,	482	sd=	12
S230803-65-C	Liver (Pacific Golden Plover), 7/6/2023	458	cv=	2.6%
S230803-66-A	Myna Carcass, A-I-Post1-My, Empire Cafe,	384	$Mean_3$=	386
S230803-66-B	Sentinel Passerines, Myna, 7/4/2023	386	sd=	2.5
S230803-66-C		389	cv=	0.65%
S230803-67-A	Myna Carcass, A-II-Post1-My, Empire	1200	$Mean_3$=	1220
S230803-67-B	Cafe, Sentinel Passerines, Myna, 7/6/2023	1220	sd=	20
S230803-67-C		1240	cv=	1.6%
S230803-68-A	Egret carcass, A-III-Post1-Egret, Radar,	849	$Mean_3$=	860
S230803-68-B	Sentinel Passerines, Cattle Egret, 7/7/2023	872	sd=	12
S230803-68-C		860	cv=	1.4%
S230803-69-A	Canary Carcass, A-I-Post1-Ca, Radar (in	2910	Value=	2910
S230803-69-B	ironwoods), Sentinel Passerines, Canary,	INS	sd=	------
S230803-69-C	7/6/2023	INS	cv=	------
S230803-70-A	Seabird Carcass, A-I-Post1-Se, Rusty	2.1*	Avg_2=	2.0*
S230803-70-B	bucket, Seabird, White Tern Chick,	1.8*	sd=	0.21
S230803-70-C	7/9/2023	INS	cv=	11%
S230803-71-A	Seabird Carcass, A-II-Post1-Se, Empire,	ND	$Mean_3$=	ND
S230803-71-B	Seabird, LAAL chick liver, 7/8/2023	ND	sd=	------
S230803-71-C		ND	cv=	------

ND = Not Detected
* = Value is below the quantitation limit of 4.97 ng/g
INS = Insufficient Sample

QC Results:

ID	Theoretical Brodifacoum Concentration (ng/g)	Observed Brodifacoum Concentration (ng/g)	% Recovery	Descriptive Statistics	
QC-01	Control	ND	N/A		
QC-02	Control	ND	N/A	$Mean_5$=	ND
QC-03	Control	ND	N/A	sd=	-----
QC-04	Control	ND	N/A	cv=	-----
QC-05	Control	ND	N/A		
QC-06	49.6	46.7	94.2		
QC-07	49.9	49.1	98.4	$Mean_5$=	95.6%
QC-08	56.6	54.4	96.1	sd=	1.7%
QC-09	53.8	50.8	94.4	cv=	1.8%
QC-10	49.6	47.0	94.8		
QC-11	600	588	98.0		
QC-12	551	545	98.9	$Mean_5$=	98.0%
QC-13	578	561	97.1	sd=	0.70%
QC-14	516	504	97.7	cv=	0.71%
QC-15	528	520	98.5		
QC-16	2040	1990	97.5		
QC-17	2330	2280	97.9	$Mean_5$=	97.6%
QC-18	2370	2330	98.3	sd=	0.63%
QC-19	1970	1920	97.5	cv=	0.65%
QC-20	2030	1960	96.6		

ND = Not Detected
