# Supplementary material for: Anticoagulant residues associated with an attempted rodent eradication from a subtropical coral atoll
Source: PLoS One. 2026 Mar 23;21(3):e0344972. doi: 10.1371/journal.pone.0344972 (PMC13008109; doi:10.1371/journal.pone.0344972)
Supplement: S1 Appendix — (ZIP) [file pone.0344972.s001.zip › Supporting Information S1/23-031 Post 3 Midway Island Avian Livers Brodifacoum Report.pdf]

To: Carmen Antaky
Biologist
NWRC Hawai'i Field Station

Subject: Determination of brodifacoum in avian livers from Midway Island (QA-3404)

Methods: 188A "Determination of Multiple Rodenticide Residues in Avian Liver by dSPE and LC-MS/MS" -Non-GLP

Analysis Dates: 09/14/23

Notebook References: AC165, pp.186-187, 202
AC169, pp.12-14
QC35, p.68

Analyst: Ben Abbo

Sample Description:

Four avian liver samples were submitted on 09/05/23. Eight avian liver samples were submitted on 09/07/23. See sample descriptions on p.3.

Additional Comments:

- The initial batch of four avian liver samples received on 09/05/23 had been held up during shipping and arrived at NWRC thawed and at room temperature. We attempted to process some of the samples to analyze, but we were unable to recover sufficient sample to test. These samples are marked No Sample in the report.
- Three replicates of each sample were analyzed, except when there was not sufficient sample for three replicates as noted in the report. The mean brodifacoum concentration and standard deviation are reported for each sample.
- Control quail liver (S221018-03) was used as the matrix for QC samples.

Method Limit of Detection/Quantitation (MLOD/MLOQ) Values:

Method detection and quantitation limits were determined from by comparing the noise at the analyte retention in five unfortified control avian liver samples to the peak height of brodifacoum in five control avian liver samples fortified to ~50 ng/g brodifacoum. The detection limit was determined to be 3X the noise and the quantitation limit was determined to be 10X the noise found in the unfortified samples.

Method Limit of Detection (MLOD)

Matrix	Detection Limit
Avian Livers	1.5 ng/g

Method Limit of Quantitation (MLOQ)

Matrix	Quantitation Limit
Avian Livers	4.97 ng/g

Results:

Sample ID	Sample Description	Brodifacoum Conc (ng/g)	Descriptive Statistics	
S230905-19	Avian Liver, A-I-Post3-Du, Monument on Eastern, Duck, Laysan Duck liver, 8/16/2023	No Sample		
S230905-20	Avian Liver, A-II-Post3-Sh, N Jetty Harbor, Shorebird, Ruddy Turnstone liver, 8/18/2023	No Sample		
S230905-21	Avian Liver, A-IV-Post3-Sh, NAF Hangar, Shorebird, Ruddy Turnstone liver, 8/17/2023	No Sample		
S230905-22	Avian Liver, A-V-Post3-Sh, Catchment, Shorebird, Pacific Golden Plover liver, 8/14/2023	No Sample		
S230907-10-A	Avian Liver, A-II-Post3-Du, Monument on	1020	Value=	1020
S230907-10-B	Eastern, Duck, LADU liver (23-523),	INS	sd=	-----
S230907-10-C	8/16/2023	INS	cv=	-----
S230907-11-A	Avian Liver, B-I-Post3-Du, Eastern, Duck,	80.8	Avg_2=	81.2
S230907-11-B	LADU liver (23-272), 8/24/2023	81.6	sd=	0.57
S230907-11-C		INS	cv=	0.70%
S230907-12-A	Avian Liver, C-I-Post3-Du, Eastern, Duck,	249	Value=	249
S230907-12-B	LADU liver (23-630), 8/28/2023	INS	sd=	----
S230907-12-C		INS	cv=	-----
S230907-13-A	Avian Liver, A-II-Post3-Sh, N Jetty	889	Avg_2=	907
S230907-13-B	Harbor, Shorebird, RUTU liver, 8/18/2023	925	sd=	25
S230907-13-C		INS	cv=	2.8%
S230907-14-A	Avian Liver, A-IV-Post3-Sh, NF Hanger,	853	$Mean_3$=	866
S230907-14-B	Shorebird, RUTU liver, 8/17/2023	859	sd=	18
S230907-14-C		887	cv=	2.1%
S230907-15-A	Avian Liver, A-V-Post3-Sh, Catchment,	751	$Mean_3$=	744
S230907-15-B	Shorebird, PAGP liver, 8/14/2023	729	sd=	13
S230907-15-C		751	cv=	1.7%
S230907-16-A	Avian Liver, A-I-Post3-Egret,	942	Avg_2=	910
S230907-16-B	Chugach/transportation @ garden, Egret,	878	sd=	45
S230907-16-C	CAEG liver, 8/22/2023	INS	cv=	4.9%
S230907-17-A	Avian Liver, A-I-Post3-My, Parade ground,	723	Avg_2=	684
S230907-17-B	Passerine, COMA liver, 8/25/2023	644	sd=	56
S230907-17-C		INS	cv=	8.2%

ND = Not Detected
INS = Insufficient Sample

QC Results:

ID	Theoretical Brodifacoum Concentration (ng/g)	Observed Brodifacoum Concentration (ng/g)	% Recovery	Descriptive Statistics	
QC-21	Control	ND	N/A	$Mean_3$=	ND
QC-22	Control	ND	N/A	sd=	-----
QC-23	Control	ND	N/A	cv=	-----
QC-24	48.7	46.5	95.5	$Mean_3$=	93.9%
QC-25	53.3	49.3	92.5	sd=	1.5%
QC-26	61.0	57.1	93.6	cv=	1.6%
QC-27	580	570	98.3	$Mean_3$=	98.7%
QC-28	607	600	98.8	sd=	0.40%
QC-29	580	575	99.1	cv=	0.41%
QC-30	2280	2320	102	$Mean_3$=	103%
QC-31	2510	2580	103	sd=	1.0%
QC-32	1940	2020	104	cv=	0.97%

ND = Not Detected
