# Supplementary material for: Anticoagulant residues associated with an attempted rodent eradication from a subtropical coral atoll
Source: PLoS One. 2026 Mar 23;21(3):e0344972. doi: 10.1371/journal.pone.0344972 (PMC13008109; doi:10.1371/journal.pone.0344972)
Supplement: S1 Appendix — (ZIP) [file pone.0344972.s001.zip › Supporting Information S1/23-032 Post 1 Midway Island Soils Brodifacoum Report.pdf]

To: Carmen Antaky
Biologist
NWRC Hawai'i Field Station

Subject: Determination of brodifacoum in soils from Midway Island (QA-3404)

Methods: 188A "Determination of Multiple Rodenticide Residues in Avian Liver by dSPE and LC-MS/MS" -Non-GLP

Analysis Dates: 08/23/23, 08/28/23

Notebook References: AC165, pp.186-187, 202; AC169, pp.1-3, 6
QC35, p.68

Analyst: Ben Abbo

Sample Description:

Six samples of soil from Midway Atoll were submitted on 08/03/23. See sample descriptions on p.3.

Additional Comments:

- Three replicates of each sample were analyzed. The mean brodifacoum concentration and standard deviation are reported for each sample.
- Replicate S230803-51-C had a positive result for brodifacoum while the two other replicates for that sample had no detectable levels of brodifacoum. It is likely that this result is due to a contamination of the replicate during the extraction procedure and not a true positive result. Three further replicates of this sample were extracted on 08/28/23 to confirm this. These three replicates did not have detectable levels of brodifacoum and an outlier test determined that the result for S230803-51-C was an outlier and its result was disregarded. The sample was reported as Not Detected.
- A soil sample from Midway Atoll (S220801-61) that had been previously determined to have no detectable levels of brodifacoum was used as the matrix for QC samples.

Method Limit of Detection/Quantitation (MLOD/MLOQ) Values:

Method detection and quantitation limits were determined by comparing the noise at the analyte retention in five unfortified control soil samples to the peak height of brodifacoum in five control soil samples fortified to ~50 ng/g brodifacoum. The detection limit was determined to be 3X the noise and the quantitation limit was determined to be 10X the noise found in the unfortified samples.

Method Limit of Detection (MLOD)

Matrix	Detection Limit
Soil	1.3 ng/g

Method Limit of Quantitation (MLOQ)

Matrix	Quantitation Limit
Soil	4.33 ng/g

Results:

Sample ID	Sample Description	Analysis Date	Brodifacoum Conc (ng/g)	Descriptive Statistics	
S230803-50-A	Soil, A-I-Post1-S, A -Radar, Soil, 7/7/2023	08/23/23	ND	$Mean_3$=	ND
S230803-50-B		08/23/23	ND	sd=	------
S230803-50-C		08/23/23	ND	cv=	------
S230803-51-A	Soil, B-I-Post1-S, B – Brackish, Soil, 7/7/2023	08/23/23	ND	$Mean_3$=	ND
S230803-51-B		08/23/23	ND	sd=	------
S230803-51-C		08/23/23	4.86*	cv=	------
S230803-51-D		08/28/23	ND		
S230803-51-E		08/28/23	ND		
S230803-51-F		08/28/23	ND		
S230803-52-A	Soil, C-I-Post1-S, C – Community Garden, Soil, 7/6/2023	08/23/23	ND	$Mean_3$=	ND
S230803-52-B		08/23/23	ND	sd=	------
S230803-52-C		08/23/23	ND	cv=	------
S230803-53-A	Soil, A-I-Post1-SS, A -Parade Ground, Soil, 7/6/2023	08/23/23	ND	$Mean_3$=	ND
S230803-53-B		08/23/23	ND	sd=	------
S230803-53-C		08/23/23	ND	cv=	------
S230803-54-A	Soil, B-I-Post1-SS, B – Orchard, Soil, 7/6/2023	08/23/23	ND	$Mean_3$=	ND
S230803-54-B		08/23/23	ND	sd=	------
S230803-54-C		08/23/23	ND	cv=	------
S230803-55-A	Soil, C-I-Post1-SS, C – Community Garden, Soil, 7/6/2023	08/23/23	ND	$Mean_3$=	ND
S230803-55-B		08/23/23	ND	sd=	------
S230803-55-C		08/23/23	ND	cv=	------

ND = Not Detected
*-Value was determined to be an outlier and will be disregarded.

QC Results:

ID	Analysis Date	Theoretical Brodifacoum Concentration (ng/g)	Observed Brodifacoum Concentration (ng/g)	% Recovery	Descriptive Statistics	
QC-01	08/23/23	Control	ND	N/A		
QC-02	08/23/23	Control	ND	N/A	$Mean_5$=	ND
QC-03	08/23/23	Control	ND	N/A	sd=	-----
QC-04	08/23/23	Control	ND	N/A	cv=	-----
QC-05	08/23/23	Control	ND	N/A		
QC-06	08/23/23	54.4	54.0	99.3		
QC-07	08/23/23	50.5	49.1	97.2	$Mean_5$=	95.8%
QC-08	08/23/23	60.1	57.8	96.2	sd=	4.5%
QC-09	08/23/23	56.9	55.9	98.2	cv=	4.7%
QC-10	08/23/23	52.8	46.4	87.9		
QC-11	08/23/23	605	589	97.4		
QC-12	08/23/23	628	603	96.0	$Mean_5$=	96.4%
QC-13	08/23/23	676	656	97.0	sd=	0.71%
QC-14	08/23/23	590	565	95.8	cv=	0.74%
QC-15	08/23/23	628	603	96.0		
QC-16	08/23/23	2060	2020	98.1		
QC-17	08/23/23	2140	2070	96.7	$Mean_5$=	97.9%
QC-18	08/23/23	2310	2280	98.7	sd=	0.84%
QC-19	08/23/23	2050	2020	98.5	cv=	0.86%
QC-20	08/23/23	2240	2180	97.3		

ND = Not Detected
