# Supplementary material for: Anticoagulant residues associated with an attempted rodent eradication from a subtropical coral atoll
Source: PLoS One. 2026 Mar 23;21(3):e0344972. doi: 10.1371/journal.pone.0344972 (PMC13008109; doi:10.1371/journal.pone.0344972)
Supplement: S1 Appendix — (ZIP) [file pone.0344972.s001.zip › Supporting Information S1/23-033 Post 1 Midway Island Waters Brodifacoum Report.pdf]

Wildlife Services **NWRC** National Wildlife Research Center<br>Analytical Services Report	United States Department of Agriculture<br>Animal Plant Health Inspection Service<br>Wildlife Services<br>National Wildlife Research Center<br>Laboratory Support Services Unit	Invoice #: 23-033<br>Date: 08/31/2023<br>Page: 1 of 4

To:	Carmen Antaky<br>Biologist<br>NWRC Hawai'i Field Station
Subject:	Determination of brodifacoum in Waters from Midway Island (QA-3404)
Methods:	188A "Determination of Multiple Rodenticide Residues in Avian Liver by dSPE and LC-MS/MS" -Non-GLP<br>"Determination of Brodifacoum Residues in Water"-Non-GLP
Analysis Dates:	08/26/23, 08/29/23
Notebook References:	AC165, pp.186-187, 202; AC169, pp.4-5, 7<br>QC35, p.68
Analyst:	Ben Abbo

Sample Description:

Eight samples of water from Midway Atoll were submitted on 08/03/23. See sample descriptions on p.3.

Additional Comments:

- Two replicates of each sample were analyzed.
- Replicates S230803-58-A and -59-B had positive results for brodifacoum while the other replicates for those samples had no detectable levels of brodifacoum. It is likely that this result is due to a contamination of the replicate during the extraction procedure and not a true positive result. Two further replicates of each sample were extracted on 08/29/23 to confirm this. These two replicates did not have detectable levels of brodifacoum and an outlier test determined that the results for S230803-58-A and -59-B were outliers and their results were disregarded. The sample was reported as Not Detected.
- Cache la Poudre River water from Laporte, Colorado (S230825-01) was used as the matrix for QC samples for fresh water.
- Synthetic sea water (NWRC ID# 7262) was used as the matrix for QC samples for sea water.

Sample Preparation and Extraction:

Water Extraction Procedure:

1. Add 60 mL of seawater to a 125-mL separatory funnel.
2. Add 0.020 mL surrogate to all and 0.040 mL of acetonitrile or 75X brodifacoum stock as indicated.
3. Add 20 mL chloroform.
4. Add ~8.5g sodium chloride.
5. Add 10 mL 1M hydrochloric acid.
6. Cap and shake for 8-10s, let set 1 minute. Repeat 2X.
7. Dispense chloroform phase into 25-mL glass tube taking care to not transfer any water phase.
8. Remove solvent in a 60°C N-Evap with a gentle flow of nitrogen gas.
9. Add 0.300 mL acetonitrile, vortex thoroughly, wetting as much of the inside surface of the tube as possible.
10. Add 1.200 mL pH 9.5 20-mM ammonium acetate buffer and vortex thoroughly.
11. Transfer the sample to an autosampler vial and assay by LC-MS/MS.

Method Limit of Detection/Quantitation (MLOD/MLOQ) Values:

Method detection and quantitation limits were determined by comparing the noise at the analyte retention in five unfortified control water samples to the peak height of brodifacoum in five control water samples fortified to 0.192 ng/mL brodifacoum. The detection limit was determined to be 3X the noise and the quantitation limit was determined to be 10X the noise found in the unfortified samples.

Method Limit of Detection (MLOD)

Matrix	Detection Limit
Fresh water	0.0018 ng/mL
Sea water	0.0030 ng/mL

Method Limit of Quantitation (MLOQ)

Matrix	Quantitation Limit
Fresh water	0.00615 ng/mL
Sea water	0.00994 ng/mL

Results:

Sample ID	Sample Description	Analysis Date	Brodifacoum (ng/g)	Descriptive Statistics	
S230803-56-A	Water Collection, A-I-Post1-WC, A –	8/26/23	ND	Avg_2=	ND
S230803-56-B	Catchment, Water, 7/5/2023	8/26/23	ND	sd=	------
				cv=	------
S230803-57-A	Ocean water, A-I-Post1-Oc, A – Cargo Pier,	8/26/23	ND	Avg_2=	ND
S230803-57-B	Water, 7/4/2023	8/26/23	ND	sd=	------
				cv=	------
S230803-58-A	Ocean water, B-I-Post1-Oc, B – Hale Honu,	8/26/23	0.0269*	$Mean_3$=	ND
S230803-58-B	Water, 7/4/2023	8/26/23	ND	sd=	------
S230803-58-C		8/29/23	ND	cv=	------
S230803-58-D		8/29/23	ND		
S230803-59-A	Ocean water, C-I-Post1-Oc, C – Rusty	8/26/23	ND	$Mean_3$=	ND
S230803-59-B	Bucket, Water, 7/3/2023	8/26/23	0.0038*	sd=	------
S230803-59-C		8/29/23	ND	cv=	------
S230803-59-D		8/29/30	ND		
S230803-60-A	Ocean water, D-I-Post1-Oc, D - Harbor,	8/26/23	ND	Avg_2=	ND
S230803-60-B	Water, 7/4/2023	8/26/23	ND	sd=	------
				cv=	------
S230803-61-A	Fresh water, A-I-Post1-Fr, A – Radar,	8/26/23	ND	Avg_2=	ND
S230803-61-B	Water, 7/7/2023	8/26/23	ND	sd=	------
				cv=	------
S230803-62-A	Fresh water, B-I-Post1-Fr, B – Brackish,	8/26/23	ND	Avg_2=	ND
S230803-62-B	Water, 7/7/2023	8/26/23	ND	sd=	------
				cv=	------
S230803-63-A	Fresh water, C-I-Post1-Fr, C – R2, Water,	8/26/23	ND	Avg_2=	ND
S230803-63-B	7/5/2023	8/26/23	ND	sd=	------
				cv=	------

ND = Not Detected
*-Value was determined to be an outlier and will be disregarded.

QC Results:

Fresh Water:

ID	Analysis Date	Theoretical Brodifacoum Concentration (ng/mL)	Observed Brodifacoum Concentration (ng/mL)	% Recovery
QC-01	8/26/23	Control	ND	N/A
QC-02	8/26/23	Control	ND	N/A
QC-03	8/26/23	0.192	0.185	96.4
QC-04	8/26/23	0.192	0.180	93.8
QC-05	8/26/23	1.73	1.70	98.3
QC-06	8/26/23	1.73	1.71	98.8
QC-07	8/26/23	5.20	5.36	103
QC-08	8/26/23	5.20	5.33	103

ND = Not Detected.

Sea Water:

ID	Analysis Date	Theoretical Brodifacoum Concentration (ng/mL)	Observed Brodifacoum Concentration (ng/mL)	% Recovery
QC-09	8/26/23	Control	ND	------
QC-10	8/26/23	Control	ND	------
QC-11	8/26/23	0.192	0.135*	70.3
QC-12	8/26/23	0.192	0.137*	71.4
QC-17	8/29/23	0.192	0.166	86.5
QC-18	8/29/23	0.192	0.170	86.5
QC-13	8/26/23	1.73	1.67	96.5
QC-14	8/26/23	1.73	1.67	96.5
QC-15	8/26/23	5.20	5.11	98.3
QC-16	8/26/23	5.20	5.15	99.0

ND = Not Detected.

*-Due to a surrogate spiking error, this sample could not be accurately quantified. This result will be disregarded.
