# Supplementary material for: Anticoagulant residues associated with an attempted rodent eradication from a subtropical coral atoll
Source: PLoS One. 2026 Mar 23;21(3):e0344972. doi: 10.1371/journal.pone.0344972 (PMC13008109; doi:10.1371/journal.pone.0344972)
Supplement: S1 Appendix — (ZIP) [file pone.0344972.s001.zip › Supporting Information S1/23-034 Post 4 Brodifacoum Midway Island Avian Livers Report.pdf]

Wildlife Services<br>**NWRC**<br>National Wildlife Research Center<br>Analytical Services Report	United States Department of Agriculture<br>Animal Plant Health Inspection Service<br>Wildlife Services<br>National Wildlife Research Center<br>Laboratory Support Services Unit	Invoice #: 23-034/8<br>Date: 10/27/23<br>Page: 1 of 4

To: Carmen Antaky
Biologist
NWRC Hawai'i Field Station

Subject: Determination of brodifacoum in avian livers matrices from Midway Island (QA-3404)

Methods: 188A "Determination of Multiple Rodenticide Residues in Avian Liver by dSPE and LC-MS/MS" -Non-GLP

Analysis Dates: 10/10/23

Notebook References: AC169, pp.18-20, 28, 31
QC35, p.68

Analyst: Ben Abbo

Sample Description:

Seven avian liver and one bird carcass samples were submitted on 09/28/23. See sample descriptions on p.3.

Additional Comments:

- S230928-84 was necropsied and the liver removed for sampling.
- Three replicates of each sample were analyzed except for samples S230928-85, -86, and -87. There was insufficient sample to analyze multiple replicates for these samples. A single sample was analyzed for each of these. The mean, standard deviation, and coefficient of variance are reported.
- Control quail liver (S221018-03) was used as the matrix for QC samples.

Method Limit of Detection/Quantitation (MLOD/MLOQ) Values:

Method detection and quantitation limits were determined from by comparing the noise at the analyte retention in three unfortified control quail liver samples to the peak height of brodifacoum in three control quail liver samples fortified to ~15 ng/g brodifacoum. The detection limit was determined to be 3X the noise and the quantitation limit was determined to be 10X the noise found in the unfortified samples.

Method Limit of Detection (MLOD)

Matrix	Detection Limit
Avian Liver	1.3 ng/g

Method Limit of Quantitation (MLOQ)

Matrix	Quantitation Limit
Avian Liver	4.26 ng/g

Results:

Sample ID	Sample Description	Brodifacoum Conc (ng/g)	Descriptive Statistics	
S230928-83-A	Avian Liver, A-I-Post4-Se, Fuel Pier,	ND	$Mean_3$=	ND
S230928-83-B	Seabird, RTRP chick liver , 9/11/2023	ND	sd=	-----
S230928-83-C		ND	cv=	-----
S230928-84-A	Avian Liver, A-III-Post4Se, Old Runway,	ND	$Mean_3$=	ND
S230928-84-B	Seabird, White Tern (full carcass),	ND	sd=	-----
S230928-84-C	9/13/2023	ND	cv=	-----
S230928-85-A	Avian Liver, A-II-Post4-Sh, Harbor,	1280	$Mean_3$=	1280
S230928-85-B	Shorebird, PAGP 23-005 liver, 9/14/2023	INS	sd=	-----
S230928-85-C		INS	cv=	-----
S230928-86-A	Avian Liver, A-I-Post4-Du, Eastern	1.9*	$Mean_3$=	1.9*
S230928-86-B	Monument Seagrape, Duck, LADU liver 23-	INS	sd=	-----
S230928-86-C	722, 9/3/2023	INS	cv=	-----
S230928-87-A	Avian Liver, A-II-Post4-Du, Eastern	36.8	$Mean_3$=	36.8
S230928-87-B	Monument Seagrape, Duck, LADU liver 23-	INS	sd=	-----
S230928-87-C	695, 8/29/2023	INS	cv=	-----
S230928-88-A	Avian Liver, A-I-Post4-Myna, Cart road	970	$Mean_3$=	961
S230928-88-B	rockpiles, Passerines, COMY liver,	961	sd=	9.5
S230928-88-C	9/12/2023	951	cv=	0.99%
S230928-89-A	Avian Liver, A-II-Con-RUTU, North	118	$Mean_3$=	117
S230928-89-B	Beach, Shorebird, RUTU liver, 9/5/2023	118	sd=	1.7
S230928-89-C		115	cv=	1.5%
S230928-90-A	Avian Liver, A-I-Con-BTCU, Old Fuel	101	$Mean_3$=	102
S230928-90-B	Farm , Shorebird, BTCU liver, 9/4/2023	101	sd=	1.7
S230928-90-C		104	cv=	1.7%

ND = Not Detected.
*-Sample was below quantitation limit of 4.26 ng/g; result should be considered qualitative.
INS-Insufficient Sample

QC Results:

ID	Theoretical Brodifacoum Concentration (ng/g)	Observed Brodifacoum Concentration (ng/g)	% Recovery	Descriptive Statistics	
QC-73	Control	ND	N/A		
QC-74	Control	ND	N/A		
QC-75	Control	ND	N/A		
QC-76	14.0	13.5	96.4	$Mean_3$=	95.5%
QC-77	13.0	12.2	93.8	sd=	1.5%
QC-78	14.0	13.5	96.4	cv=	1.6%
QC-79	485	483	99.6	$Mean_3$=	100%
QC-80	484	494	102	sd=	1.4%
QC-81	458	456	99.6	cv=	1.4%
QC-82	2530	2540	100	$Mean_3$=	101%
QC-83	2270	2310	102	sd=	1.3%
QC-84	2060	2050	99.5	cv=	1.3%

ND = Not Detected.
