# Supplementary material for: Anticoagulant residues associated with an attempted rodent eradication from a subtropical coral atoll
Source: PLoS One. 2026 Mar 23;21(3):e0344972. doi: 10.1371/journal.pone.0344972 (PMC13008109; doi:10.1371/journal.pone.0344972)
Supplement: S1 Appendix — (ZIP) [file pone.0344972.s001.zip › Supporting Information S1/23-034 Post 4 Brodifacoum Midway Island Crustaceans Report.pdf]

To: Carmen Antaky
Biologist
NWRC Hawai'i Field Station

Subject: Determination of brodifacoum in crustaceans matrices from Midway Island (QA-3404)

Methods: 188A "Determination of Multiple Rodenticide Residues in Avian Liver by dSPE and LC-MS/MS" -Non-GLP

Analysis Dates: 10/05/23

Notebook References: AC169, pp.16, 19-20, 22, 29
QC35, p.68

Analyst: Ben Abbo

Sample Description:

Six crustacean samples were submitted on 09/28/23. See sample descriptions on p.3.

Additional Comments:

- Three replicates of each sample were analyzed. The mean, standard deviation, and coefficient of variance are reported.
- Control ghost crab (S220801-42) was used as the matrix for QC samples.

Method Limit of Detection/Quantitation (MLOD/MLOQ) Values:

Method detection and quantitation limits were determined from by comparing the noise at the analyte retention in three unfortified control ghost crab samples to the peak height of brodifacoum in three control ghost crab samples fortified to ~15 ng/g brodifacoum. The detection limit was determined to be 3X the noise and the quantitation limit was determined to be 10X the noise found in the unfortified samples.

Method Limit of Detection (MLOD)

Matrix	Detection Limit
Invertebrates	0.94 ng/g

Method Limit of Quantitation (MLOQ)

Matrix	Quantitation Limit
Invertebrates	3.12 ng/g

Results:

Sample ID	Sample Description	Brodifacoum Conc (ng/g)	Descriptive Statistics	
S230928-25-A	Crustaceans, A-I-Post4-Gh, CargoPier,	16.4	$Mean_3$=	15.1
S230928-25-B	Crabs, Crabs, Ghostcrabs, 9/12/2023	14.8	sd=	1.1
S230928-25-C		14.2	cv=	7.3%
S230928-26-A	Crustaceans, A-II-Post4-Gh, CargoPier,	11.4	$Mean_3$=	11.1
S230928-26-B	Crabs, Crabs, Ghostcrabs, 9/12/2023	11.0	sd=	0.23
S230928-26-C		11.0	cv=	2.1%
S230928-27-A	Crustaceans, B-I-Post4-Gh, Hale Honu,	8.84	$Mean_3$=	9.49
S230928-27-B	Crabs, Crabs, Ghostcrabs, 9/12/2023	10.2	sd=	0.68
S230928-27-C		9.43	cv=	7.2%
S230928-28-A	Crustaceans, B-II-Post4-Gh, Hale Honu,	6.62	$Mean_3$=	6.06
S230928-28-B	Crabs, Crabs, Ghostcrabs, 9/12/2023	5.79	sd=	0.49
S230928-28-C		5.76	cv=	8.1%
S230928-29-A	Crustaceans, C-I-Post4-Gh, RustyBucket,	7.85	$Mean_3$=	7.14
S230928-29-B	Crabs, Crabs, Ghostcrabs, 9/12/2023	6.27	sd=	0.80
S230928-29-C		7.30	cv=	11%
S230928-30-A	Crustaceans, C-II-Post4-Gh, RustyBucket,	8.08	$Mean_3$=	8.80
S230928-30-B	Crabs, Crabs, Ghostcrabs, 9/12/2023	9.53	sd=	0.73
S230928-30-C		8.78	cv=	8.3%

ND = Not Detected.

QC Results:

ID	Theoretical Brodifacoum Concentration (ng/g)	Observed Brodifacoum Concentration (ng/g)	% Recovery	Descriptive Statistics	
QC-13	Control	ND	N/A		
QC-14	Control	ND	N/A		
QC-15	Control	ND	N/A		
QC-16	13.4	14.2	106	$Mean_3$=	105%
QC-17	14.9	15.8	106	sd=	1.7%
QC-18	14.1	14.5	103	cv=	1.6%
QC-19	484	495	102	$Mean_3$=	101%
QC-20	477	484	102	sd=	1.4%
QC-21	469	467	99.6	cv=	1.4%
QC-22	2000	2040	102	$Mean_3$=	105%
QC-23	2340	2620	112	sd=	5.8%
QC-24	2450	2490	102	cv=	5.5%

ND = Not Detected.
