# Supplementary material for: Anticoagulant residues associated with an attempted rodent eradication from a subtropical coral atoll
Source: PLoS One. 2026 Mar 23;21(3):e0344972. doi: 10.1371/journal.pone.0344972 (PMC13008109; doi:10.1371/journal.pone.0344972)
Supplement: S1 Appendix — (ZIP) [file pone.0344972.s001.zip › Supporting Information S1/23-034 Post 4 Brodifacoum Midway Island Fish Report.pdf]

Wildlife Services NWRC National Wildlife Research Center<br>Analytical Services Report	United States Department of Agriculture<br>Animal Plant Health Inspection Service<br>Wildlife Services<br>National Wildlife Research Center<br>Laboratory Support Services Unit	Invoice #: 23-034/6<br>Date: 10/27/23<br>Page: 1 of 5

To: Carmen Antaky
Biologist
NWRC Hawai'i Field Station

Subject: Determination of brodifacoum in fish matrices from Midway Island (QA-3404)

Methods: 188A "Determination of Multiple Rodenticide Residues in Avian Liver by dSPE and LC-MS/MS" -Non-GLP

Analysis Dates: 10/10/23

Notebook References: AC169, pp.17, 20-21, 26, 31
QC35, p.68

Analyst: Ben Abbo

Sample Description:

Nineteen fish samples were submitted on 09/28/23. See sample descriptions on pp.3-4.

Additional Comments:

- Sample S230928-59 was lost during the homogenization process when its canister ruptured, causing the loss of the sample.
- Three replicates of each sample were analyzed. The mean, standard deviation, and coefficient of variance are reported.
- Control sea bass (S220906-01) was used as the matrix for QC samples.

Method Limit of Detection/Quantitation (MLOD/MLOQ) Values:

Method detection and quantitation limits were determined from by comparing the noise at the analyte retention in three unfortified control sea bass samples to the peak height of brodifacoum in three control sea bass samples fortified to ~15 ng/g brodifacoum. The detection limit was determined to be 3X the noise and the quantitation limit was determined to be 10X the noise found in the unfortified samples.

Method Limit of Detection (MLOD)

Matrix	Detection Limit
Fish	1.2 ng/g

Method Limit of Quantitation (MLOQ)

Matrix	Quantitation Limit
Fish	4.06 ng/g

Results:

Sample ID	Sample Description	Brodifacoum Conc (ng/g)	Descriptive Statistics	
S230928-58-A	Fish, A-I-Post4-M, R2, Mosquito Fish ,	20.1	$Mean_3$=	20.3
S230928-58-B	Gambusia, 9/7/2023	20.3	sd=	0.20
S230928-58-C		20.5	cv=	0.99%
S230928-59	Fish, A-II-Post4-M, R2, Mosquito Fish , Gambusia, 9/7/2023	Sample lost during processing		
S230928-60-A	Fish, B-I-Post4-M, Catchment, Mosquito	4.50	$Mean_3$=	4.76
S230928-60-B	Fish , Gambusia, 9/7/2023	4.45	sd=	0.49
S230928-60-C		5.33	cv=	10%
S230928-61-A	Fish, B-II-Post4-M, Catchment, Mosquito	16.1	$Mean_3$=	16.0
S230928-61-B	Fish , Gambusia, 9/7/2023	15.8	sd=	0.17
S230928-61-C		16.1	cv=	1.1%
S230928-62-A	Fish, C-I-Post4-M, Drainage Pond,	90.7	$Mean_3$=	91.2
S230928-62-B	Mosquito Fish , Gambusia, 9/7/2023	92.2	sd=	0.90
S230928-62-C		90.6	cv=	0.99%
S230928-63-A	Fish, C-II-Post4-M, Drainage Pond,	82.5	$Mean_3$=	83.2
S230928-63-B	Mosquito Fish , Gambusia, 9/7/2023	83.4	sd=	0.62
S230928-63-C		83.7	cv=	0.75%
S230928-64-A	Fish, A-I-Post4-Bait, Cargo Pier, Bait Fish,	2.0*	$Mean_3$=	1.6*
S230928-64-B	Flagtail, 9/6/2023	1.6*	sd=	0.35
S230928-64-C		1.3*	cv=	22%
S230928-65-A	Fish, A-II-Post4-Bait, Cargo Pier, Bait Fish,	34.3	$Mean_3$=	34.1
S230928-65-B	Flagtail, 9/6/2023	33.2	sd=	0.86
S230928-65-C		34.9	cv=	2.5%
S230928-66-A	Fish, B-I-Post4-Bait, Hale Honu, Bait Fish,	22.3	$Mean_3$=	22.4
S230928-66-B	Flagtail, 9/6/2023	23.1	sd=	0.61
S230928-66-C		21.9	cv=	2.7%
S230928-67-A	Fish, B-II-Post4-Bait, Hale Honu, Bait Fish,	ND	$Mean_3$=	ND
S230928-67-B	Flagtail, 9/6/2023	ND	sd=	-----
S230928-67-C		ND	cv=	-----
S230928-68-A	Fish, C-I-Post4-Bait, RustyBucket, Bait	22.1	$Mean_3$=	22.4
S230928-68-B	Fish, Flagtail, 9/6/2023	24.4	sd=	1.9
S230928-68-C		20.6	cv=	8.5%
S230928-69-A	Fish, C-II-Post4-Bait, RustyBucket, Bait	17.5	$Mean_3$=	17.1
S230928-69-B	Fish, Flagtail, 9/6/2023	16.4	sd=	0.64
S230928-69-C		17.5	cv=	3.7%

ND = Not Detected.
*-Value is below the method quantitation limit of 4.06 ng/g

Results:

Sample ID	Sample Description	Brodifacoum Conc (ng/g)	Descriptive Statistics	
S230928-70-A	Fish, A-I-Post4-Reef, CargoPier, Reef Fish,	3.5*	$Mean_3$=	1.8*
S230928-70-B	Moi, 9/6/2023	ND	sd=	1.5
S230928-70-C		1.3*	cv=	83%
S230928-71-A	Fish, A-II-Post4-Reef, CargoPier, Reef Fish,	ND	$Mean_3$=	ND
S230928-71-B	Moi, 9/6/2023	ND	sd=	-----
S230928-71-C		ND	cv=	-----
S230928-72-A	Fish, B-I-Post4-Reef, Hale Honu, Reef Fish,	ND	$Mean_3$=	ND
S230928-72-B	Goatfish, 9/6/2023	ND	sd=	-----
S230928-72-C		ND	cv=	-----
S230928-73-A	Fish, B-II-Post4-Reef, Hale Honu, Reef	ND	$Mean_3$=	ND
S230928-73-B	Fish, Goatfish, 9/6/2023	ND	sd=	-----
S230928-73-C		ND	cv=	-----
S230928-74-A	Fish, C-I-Post4-Reef, RustyBucket, Reef	ND	$Mean_3$=	ND
S230928-74-B	Fish, Goatfish, 9/6/2023	ND	sd=	-----
S230928-74-C		ND	cv=	-----
S230928-75-A	Fish, C-II-Post4-Reef, RustyBucket, Reef	ND	$Mean_3$=	ND
S230928-75-B	Fish, Goatfish, 9/6/2023	ND	sd=	-----
S230928-75-C		ND	cv=	-----
S230928-76-A	Fish, C-I-Post4-ReefFishv2, RustyBucket,	ND	$Mean_3$=	ND
S230928-76-B	Reef Fish, Taape, 9/15/2023	ND	sd=	-----
S230928-76-C		ND	cv=	-----

ND = Not Detected.
*-Value is below the method quantitation limit of 4.06 ng/g

QC Results:

ID	Theoretical Brodifacoum Concentration (ng/g)	Observed Brodifacoum Concentration (ng/g)	% Recovery	Descriptive Statistics	
QC-49	Control	ND	N/A		
QC-50	Control	ND	N/A		
QC-51	Control	ND	N/A		
QC-52	13.4	13.2	98.5	$Mean_3$=	102%
QC-53	12.8	13.5	106	sd=	3.8%
QC-54	13.6	13.6	102	cv=	3.7%
QC-55	470	480	102	$Mean_3$=	103%
QC-56	442	455	103	sd=	0.58%
QC-57	466	481	103	cv=	0.56%
QC-58	2170	2190	101	$Mean_3$=	101%
QC-59	2420	2450	101	sd=	0.0%
QC-60	2260	2280	101	cv=	0.0%

ND = Not Detected.
