# Supplementary material for: Anticoagulant residues associated with an attempted rodent eradication from a subtropical coral atoll
Source: PLoS One. 2026 Mar 23;21(3):e0344972. doi: 10.1371/journal.pone.0344972 (PMC13008109; doi:10.1371/journal.pone.0344972)
Supplement: S1 Appendix — (ZIP) [file pone.0344972.s001.zip › Supporting Information S1/23-034 Post 4 Brodifacoum Midway Island Invertebrates Report.pdf]

To: Carmen Antaky
Biologist
NWRC Hawai'i Field Station

Subject: Determination of brodifacoum in invertebrate matrices from Midway Island (QA-3404)

Methods: 188A "Determination of Multiple Rodenticide Residues in Avian Liver by dSPE and LC-MS/MS" -Non-GLP

Analysis Dates: 10/04/23

Notebook References: AC169, pp.15, 19-21, 23
QC35, p.68

Analyst: Ben Abbo

Sample Description:

Twenty-four invertebrate samples were submitted on 09/28/23. See sample descriptions on pp.3-4.

Additional Comments:

- Three replicates of each sample were analyzed. The mean, standard deviation, and coefficient of variance are reported.
- Control crickets (S221018-01) were used as the matrix for QC samples.

Method Limit of Detection/Quantitation (MLOD/MLOQ) Values:

Method detection and quantitation limits were determined from by comparing the noise at the analyte retention in three unfortified control cricket samples to the peak height of brodifacoum in three control cricket samples fortified to ~15 ng/g brodifacoum. The detection limit was determined to be 3X the noise and the quantitation limit was determined to be 10X the noise found in the unfortified samples.

Method Limit of Detection (MLOD)

Matrix	Detection Limit
Invertebrates	1.2 ng/g

Method Limit of Quantitation (MLOQ)

Matrix	Quantitation Limit
Invertebrates	3.97 ng/g

Results:

Sample ID	Sample Description	Brodifacoum Conc (ng/g)	Descriptive Statistics	
S230928-01-A	Invertebrates, A-I-Post4-G, Radar, Grubs,	6.26	Mean3=	5.57
S230928-01-B	Grubs, Emerald beetle, 9/13/2023	4.78	sd=	0.75
S230928-01-C		5.68	cv=	14%
S230928-02-A	Invertebrates, A-II-Post4-G, Radar, Grubs,	5.90	Mean3=	5.91
S230928-02-B	Grubs, Emerald beetle, 9/13/2023	5.65	sd=	0.27
S230928-02-C		6.18	cv=	4.6%
S230928-03-A	Invertebrates, B-I-Post4-G, Brackish, Grubs,	10.5	Mean3=	7.87
S230928-03-B	Grubs, Emerald beetle, 9/13/2023	6.82	sd=	2.3
S230928-03-C		6.28	cv=	29%
S230928-04-A	Invertebrates, B-II-Post4-G, Brackish,	7.24	Mean3=	7.07
S230928-04-B	Grubs, Grubs, Emerald beetle, 9/13/2023	7.06	sd=	0.17
S230928-04-C		6.90	cv=	2.4%
S230928-05-A	Invertebrates, C-I-Post4-G, RustyBucket,	ND	Mean3=	ND
S230928-05-B	Grubs, Grubs, Emerald beetle, 9/13/2023	ND	sd=	-----
S230928-05-C		ND	cv=	-----
S230928-06-A	Invertebrates, C-II-Post4-G, RustyBucket,	1.8*	Mean3=	1.7*
S230928-06-B	Grubs, Grubs, Emerald beetle, 9/13/2023	1.8*	sd=	0.23
S230928-06-C		1.4*	cv=	14%
S230928-07-A	Invertebrates, A-I-Post4-R, Radar, Roaches,	14.1	Mean3=	9.94
S230928-07-B	Roaches, Blattodea, 9/18/2023	6.14	sd=	4.0
S230928-07-C		9.58	cv=	40%
S230928-08-A	Invertebrates, A-II-Post4-R, Radar, Roaches,	2.2*	Mean3=	2.2*
S230928-08-B	Roaches, Blattodea, 9/18/2023	2.1*	sd=	0.058
S230928-08-C		2.2*	cv=	2.6%
S230928-09-A	Invertebrates, B-I-Post4-R, Brackish,	11.2	Mean3=	11.9
S230928-09-B	Roaches, Roaches, Blattodea, 9/18/2023	8.85	sd=	3.5
S230928-09-C		15.7	cv=	29%
S230928-10-A	Invertebrates, B-II-Post4-R, Brackish,	12.4	Mean3=	12.9
S230928-10-B	Roaches, Roaches, Blattodea, 9/18/2023	14.0	sd=	0.92
S230928-10-C		12.4	cv=	7.1%
S230928-11-A	Invertebrates, C-I-Post4-R, RustyBucket,	29.0	Mean3=	25.2
S230928-11-B	Roaches, Roaches, Blattodea, 9/18/2023	22.0	sd=	3.5
S230928-11-C		24.6	cv=	14%
S230928-12-A	Invertebrates, C-II-Post4-R, RustyBucket,	7.45	Mean3=	7.79
S230928-12-B	Roaches, Roaches, Blattodea, 9/18/2023	8.39	sd=	0.52
S230928-12-C		7.53	cv=	6.7%

ND = Not Detected.

*-Value is below the method quantitation limit of 4.69 ng/g

Results:

Sample ID	Sample Description	Brodifacoum Conc (ng/g)	Descriptive Statistics	
S230928-13-A	Invertebrates, D-I-Post4-R, Ballfield,	3.6*	Mean3=	3.4*
S230928-13-B	Roaches, Roaches, Blattodea, 9/18/2023	3.3*	sd=	0.17
S230928-13-C		3.3*	cv=	5.0%
S230928-14-A	Invertebrates, D-II-Post4-R, Ballfield,	6.36	Mean3=	6.02
S230928-14-B	Roaches, Roaches, Blattodea, 9/18/2023	6.12	sd=	0.39
S230928-14-C		5.59	cv=	6.5%
S230928-15-A	Invertebrates, E-I-Post4-R, Parade Ground,	5.87	Mean3=	5.89
S230928-15-B	Roaches, Roaches, Blattodea, 9/18/2023	6.40	sd=	0.50
S230928-15-C		5.41	cv=	8.5%
S230928-16-A	Invertebrates, E-II-Post4-R, Parade Ground,	4.91	Mean3=	4.68
S230928-16-B	Roaches, Roaches, Blattodea, 9/18/2023	4.72	sd=	0.25
S230928-16-C		4.42	cv=	5.3%
S230928-17-A	Invertebrates, F-I-Post4-R, SE Corner Radar	4.66	Mean3=	4.45
S230928-17-B	Hill, Roaches, Roaches, Blattodea,	4.28	sd=	0.19
S230928-17-C	9/18/2023	4.42	cv=	4.3%
S230928-18-A	Invertebrates, F-II-Post4-R, SE Corner	8.01	Mean3=	8.00
S230928-18-B	Radar Hill, Roaches, Roaches, Blattodea,	7.21	sd=	0.78
S230928-18-C	9/18/2023	8.77	cv=	9.8%
S230928-19-A	Invertebrates, A-I-Post4-Pitfall, Radar,	4.64	Mean3=	4.14
S230928-19-B	Pitfall, Pitfall, Pooled, 9/13/2023	2.9*	sd=	1.1
S230928-19-C		4.88	cv=	27%
S230928-20-A	Invertebrates, A-II-Post4-Pitfall, Radar,	3.2*	Mean3=	3.9*
S230928-20-B	Pitfall, Pitfall, Pooled, 9/13/2023	4.75	sd=	0.78
S230928-20-C		3.8*	cv=	20%
S230928-21-A	Invertebrates, B-I-Post4-Pitfall, Brackish,	17.7	Mean3=	17.0
S230928-21-B	Pitfall, Pitfall, Pooled, 9/13/2023	14.9	sd=	1.8
S230928-21-C		18.3	cv=	11%
S230928-22-A	Invertebrates, B-II-Post4-Pitfall, Brackish,	9.11	Mean3=	12.4
S230928-22-B	Pitfall, Pitfall, Pooled, 9/13/2023	16.00	sd=	3.5
S230928-22-C		12.00	cv=	28%
S230928-23-A	Invertebrates, C-I-Post4-Pitfall,	7.13	Mean3=	5.96
S230928-23-B	RustyBucket, Pitfall, Pitfall, Pooled,	6.20	sd=	1.3
S230928-23-C	9/13/2023	4.55	cv=	22%
S230928-24-A	Invertebrates, C-II-Post4-Pitfall,	4.0*	Mean3=	3.9*
S230928-24-B	RustyBucket, Pitfall, Pitfall, Pooled,	3.0*	sd=	0.85
S230928-24-C	9/13/2023	4.68	cv=	22%

ND = Not Detected.

*-Value is below the method quantitation limit of 3.97 ng/g

QC Results:

ID	Theoretical Brodifacoum Concentration (ng/g)	Observed Brodifacoum Concentration (ng/g)	% Recovery	Descriptive Statistics	
QC-01	Control	ND	N/A		
QC-02	Control	ND	N/A		
QC-03	Control	ND	N/A		
QC-04	14.6	13.7	93.8	$Mean_3$=	95.9%
QC-05	14.4	14.2	98.6	sd=	2.5%
QC-06	12.5	11.9	95.2	cv=	2.6%
QC-07	416	399	95.9	$Mean_3$=	96.3%
QC-08	561	543	96.8	sd=	0.46%
QC-09	503	484	96.2	cv=	0.48%
QC-10	2010	1960	97.5	$Mean_3$=	98.0%
QC-11	2050	2020	98.5	sd=	0.50%
QC-12	2100	2060	98.1	cv=	0.51%

ND = Not Detected.
