# Supplementary material for: Anticoagulant residues associated with an attempted rodent eradication from a subtropical coral atoll
Source: PLoS One. 2026 Mar 23;21(3):e0344972. doi: 10.1371/journal.pone.0344972 (PMC13008109; doi:10.1371/journal.pone.0344972)
Supplement: S1 Appendix — (ZIP) [file pone.0344972.s001.zip › Supporting Information S1/23-034 Post 4 Brodifacoum Midway Island Plants Report.pdf]

Wildlife Services **NWRC** National Wildlife Research Center<br>Analytical Services Report	United States Department of Agriculture<br>Animal Plant Health Inspection Service<br>Wildlife Services<br>National Wildlife Research Center<br>Laboratory Support Services Unit	Invoice #: 23-034/4<br>Date: 10/27/23<br>Page: 1 of 3

To: Carmen Antaky
Biologist
NWRC Hawai'i Field Station

Subject: Determination of brodifacoum in plant matrices from Midway Island (QA-3404)

Methods: 188A "Determination of Multiple Rodenticide Residues in Avian Liver by dSPE and LC-MS/MS" -Non-GLP

Analysis Dates: 10/07/23

Notebook References: AC169, pp.16, 19-20, 25, 30
QC35, p.68

Analyst: Ben Abbo

Sample Description:

Six plant samples were submitted on 09/28/23. See sample descriptions on p.3.

Additional Comments:

- Three replicates of each sample were analyzed. The mean, standard deviation, and coefficient of variance are reported.
- Samples were extracted on 10/06/23, but an error with the instrument caused their analysis to be delayed to 10/07/23. The QC sample recoveries were normal, indicating that the analysis was not impacted by the delay.
- Control lime (S221013-01) was used as the matrix for QC samples.

Method Limit of Detection/Quantitation (MLOD/MLOQ) Values:

Method detection and quantitation limits were determined from by comparing the noise at the analyte retention in three unfortified control lime samples to the peak height of brodifacoum in three control lime samples fortified to ~15 ng/g brodifacoum. The detection limit was determined to be 3X the noise and the quantitation limit was determined to be 10X the noise found in the unfortified samples.

Method Limit of Detection (MLOD)

Matrix	Detection Limit
Plants	1.1 ng/g

Method Limit of Quantitation (MLOQ)

Matrix	Quantitation Limit
Plants	3.72 ng/g

Results:

Sample ID	Sample Description	Brodifacoum Conc (ng/g)	Descriptive Statistics	
S230928-38-A	Plants, A-I-Post4-Pl, Community Garden A,	ND	$Mean_3$=	ND
S230928-38-B	Fruit, Fruit, Kaffir lime leaves, 9/11/2023	ND	sd=	-----
S230928-38-C		ND	cv=	-----
S230928-39-A	Plants, A-II-Post4-Pl, Community Garden A,	ND	$Mean_3$=	ND
S230928-39-B	Fruit, Fruit, Kaffir lime leaves, 9/11/2023	ND	sd=	-----
S230928-39-C		ND	cv=	-----
S230928-40-A	Plants, B-I-Post4-Pl, Orchard, Fruit, Fruit,	ND	$Mean_3$=	ND
S230928-40-B	Persian lime, 9/11/2023	ND	sd=	-----
S230928-40-C		ND	cv=	-----
S230928-41-A	Plants, B-II-Post4-Pl, Orchard, Fruit, Fruit,	ND	$Mean_3$=	ND
S230928-41-B	Persian lime, 9/11/2023	ND	sd=	-----
S230928-41-C		ND	cv=	-----
S230928-42-A	Plants, C-I-Post4-Pl, Community Garden B,	ND	$Mean_3$=	ND
S230928-42-B	Fruit, Fruit, Papaya, 9/11/2023	ND	sd=	-----
S230928-42-C		ND	cv=	-----
S230928-43-A	Plants, C-II-Post4-Pl, Community Garden B,	ND	$Mean_3$=	ND
S230928-43-B	Fruit, Fruit, Papaya, 9/11/2023	ND	sd=	-----
S230928-43-C		ND	cv=	-----

ND = Not Detected.

QC Results:

ID	Theoretical Brodifacoum Concentration (ng/g)	Observed Brodifacoum Concentration (ng/g)	% Recovery	Descriptive Statistics	
QC-37	Control	ND	N/A		
QC-38	Control	ND	N/A		
QC-39	Control	ND	N/A		
QC-40	13.2	13.4	102	$Mean_3$=	101%
QC-41	12.5	12.8	102	sd=	2.4%
QC-42	13.7	13.4	97.8	cv=	2.4%
QC-43	470	490	104	$Mean_3$=	103%
QC-44	556	580	104	sd=	1.2%
QC-45	460	471	102	cv=	1.2%
QC-46	2270	2330	103	$Mean_3$=	103%
QC-47	2020	2090	104	sd=	1.0%
QC-48	2400	2450	102	cv=	0.97%

ND = Not Detected.
