# Supplementary material for: Anticoagulant residues associated with an attempted rodent eradication from a subtropical coral atoll
Source: PLoS One. 2026 Mar 23;21(3):e0344972. doi: 10.1371/journal.pone.0344972 (PMC13008109; doi:10.1371/journal.pone.0344972)
Supplement: S1 Appendix — (ZIP) [file pone.0344972.s001.zip › Supporting Information S1/23-034 Post 4 Brodifacoum Midway Island Reptiles Report.pdf]

To: Carmen Antaky
Biologist
NWRC Hawai'i Field Station

Subject: Determination of brodifacoum in reptile matrices from Midway Island (QA-3404)

Methods: 188A "Determination of Multiple Rodenticide Residues in Avian Liver by dSPE and LC-MS/MS" -Non-GLP

Analysis Dates: 10/07/23

Notebook References: AC169, pp.16, 19-20, 22, 29
QC35, p.68

Analyst: Ben Abbo

Sample Description:

Six reptile samples were submitted on 09/28/23. See sample descriptions on p.3.

Additional Comments:

- Three replicates of each sample were analyzed. The mean, standard deviation, and coefficient of variance are reported.
- Samples were extracted on 10/06/23, but an error with the instrument caused their analysis to be delayed to 10/07/23. The QC sample recoveries were normal, indicating that the analysis was not impacted by the delay
- Control gecko (S220801-47) was used as the matrix for QC samples.

Method Limit of Detection/Quantitation (MLOD/MLOQ) Values:

Method detection and quantitation limits were determined from by comparing the noise at the analyte retention in three unfortified control gecko samples to the peak height of brodifacoum in three control gecko samples fortified to ~15 ng/g brodifacoum. The detection limit was determined to be 3X the noise and the quantitation limit was determined to be 10X the noise found in the unfortified samples.

Method Limit of Detection (MLOD)

Matrix	Detection Limit
Reptile	2.0 ng/g

Method Limit of Quantitation (MLOQ)

Matrix	Quantitation Limit
Reptile	6.59 ng/g

Results:

Sample ID	Sample Description	Brodifacoum Conc (ng/g)	Descriptive Statistics	
S230928-77-A	Reptiles, A-I-Post4-Ge, Clipper, Geckos,	14.2	$Mean_3$=	14.3
S230928-77-B	House gecko, 9/11/2023	14.0	sd=	0.31
S230928-77-C		14.6	cv=	2.2%
S230928-78-A	Reptiles, A-II-Post4-Ge, Clipper, Geckos,	13.6	$Mean_3$=	13.4
S230928-78-B	indo-pacific gecko, 9/11/2023	13.2	sd=	0.20
S230928-78-C		13.4	cv=	1.5%
S230928-79-A	Reptiles, B-I-Post4-Ge, Gym/Store, Geckos,	45.3	$Mean_3$=	45.5
S230928-79-B	indo-pacific gecko, 9/11/2023	45.1	sd=	0.53
S230928-79-C		46.1	cv=	1.2%
S230928-80-A	Reptiles, B-II-Post4-Ge, Gym/Store,	44.7	$Mean_3$=	47.4
S230928-80-B	Geckos, indo-pacific gecko, 9/11/2023	49.0	sd=	2.4
S230928-80-C		48.5	cv=	5.1%
S230928-81-A	Reptiles, C-I-Post4-Ge, FWS Office,	57.1	$Mean_3$=	57.1
S230928-81-B	Geckos, Mourning gecko, 9/11/2023	57.2	sd=	0.15
S230928-81-C		56.9	cv=	0.26%
S230928-82-A	Reptiles, C-II-Post4-Ge, FWS Office,	47.8	$Mean_3$=	46.5
S230928-82-B	Geckos, indo-pacific gecko, 9/11/2023	46.5	sd=	1.3
S230928-82-C		45.3	cv=	2.8%

ND = Not Detected.

QC Results:

ID	Theoretical Brodifacoum Concentration (ng/g)	Observed Brodifacoum Concentration (ng/g)	% Recovery	Descriptive Statistics	
QC-61	Control	ND	N/A		
QC-62	Control	ND	N/A		
QC-63	Control	ND	N/A		
QC-64	14.0	14.7	105	$Mean_3$=	101%
QC-65	13.2	13.1	99.2	sd=	3.5%
QC-66	15.5	15.3	98.7	cv=	3.5%
QC-67	480	489	102	$Mean_3$=	103%
QC-68	482	499	104	sd=	1.2%
QC-69	495	506	102	cv=	1.2%
QC-70	2180	2230	102	$Mean_3$=	101%
QC-71	2140	2170	101	sd=	0.58%
QC-72	2270	2290	101	cv=	0.57%

ND = Not Detected.
