# Supplementary material for: Anticoagulant residues associated with an attempted rodent eradication from a subtropical coral atoll
Source: PLoS One. 2026 Mar 23;21(3):e0344972. doi: 10.1371/journal.pone.0344972 (PMC13008109; doi:10.1371/journal.pone.0344972)
Supplement: S1 Appendix — (ZIP) [file pone.0344972.s001.zip › Supporting Information S1/23-034 Post 4 Brodifacoum Midway Island Soils Report.pdf]

To: Carmen Antaky
Biologist
NWRC Hawai'i Field Station

Subject: Determination of brodifacoum in soil matrices from Midway Island (QA-3404)

Methods: 188A "Determination of Multiple Rodenticide Residues in Avian Liver by dSPE and LC-MS/MS" -Non-GLP

Analysis Dates: 10/05/23

Notebook References: AC169, pp.16, 19-20, 24, 29
QC35, p.68

Analyst: Ben Abbo

Sample Description:

Seven soil samples were submitted on 09/28/23. See sample descriptions on p.3.

Additional Comments:

- Three replicates of each sample were analyzed. The mean, standard deviation, and coefficient of variance are reported.
- Replicate sample S230928-34-B had a brodifacoum concentration of 4.0 ng/g. Since the two other replicates for this sample had no detectable levels of brodifacoum, it was determined that this was likely a contamination during the extraction process and not a valid result. The value was disregarded and the sample reported as not detected.
- Control soil (S220801-61) was used as the matrix for QC samples.

Method Limit of Detection/Quantitation (MLOD/MLOQ) Values:

Method detection and quantitation limits were determined from by comparing the noise at the analyte retention in three unfortified control soil samples to the peak height of brodifacoum in three control soil samples fortified to ~15 ng/g brodifacoum. The detection limit was determined to be 3X the noise and the quantitation limit was determined to be 10X the noise found in the unfortified samples.

Method Limit of Detection (MLOD)

Matrix	Detection Limit
Soil	1.3 ng/g

Method Limit of Quantitation (MLOQ)

Matrix	Quantitation Limit
Soil	4.29 ng/g

Results:

Sample ID	Sample Description	Brodifacoum Conc (ng/g)	Descriptive Statistics	
S230928-31-A	Soil, A-I-Post4-S, Radar, Soil, 9/8/2023	ND	$Mean_3$=	ND
S230928-31-B		ND	sd=	-----
S230928-31-C		ND	cv=	-----
S230928-32-A	Soil, B-I-Post4-S, Brackish, Soil, 9/8/2023	ND	$Mean_3$=	ND
S230928-32-B		ND	sd=	-----
S230928-32-C		ND	cv=	-----
S230928-33-A	Soil, C-I-Post4-S, Community Garden, Soil, 9/11/2023	ND	$Mean_3$=	ND
S230928-33-B		ND	sd=	-----
S230928-33-C		ND	cv=	-----
S230928-34-A	Soil, A-I-Post4-SS, Parade Ground, Soil, 9/11/2023	ND	$Mean_3$=	ND
S230928-34-B		4.0+	sd=	-----
S230928-34-C		ND	cv=	-----
S230928-35-A	Soil, B-I-Post4-SS, Orchard, Soil, 9/11/2023	1.6*	$Mean_3$=	ND
S230928-35-B		ND	sd=	-----
S230928-35-C		ND	cv=	-----
S230928-36-A	Soil, C-I-Post4-SS, Community Garden, Soil, uncovered, 9/11/2023	ND	$Mean_3$=	ND
S230928-36-B		ND	sd=	-----
S230928-36-C		ND	cv=	-----
S230928-37-A	Soil, D-I-Post4-SS, Ballfield, Soil, 9/11/2023	4.45	$Mean_3$=	3.4*
S230928-37-B		3.1*	sd=	0.96
S230928-37-C		2.6*	cv=	28%

ND = Not Detected.
*-Sample was below quantitation limit of 4.29 ng/g; result should be considered qualitative.
+-Sample was determined to be a contaminant and was disregarded.

QC Results:

ID	Theoretical Brodifacoum Concentration (ng/g)	Observed Brodifacoum Concentration (ng/g)	% Recovery	Descriptive Statistics	
QC-25	Control	ND	N/A		
QC-26	Control	ND	N/A		
QC-27	Control	ND	N/A		
QC-28	13.0	12.1	93.1	$Mean_3$=	95.8%
QC-29	14.6	14.3	97.9	sd=	2.5%
QC-30	14.1	13.6	96.5	cv=	2.6%
QC-31	500	504	101	$Mean_3$=	101%
QC-32	487	487	100	sd=	0.58%
QC-33	481	485	101	cv=	0.57%
QC-34	2260	2300	102	$Mean_3$=	101%
QC-35	2150	2180	101	sd=	1.2%
QC-36	2280	2270	99.6	cv=	1.2%

ND = Not Detected.
