# Supplementary material for: Anticoagulant residues associated with an attempted rodent eradication from a subtropical coral atoll
Source: PLoS One. 2026 Mar 23;21(3):e0344972. doi: 10.1371/journal.pone.0344972 (PMC13008109; doi:10.1371/journal.pone.0344972)
Supplement: S1 Appendix — (ZIP) [file pone.0344972.s001.zip › Supporting Information S1/23-034 Post 4 Brodifacoum Midway Island Waters Report.pdf]

To: Carmen Antaky
Biologist
NWRC Hawai'i Field Station

Subject: Determination of brodifacoum in Waters from Midway Island (QA-3404)

Methods: 188A "Determination of Multiple Rodenticide Residues in Avian Liver by dSPE and LC-MS/MS" -Non-GLP
"Determination of Brodifacoum Residues in Water"-Non-GLP

Analysis Dates: 10/12/23

Notebook References: AC169, pp.20-21, 32-33
QC35, p.68

Analyst: Ben Abbo

Sample Description:

Fourteen samples of water from Midway Atoll were submitted on 09/28/23. See sample descriptions on p.3-4.

Additional Comments:

- Two replicates of each sample were analyzed.
- Cache la Poudre River water from Laporte, Colorado (S230825-01) was used as the matrix for QC samples for fresh water.
- Synthetic sea water (NWRC ID# 7262) was used as the matrix for QC samples for sea water.

Sample Preparation and Extraction:

Water Extraction Procedure:

1. Add 60 mL of seawater to a 125-mL separatory funnel.
2. Add 0.020 mL surrogate to all and 0.040 mL of acetonitrile or 75X brodifacoum stock as indicated.
3. Add 20 mL chloroform.
4. Add ~8.5g sodium chloride.
5. Add 10 mL 1M hydrochloric acid.
6. Cap and shake for 8-10s, let set 1 minute. Repeat 2X.
7. Dispense chloroform phase into 25-mL glass tube taking care to not transfer any water phase.
8. Remove solvent in a 60°C N-Evap with a gentle flow of nitrogen gas.
9. Add 0.300 mL acetonitrile, vortex thoroughly, wetting as much of the inside surface of the tube as possible.
10. Add 1.200 mL pH 9.5 20-mM ammonium acetate buffer and vortex thoroughly.
11. Transfer the sample to an autosampler vial and assay by LC-MS/MS.

Method Limit of Detection/Quantitation (MLOD/MLOQ) Values:

Method detection and quantitation limits were determined by comparing the noise at the analyte retention in two unfortified control water samples to the peak height of brodifacoum in two control water samples fortified to 0.0214 ng/mL brodifacoum. The detection limit was determined to be 3X the noise and the quantitation limit was determined to be 10X the noise found in the unfortified samples.

Method Limit of Detection (MLOD)

Matrix	Detection Limit
Fresh water	0.0071 ng/mL
Sea water	0.0014 ng/mL

Method Limit of Quantitation (MLOQ)

Matrix	Quantitation Limit
Fresh water	0.0236 ng/mL
Sea water	0.00453 ng/mL

Results:

Fresh Water

Sample ID	Sample Description	Brodifacoum (ng/g)	Descriptive Statistics	
S230928-44-A	Water, A-I-Post4-DW, FWS Office,	ND	Avg_2=	ND
S230928-44-B	Drinking water, 9/8/2023	ND	sd=	------
			cv=	------
S230928-45-A	Water, B-I-Post4-DW, Charlie Barracks,	ND	Avg_2=	ND
S230928-45-B	Drinking water, 9/8/2023	ND	sd=	------
			cv=	------
S230928-46-A	Water, A-I-Post4-WC, Catchment, Drainage	ND	Avg_2=	ND
S230928-46-B	Water, 9/7/2023	ND	sd=	------
			cv=	------
S230928-47-A	Water, B-I-Post4-WC, Bowling Alley,	ND	Avg_2=	ND
S230928-47-B	Drainage Water, 9/7/2023	ND	sd=	------
			cv=	------
S230928-48-A	Water, C-I-Post4-WC, Catchment, Drainage	ND	Avg_2=	ND
S230928-48-B	Water, 9/7/2023	ND	sd=	------
			cv=	------
S230928-53-A	Water, A-I-Post4-Fr, Radar, Fresh Water,	ND	Avg_2=	ND
S230928-53-B	9/8/2023	ND	sd=	------
			cv=	------
S230928-54-A	Water, B-I-Post4-Fr, Brackish, Fresh Water,	ND	Avg_2=	ND
S230928-54-B	9/8/2023	ND	sd=	------
			cv=	------
S230928-55-A	Water, C-I-Post4-Fr, R2, Fresh Water,	ND	Avg_2=	ND
S230928-55-B	9/8/2023	ND	sd=	------
			cv=	------
S230928-56-A	Water, D-I-Post4-Fr, Office Guzzer, Fresh	ND	Avg_2=	ND
S230928-56-B	Water, 9/8/2023	ND	sd=	------
			cv=	------
S230928-57-A	Water, E-I-Post4-Fr, Drainage Pond, Fresh	ND	Avg_2=	ND
S230928-57-B	Water, 9/7/2023	ND	sd=	------
			cv=	------

ND-Not Detected

Results:

Sea Water

**Sample ID**	**Sample Description**	**Brodifacoum (ng/g)**	Descriptive Statistics	
S230928-49-A	Water, A-I-Post4-Oc, CargoPier, Ocean	ND	Avg_2=	ND
S230928-49-B	Water, 9/6/2023	ND	sd=	------
			cv=	------
S230928-50-A	Water, B-I-Post4-Oc, Hale Honu, Ocean	ND	Avg_2=	ND
S230928-50-B	Water, 9/6/2023	ND	sd=	------
			cv=	------
S230928-51-A	Water, C-I-Post4-Oc, RustyBucket, Ocean	ND	Avg_2=	ND
S230928-51-B	Water, 9/6/2023	ND	sd=	------
			cv=	------
S230928-52-A	Water, D-I-Post4-Oc, Harbor, Ocean Water,	ND	Avg_2=	ND
S230928-52-B	9/6/2023	ND	sd=	------
			cv=	------

ND-Not Detected

QC Results:

Fresh Water:

ID	Theoretical Brodifacoum Concentration (ng/mL)	Observed Brodifacoum Concentration (ng/mL)	% Recovery
QC-85	Control	ND	-----
QC-86	Control	ND	-----
QC-87	0.0214	0.021*	98.1
QC-88	0.0214	0.022*	103
QC-89	1.73	1.71	98.8
QC-90	1.73	1.73	100
QC-91	5.20	5.09	97.9
QC-92	5.20	4.98	95.8

ND-Not Detected
*-Value is below the quantitation limit of 0.0236 ng/mL

Sea Water:

ID	Theoretical Brodifacoum Concentration (ng/mL)	Observed Brodifacoum Concentration (ng/mL)	% Recovery
QC-93	Control	ND	------
QC-94	Control	ND	------
QC-95	0.0214	0.0220	103
QC-96	0.0214	0.0225	105
QC-97	1.73	1.68	97.1
QC-98	1.73	1.67	96.5
QC-99	5.20	5.12	98.5
QC-100	5.20	5.09	97.9

ND-Not Detected
