# Supplementary material for: Anticoagulant residues associated with an attempted rodent eradication from a subtropical coral atoll
Source: PLoS One. 2026 Mar 23;21(3):e0344972. doi: 10.1371/journal.pone.0344972 (PMC13008109; doi:10.1371/journal.pone.0344972)
Supplement: S1 Appendix — (ZIP) [file pone.0344972.s001.zip › Supporting Information S1/24-003 Post 5 Brodifacoum Midway Island Avian Livers Report.pdf]

To:	Carmen Antaky<br>Biologist<br>NWRC Hawai'i Field Station
Subject:	Determination of brodifacoum in avian livers matrices from Midway Island, Post 5 (QA-3404)
Methods:	188A "Determination of Multiple Rodenticide Residues in Avian Liver by dSPE and LC-MS/MS" -Non-GLP
Analysis Dates:	11/15/23
Notebook References:	AC169, pp.20-21, 44, 49, 53<br>QC35, p.68
Analyst:	Ben Abbo

Sample Description:

Four avian liver samples were submitted on 10/26/23. See sample descriptions on p.3.

Additional Comments:

- Three replicates of each sample were analyzed except for samples S231026-57, -58, and -59. There was insufficient sample to analyze multiple replicates for these samples. A single sample for each of S231026-57 and -58 and two replicates for S231026-59 were analyzed. The mean, standard deviation, and coefficient of variance are reported.
- Control quail liver (S221018-03) was used as the matrix for QC samples.

Method Limit of Detection/Quantitation (MLOD/MLOQ) Values:

Method detection and quantitation limits were determined from by comparing the noise at the analyte retention in three unfortified control quail liver samples to the peak height of brodifacoum in three control quail liver samples fortified to ~15 ng/g brodifacoum. The detection limit was determined to be 3X the noise and the quantitation limit was determined to be 10X the noise found in the unfortified samples.

Method Limit of Detection (MLOD)

Matrix	Detection Limit
Avian Liver	1.3 ng/g

Method Limit of Quantitation (MLOQ)

Matrix	Quantitation Limit
Avian Liver	4.26 ng/g

Results:

Sample ID	Sample Description	Brodifacoum Conc (ng/g)	Descriptive Statistics	
S231026-57-A	Seabird, A-I-Post5-Se, RustyBucket, Bonin	ND	Value=	ND
S231026-57-B	Petrel liver , 10/4/2023	INS	sd=	-----
S231026-57-C		INS	cv=	-----
S231026-58-A	Seabird, A-II-Post5Se, Community Garden,	ND	Value=	ND
S231026-58-B	White Tern liver, 10/11/2023	INS	sd=	-----
S231026-58-C		INS	cv=	-----
S231026-59-A	Shorebird, A-I-Post5-Sh, Hale Honu, PAGP	494	$Mean_2$=	494
S231026-59-B	23-084 liver, 10/3/2023	493	sd=	0.71
S231026-59-C		INS	cv=	0.14%
S231026-60-A	Shorebird, A-VI-Post5-Sh, Catchment,	1010	$Mean_3$=	1030
S231026-60-B	PAGP 23-104 liver, 10/13/2023	1020	sd=	21
S231026-60-C		1050	cv=	2.0%

ND = Not Detected.
INS-Insufficient Sample

QC Results:

ID	Theoretical Brodifacoum Concentration (ng/g)	Observed Brodifacoum Concentration (ng/g)	% Recovery
QC-33	Control	ND	N/A
QC-34	Control	ND	N/A
QC-35	9.59	9.55	99.6
QC-36	9.87	8.89	90.1
QC-37	459	450	98.0
QC-38	478	466	97.5
QC-39	1930	1810	93.8
QC-40	2120	1990	93.9

ND = Not Detected.
