# Supplementary material for: Anticoagulant residues associated with an attempted rodent eradication from a subtropical coral atoll
Source: PLoS One. 2026 Mar 23;21(3):e0344972. doi: 10.1371/journal.pone.0344972 (PMC13008109; doi:10.1371/journal.pone.0344972)
Supplement: S1 Appendix — (ZIP) [file pone.0344972.s001.zip › Supporting Information S1/24-003 Post 5 Brodifacoum Midway Island Crustaceans Report.pdf]

Wildlife Services NWRC National Wildlife Research Center<br>Analytical Services Report	United States Department of Agriculture<br>Animal Plant Health Inspection Service<br>Wildlife Services<br>National Wildlife Research Center<br>Laboratory Support Services Unit	Invoice #: 24-003/3<br>Date: 12/20/23<br>Page: 1 of 3

To: Carmen Antaky
Biologist
NWRC Hawai'i Field Station

Subject: Determination of brodifacoum in crustaceans matrices from Midway Island, Post 5 (QA-3404)

Methods: 188A "Determination of Multiple Rodenticide Residues in Avian Liver by dSPE and LC-MS/MS" -Non-GLP

Analysis Dates: 11/14/23

Notebook References: AC169, pp.20-21, 43, 47, 53
QC35, p.68

Analyst: Ben Abbo

Sample Description:

Six crustacean samples were submitted on 10/26/23. See sample descriptions on p.3.

Additional Comments:

- Three replicates of each sample were analyzed. The mean, standard deviation, and coefficient of variance are reported.
- Control ghost crab (S220801-42) was used as the matrix for QC samples.

Contact the author for further details on QA/QC certification at Carmen.Antaky@usda.gov

Analyst	Date	QC Specialist	Date	Reviewer	Date

Method Limit of Detection/Quantitation (MLOD/MLOQ) Values:

Method detection and quantitation limits were determined from by comparing the noise at the analyte retention in three unfortified control ghost crab samples to the peak height of brodifacoum in three control ghost crab samples fortified to ~15 ng/g brodifacoum. The detection limit was determined to be 3X the noise and the quantitation limit was determined to be 10X the noise found in the unfortified samples.

Method Limit of Detection (MLOD)

Matrix	Detection Limit
Invertebrates	0.94 ng/g

Method Limit of Quantitation (MLOQ)

Matrix	Quantitation Limit
Invertebrates	3.12 ng/g

Results:

Sample ID	Sample Description	Brodifacoum Conc (ng/g)	Descriptive Statistics	
S231026-43-A	Crabs, A-I-Post5-Gh, CargoPier,	10.1	$Mean_3$=	10.2
S231026-43-B	Ghostcrabs, 10/4/2023	9.75	sd=	0.43
S231026-43-C		10.6	cv=	4.2%
S231026-44-A	Crabs, A-II-Post5-Gh, CargoPier,	15.5	$Mean_3$=	14.9
S231026-44-B	Ghostcrabs, 10/4/2023	15.0	sd=	0.71
S231026-44-C		14.1	cv=	4.8%
S231026-45-A	Crabs, B-I-Post5-Gh, Hale Honu,	2.3*	$Mean_3$=	2.5*
S231026-45-B	Ghostcrabs, 10/4/2023	2.8*	sd=	0.29
S231026-45-C		2.3*	cv=	12%
S231026-46-A	Crabs, B-II-Post5-Gh, Hale Honu,	5.33	$Mean_3$=	4.83
S231026-46-B	Ghostcrabs, 10/4/2023	4.39	sd=	0.47
S231026-46-C		4.76	cv=	9.7%
S231026-47-A	Crabs, C-I-Post5-Gh, RustyBucket,	4.98	$Mean_3$=	5.25
S231026-47-B	Ghostcrabs, 10/4/2023	3.40	sd=	2.0
S231026-47-C		7.37	cv=	38%
S231026-48-A	Crabs, C-II-Post5-Gh, RustyBucket,	4.48	$Mean_3$=	4.25
S231026-48-B	Ghostcrabs, 10/4/2023	4.36	sd=	0.29
S231026-48-C		3.92	cv=	6.8%

ND = Not Detected.

QC Results:

ID	Theoretical Brodifacoum Concentration (ng/g)	Observed Brodifacoum Concentration (ng/g)	% Recovery
QC-17	Control	ND	N/A
QC-18	Control	ND	N/A
QC-19	8.89	8.28	93.1
QC-20	8.25	6.90	83.6
QC-21	569	546	96.0
QC-22	491	470	95.7
QC-23	2090	1980	94.7
QC-24	2060	1950	94.7

ND = Not Detected.
