# Supplementary material for: Anticoagulant residues associated with an attempted rodent eradication from a subtropical coral atoll
Source: PLoS One. 2026 Mar 23;21(3):e0344972. doi: 10.1371/journal.pone.0344972 (PMC13008109; doi:10.1371/journal.pone.0344972)
Supplement: S1 Appendix — (ZIP) [file pone.0344972.s001.zip › Supporting Information S1/24-003 Post 5 Brodifacoum Midway Island Fish Report.pdf]

To: Carmen Antaky
Biologist
NWRC Hawai'i Field Station

Subject: Determination of brodifacoum in fish matrices from Midway Island, Post 5 (QA-3404)

Methods: 188A "Determination of Multiple Rodenticide Residues in Avian Liver by dSPE and LC-MS/MS" -Non-GLP

Analysis Dates: 11/14/23

Notebook References: AC169, pp.20-21, 43, 46, 52
QC35, p.68

Analyst: Ben Abbo

Sample Description:

Eighteen fish samples were submitted on 10/26/23. See sample descriptions on pp.3-4.

Additional Comments:

- Three replicates of each sample were analyzed. The mean, standard deviation, and coefficient of variance are reported.
- Control sea bass (S220906-01) was used as the matrix for QC samples.

Method Limit of Detection/Quantitation (MLOD/MLOQ) Values:

Method detection and quantitation limits were determined from by comparing the noise at the analyte retention in three unfortified control sea bass samples to the peak height of brodifacoum in three control sea bass samples fortified to ~15 ng/g brodifacoum. The detection limit was determined to be 3X the noise and the quantitation limit was determined to be 10X the noise found in the unfortified samples.

Method Limit of Detection (MLOD)

Matrix	Detection Limit
Fish	1.2 ng/g

Method Limit of Quantitation (MLOQ)

Matrix	Quantitation Limit
Fish	4.06 ng/g

Results:

Sample ID	Sample Description	Brodifacoum Conc (ng/g)	Descriptive Statistics	
S231026-25-A	Mosquito Fish , A-I-Post5-M, R2,	35.5	$Mean_3$=	37.5
S231026-25-B	Gambusia, 10/10/2023	38.1	sd=	1.7
S231026-25-C		38.8	cv=	4.5%
S231026-26-A	Mosquito Fish , A-II-Post5-M, R2,	14.6	$Mean_3$=	14.7
S231026-26-B	Gambusia, 10/10/2023	14.5	sd=	0.32
S231026-26-C		15.1	cv=	2.2%
S231026-27-A	Mosquito Fish , C-I-Post5-M, Catchment,	27.2	$Mean_3$=	28.6
S231026-27-B	Gambusia, 10/10/2023	30.6	sd=	1.8
S231026-27-C		28.0	cv=	6.3%
S231026-28-A	Mosquito Fish , C-II-Post5-M, Catchment,	20.1	$Mean_3$=	20.1
S231026-28-B	Gambusia, 10/10/2023	19.6	sd=	0.45
S231026-28-C		20.5	cv=	2.24%
S231026-29-A	Mosquito Fish , E-I-Post5-M, Drainage	73.2	$Mean_3$=	78.0
S231026-29-B	Pond, Gambusia, 10/10/2023	76.8	sd=	5.4
S231026-29-C		83.9	cv=	6.9%
S231026-30-A	Mosquito Fish , E-II-Post5-M, Drainage	77.4	$Mean_3$=	77.5
S231026-30-B	Pond, Gambusia, 10/10/2023	81.4	sd=	3.9
S231026-30-C		73.7	cv=	5.0%
S231026-31-A	Bait Fish, A-I-Post5-Bait, Cargo Pier,	23.7	$Mean_3$=	18.9
S231026-31-B	Flagtail, 10/3/2023	16.8	sd=	4.1
S231026-31-C		16.3	cv=	22%
S231026-32-A	Bait Fish, A-II-Post5-Bait, Cargo Pier,	26.7	$Mean_3$=	25.0
S231026-32-B	Flagtail, 10/3/2023	24.7	sd=	1.6
S231026-32-C		23.5	cv=	6.4%
S231026-33-A	Bait Fish, B-I-Post5-Bait, Hale Honu,	8.40	$Mean_3$=	8.49
S231026-33-B	Flagtail, 10/3/2023	8.05	sd=	0.49
S231026-33-C		9.02	cv=	5.8%
S231026-34-A	Bait Fish, B-II-Post5-Bait, Hale Honu,	15.7	$Mean_3$=	15.1
S231026-34-B	Flagtail, 10/3/2023	13.4	sd=	1.5
S231026-34-C		16.2	cv=	9.9%
S231026-35-A	Bait Fish, C-I-Post5-Bait, RustyBucket,	ND	$Mean_3$=	ND
S231026-35-B	Flagtail, 10/4/2023	ND	sd=	-----
S231026-35-C		ND	cv=	-----
S231026-36-A	Bait Fish, C-II-Post5-Bait, RustyBucket,	ND	$Mean_3$=	ND
S231026-36-B	Flagtail, 10/4/2023	ND	sd=	-----
S231026-36-C		ND	cv=	-----

ND = Not Detected.
*-Value is below the method quantitation limit of 4.06 ng/g

Results:

Sample ID	Sample Description	Brodifacoum Conc (ng/g)	Descriptive Statistics	
S231026-37-A	Reef Fish, A-I-Post5-Reef, CargoPier, Moi,	ND	$Mean_3$=	ND
S231026-37-B	10/3/2023	ND	sd=	-----
S231026-37-C		ND	cv=	-----
S231026-38-A	Reef Fish, A-II-Post5-Reef, CargoPier, Moi,	ND	$Mean_3$=	ND
S231026-38-B	10/3/2023	ND	sd=	-----
S231026-38-C		ND	cv=	-----
S231026-39-A	Reef Fish, B-I-Post5-Reef, Hale Honu,	1.4*	$Mean_3$=	ND
S231026-39-B	Goatfish, 10/6/2023	ND	sd=	-----
S231026-39-C		ND	cv=	-----
S231026-40-A	Reef Fish, B-II-Post5-Reef, Hale Honu,	ND	$Mean_3$=	ND
S231026-40-B	Goatfish, 10/6/2023	ND	sd=	-----
S231026-40-C		ND	cv=	-----
S231026-41-A	Reef Fish, C-I-Post5-Reef, RustyBucket,	ND	$Mean_3$=	ND
S231026-41-B	Goatfish, 10/6/2023	ND	sd=	-----
S231026-41-C		ND	cv=	-----
S231026-42-A	Reef Fish, C-II-Post5-Reef, RustyBucket,	ND	$Mean_3$=	ND
S231026-42-B	Goatfish, 10/6/2023	ND	sd=	-----
S231026-42-C		ND	cv=	-----

ND = Not Detected.
*-Value is below the method quantitation limit of 4.06 ng/g

QC Results:

ID	Theoretical Brodifacoum Concentration (ng/g)	Observed Brodifacoum Concentration (ng/g)	% Recovery
QC-09	Control	ND	N/A
QC-10	Control	ND	N/A
QC-11	13.7	12.2	89.1
QC-12	15.9	15.5	97.5
QC-13	502	475	94.5
QC-14	457	432	94.6
QC-15	2310	2160	93.5
QC-16	2000	1870	93.5

ND = Not Detected.
