# Supplementary material for: Anticoagulant residues associated with an attempted rodent eradication from a subtropical coral atoll
Source: PLoS One. 2026 Mar 23;21(3):e0344972. doi: 10.1371/journal.pone.0344972 (PMC13008109; doi:10.1371/journal.pone.0344972)
Supplement: S1 Appendix — (ZIP) [file pone.0344972.s001.zip › Supporting Information S1/24-003 Post 5 Brodifacoum Midway Island Invertebrates Report.pdf]

To: Carmen Antaky
Biologist
NWRC Hawai'i Field Station

Subject: Determination of brodifacoum in invertebrate matrices from Midway Island, Post 5 (QA-3404)

Methods: 188A "Determination of Multiple Rodenticide Residues in Avian Liver by dSPE and LC-MS/MS" -Non-GLP

Analysis Dates: 11/14/23

Notebook References: AC169, pp.20-21, 42, 45, 52
QC35, p.68

Analyst: Ben Abbo

Sample Description:

Twenty-four invertebrate samples were submitted on 10/26/23. See sample descriptions on pp.3-4.

Additional Comments:

- Three replicates of each sample were analyzed, unless there was insufficient sample to analyze three replicates. The mean, standard deviation, and coefficient of variance are reported.
- Control crickets (S221018-01) were used as the matrix for QC samples.

Method Limit of Detection/Quantitation (MLOD/MLOQ) Values:

Method detection and quantitation limits were determined from by comparing the noise at the analyte retention in three unfortified control cricket samples to the peak height of brodifacoum in three control cricket samples fortified to ~15 ng/g brodifacoum. The detection limit was determined to be 3X the noise and the quantitation limit was determined to be 10X the noise found in the unfortified samples.

Method Limit of Detection (MLOD)

Matrix	Detection Limit
Invertebrates	1.2 ng/g

Method Limit of Quantitation (MLOQ)

Matrix	Quantitation Limit
Invertebrates	3.97 ng/g

Results:

Sample ID	Sample Description	Brodifacoum Conc (ng/g)	Descriptive Statistics	
S231026-01-A	Grubs, A-I-Post5-G, Radar, Emerald beetle, 10/4/2023	6.42	$Mean_3$=	6.01
S231026-01-B		6.17	sd=	0.51
S231026-01-C		5.44	cv=	8.5%
S231026-02-A	Grubs, A-II-Post5-G, Radar, Emerald beetle, 10/4/2023	4.48	$Mean_3$=	3.9*
S231026-02-B		3.7*	sd=	0.56
S231026-02-C		3.4*	cv=	14%
S231026-03-A	Grubs, B-I-Post5-G, Brackish, Emerald beetle, 10/4/2023	3.3*	$Mean_3$=	3.2*
S231026-03-B		2.9*	sd=	0.31
S231026-03-C		3.5*	cv=	9.7%
S231026-04-A	Grubs, B-II-Post5-G, Brackish, Emerald beetle, 10/4/2023	11.5	$Mean_3$=	11.3
S231026-04-B		11.3	sd=	0.15
S231026-04-C		11.2	cv=	1.3%
S231026-05-A	Grubs, C-I-Post5-G, RustyBucket, Emerald beetle, 10/4/2023	3.9*	$Mean_3$=	4.10
S231026-05-B		4.29	sd=	0.20
S231026-05-C		4.12	cv=	4.9%
S231026-06-A	Grubs, C-II-Post5-G, RustyBucket, Emerald beetle, 10/4/2023	2.3*	$Mean_3$=	1.8*
S231026-06-B		1.3*	sd=	0.50
S231026-06-C		1.8*	cv=	28%
S231026-07-A	Roaches, A-I-Post5-R, Radar, Blattodea, 10/15/2023	ND	$Mean_3$=	ND
S231026-07-B		ND	sd=	-----
S231026-07-C		ND	cv=	-----
S231026-08-A	Roaches, A-II-Post5-R, Radar, Blattodea, 10/15/2023	ND	$Mean_3$=	ND
S231026-08-B		ND	sd=	-----
S231026-08-C		ND	cv=	-----
S231026-09-A	Roaches, B-I-Post5-R, Brackish, Blattodea, 10/15/2023	9.89	$Mean_3$=	8.64
S231026-09-B		7.01	sd=	1.5
S231026-09-C		9.02	cv=	17%
S231026-10-A	Roaches, B-II-Post5-R, Brackish, Blattodea, 10/15/2023	3.9*	$Mean_3$=	3.7*
S231026-10-B		3.5*	sd=	0.20
S231026-10-C		3.7*	cv=	5.4%
S231026-11-A	Roaches, C-I-Post5-R, RustyBucket, Blattodea, 10/15/2023	3.3*	$Mean_3$=	2.8*
S231026-11-B		2.7*	sd=	0.46
S231026-11-C		2.4*	cv=	16%
S231026-12-A	Roaches, C-II-Post5-R, RustyBucket, Blattodea, 10/15/2023	4.16	$Mean_3$=	4.53
S231026-12-B		4.79	sd=	0.33
S231026-12-C		4.63	cv=	7.3%

ND = Not Detected.

*-Value is below the method quantitation limit of 3.97 ng/g

Results:

Sample ID	Sample Description	Brodifacoum Conc (ng/g)	Descriptive Statistics	
S231026-13-A	Roaches, D-I-Post5-R, Ballfield, Blattodea, 10/16/2023	3.9*	$Mean_3$=	3.8*
S231026-13-B		3.7*	sd=	0.10
S231026-13-C		3.8*	cv=	2.6%
S231026-14-A	Roaches, D-II-Post5-R, Ballfield, Blattodea, 10/16/2023	2.2*	$Mean_3$=	2.5*
S231026-14-B		2.9*	sd=	0.35
S231026-14-C		2.5*	cv=	14%
S231026-15-A	Roaches, E-I-Post5-R, Parade Ground, Blattodea, 10/16/2023	2.2*	$Mean_3$=	2.5*
S231026-15-B		2.9*	sd=	0.35
S231026-15-C		2.5*	cv=	14%
S231026-16-A	Roaches, E-II-Post5-R, Parade Ground, Blattodea, 10/16/2023	3.1*	$Mean_3$=	3.5*
S231026-16-B		4.06	sd=	0.51
S231026-16-C		3.3*	cv=	15%
S231026-17-A	Roaches, F-I-Post5-R, SE Corner Radar Hill, Blattodea, 10/15/2023	4.66	$Mean_3$=	5.21
S231026-17-B		5.76	sd=	0.55
S231026-17-C		5.22	cv=	11%
S231026-18-A	Roaches, F-II-Post5-R, SE Corner Radar Hill, Blattodea, 10/15/2023	2.6*	$Mean_3$=	2.6*
S231026-18-B		2.7*	sd=	0.058
S231026-18-C		2.6*	cv=	2.2%
S231026-19-A	Pitfall, A-I-Post5-Pitfall, Radar, Pooled, 10/4/2023	5.61	$Mean_3$=	5.34
S231026-19-B		4.96	sd=	0.34
S231026-19-C		5.45	cv=	6.4%
S231026-20-A	Pitfall, A-II-Post5-Pitfall, Radar, Pooled, 10/4/2023	4.20	$Mean_3$=	3.7*
S231026-20-B		3.5*	sd=	0.47
S231026-20-C		3.3*	cv=	13%
S231026-21-A	Pitfall, B-I-Post5-Pitfall, Brackish, Pooled, 10/4/2023	18.7	$Mean_3$=	20.2
S231026-21-B		21.7	sd=	2.1
S231026-21-C		INS	cv=	10%
S231026-22-A	Pitfall, B-II-Post5-Pitfall, Brackish, Pooled, 10/4/2023	105	$Mean_3$=	100
S231026-22-B		96.7	sd=	4.2
S231026-22-C		99.3	cv=	4.2%
S231026-23-A	Pitfall, C-I-Post5-Pitfall, RustyBucket, Pooled, 10/6/2023	3.2*	$Mean_3$=	2.7*
S231026-23-B		2.4*	sd=	0.44
S231026-23-C		2.5*	cv=	16%
S231026-24-A	Pitfall, C-II-Post5-Pitfall, RustyBucket, Pooled, 10/6/2023	4.06	$Mean_3$=	3.6*
S231026-24-B		2.7*	sd=	0.81
S231026-24-C		4.14	cv=	23%

ND = Not Detected.
*-Value is below the method quantitation limit of 3.97 ng/g
INS-Insufficient Sample

QC Results:

ID	Theoretical Brodifacoum Concentration (ng/g)	Observed Brodifacoum Concentration (ng/g)	% Recovery
QC-01	Control	ND	N/A
QC-02	Control	ND	N/A
QC-03	12.2	12.1	99.2
QC-04	12.4	11.7	94.4
QC-05	533	520	97.6
QC-06	523	465	94.6
QC-07	1990	1940	97.5
QC-08	2110	2000	94.8

ND = Not Detected.
