# Supplementary material for: Anticoagulant residues associated with an attempted rodent eradication from a subtropical coral atoll
Source: PLoS One. 2026 Mar 23;21(3):e0344972. doi: 10.1371/journal.pone.0344972 (PMC13008109; doi:10.1371/journal.pone.0344972)
Supplement: S1 Appendix — (ZIP) [file pone.0344972.s001.zip › Supporting Information S1/24-003 Post 5 Brodifacoum Midway Island Pellets Report.pdf]

To: Carmen Antaky
Biologist
NWRC Hawai'i Field Station

Subject: Determination of brodifacoum in pellet matrices from Midway Island, Post 5 (QA-3404)

Methods: 188A "Determination of Multiple Rodenticide Residues in Avian Liver by dSPE and LC-MS/MS" -Non-GLP

Analysis Dates: 11/15/23, 11/16/23

Notebook References: AC169, pp.20-21, 44, 50, 53-54
QC35, p.68

Analyst: Ben Abbo

Sample Description:

Two pellet samples were submitted on 10/26/23. See sample descriptions on p.3.

Additional Comments:

- Samples and QC samples were initially extracted on 11/15/23. The field samples were both beyond the calibration curve, thus it was not possible to accurately quantify the samples. They were diluted and reanalyzed on 11/16/23.
- Three replicates of each sample were analyzed. The mean, standard deviation, and coefficient of variance are reported.
- Control brodifacoum pellet bait (S181102-02) was used as the matrix for QC samples.

Method Limit of Detection/Quantitation (MLOD/MLOQ) Values:

Method detection and quantitation limits were determined from by comparing the noise at the analyte retention in two unfortified control pellet samples to the peak height of brodifacoum in two control pellet samples fortified to ~15 ng/g brodifacoum. The detection limit was determined to be 3X the noise and the quantitation limit was determined to be 10X the noise found in the unfortified samples.

Method Limit of Detection (MLOD)

Matrix	Detection Limit
Pellet	2.7 ng/g

Method Limit of Quantitation (MLOQ)

Matrix	Quantitation Limit
Pellet	8.95 ng/g

Results:

Sample ID	Sample Description	Brodifacoum Conc (ng/g)	Descriptive Statistics	
S231026-65-A	Pellets, A-I-Post5-Pel, Cargo Pier,	22400	$Mean_3$=	22100
S231026-65-B	10/10/2023	23000	sd=	1000
S231026-65-C		21000	cv=	4.5%
S231026-66-A	Pellets, B-II-Post5-Pel, North Beach,	11640	$Mean_3$=	12200
S231026-66-B	10/10/2023	12720	sd=	550
S231026-66-C		12380	cv=	4.5%

QC Results:

ID	Theoretical Brodifacoum Concentration (ng/g)	Observed Brodifacoum Concentration (ng/g)	% Recovery
QC-49	Control	ND	N/A
QC-50	Control	ND	N/A
QC-51	9.20	8.97	97.5
QC-52	9.94	9.01	90.6
QC-53	520	503	96.7
QC-54	512	482	94.1
QC-55	2230	2100	94.2
QC-56	2260	2060	91.2

ND = Not Detected.
