# Supplementary material for: Anticoagulant residues associated with an attempted rodent eradication from a subtropical coral atoll
Source: PLoS One. 2026 Mar 23;21(3):e0344972. doi: 10.1371/journal.pone.0344972 (PMC13008109; doi:10.1371/journal.pone.0344972)
Supplement: S1 Appendix — (ZIP) [file pone.0344972.s001.zip › Supporting Information S1/24-003 Post 5 Brodifacoum Midway Island Soils Report.pdf]

Wildlife Services NWRC National Wildlife Research Center<br>Analytical Services Report	United States Department of Agriculture<br>Animal Plant Health Inspection Service<br>Wildlife Services<br>National Wildlife Research Center<br>Laboratory Support Services Unit	Invoice #: 24-003/6<br>Date: 12/20/23<br>Page: 1 of 4

To: Carmen Antaky
Biologist
NWRC Hawai'i Field Station

Subject: Determination of brodifacoum in soil matrices from Midway Island, Post 5 (QA-3404)

Methods: 188A "Determination of Multiple Rodenticide Residues in Avian Liver by dSPE and LC-MS/MS" -Non-GLP

Analysis Dates: 11/15/23

Notebook References: AC169, pp.20-21, 44, 50, 53
QC35, p.68

Analyst: Ben Abbo

Sample Description:

Four soil samples were submitted on 10/26/23. See sample descriptions on p.3.

Additional Comments:

- Three replicates of each sample were analyzed. The mean, standard deviation, and coefficient of variance are reported.
- Control soil (S220801-61) was used as the matrix for QC samples.

Method Limit of Detection/Quantitation (MLOD/MLOQ) Values:

Method detection and quantitation limits were determined from by comparing the noise at the analyte retention in three unfortified control soil samples to the peak height of brodifacoum in three control soil samples fortified to ~15 ng/g brodifacoum. The detection limit was determined to be 3X the noise and the quantitation limit was determined to be 10X the noise found in the unfortified samples.

Method Limit of Detection (MLOD)

Matrix	Detection Limit
Soil	1.3 ng/g

Method Limit of Quantitation (MLOQ)

Matrix	Quantitation Limit
Soil	4.29 ng/g

Results:

Sample ID	Sample Description	Brodifacoum Conc (ng/g)	Descriptive Statistics	
S231026-61-A	Soil, A-I-Post5-SS, Parade Ground, 10/11/2023	2.1*	$Mean_3$=	1.6*
S231026-61-B		ND	sd=	0.79
S231026-61-C		1.9*	cv=	49%
S231026-62-A	Soil, B-I-Post5-SS, Orchard, 10/11/2023	ND	$Mean_3$=	2.2*
S231026-62-B		5.20	sd=	2.6
S231026-62-C		ND	cv=	118%
S231026-63-A	Soil, C-I-Post5-SS, Community Garden, 10/11/2023	ND	$Mean_3$=	ND
S231026-63-B		ND	sd=	-----
S231026-63-C		ND	cv=	-----
S231026-64-A	Soil, D-I-Post5-SS, Ballfield, 10/11/2023	ND	$Mean_3$=	ND
S231026-64-B		ND	sd=	-----
S231026-64-C		ND	cv=	-----

ND = Not Detected.
*-Sample was below quantitation limit of 4.29 ng/g; result should be considered qualitative.
For samples that had replicates with detectable levels of brodifacoum and replicates that did not, the mean is calculated by assigning a value of 0.65 (half of the detection limit) to the non-dectected samples.

QC Results:

ID	Theoretical Brodifacoum Concentration (ng/g)	Observed Brodifacoum Concentration (ng/g)	% Recovery
QC-41	Control	ND	N/A
QC-42	Control	ND	N/A
QC-43	9.46	7.65	80.9
QC-44	8.83	7.69	87.1
QC-45	473	450	95.1
QC-46	533	493	92.5
QC-47	2450	2330	95.1
QC-48	2440	2320	95.1

ND = Not Detected.
