# Supplementary material for: Anticoagulant residues associated with an attempted rodent eradication from a subtropical coral atoll
Source: PLoS One. 2026 Mar 23;21(3):e0344972. doi: 10.1371/journal.pone.0344972 (PMC13008109; doi:10.1371/journal.pone.0344972)
Supplement: S1 Appendix — (ZIP) [file pone.0344972.s001.zip › Supporting Information S1/24-003 Post 5 Brodifacoum Midway Island Waters Report.pdf]

Wildlife Services **NWRC** National Wildlife Research Center<br>Analytical Services Report	United States Department of Agriculture<br>Animal Plant Health Inspection Service<br>Wildlife Services<br>National Wildlife Research Center<br>Laboratory Support Services Unit	Invoice #: 23-034/8<br>Date: 12/20/23<br>Page: 1 of 3

To: Carmen Antaky
Biologist
NWRC Hawai'i Field Station

Subject: Determination of brodifacoum in Waters from Midway Island, Post 5 (QA-3404)

Methods: 188A "Determination of Multiple Rodenticide Residues in Avian Liver by dSPE and LC-MS/MS" -Non-GLP
"Determination of Brodifacoum Residues in Water"-Non-GLP

Analysis Dates: 11/16/23

Notebook References: AC169, pp.20-21, 55
QC35, p.68

Analyst: Ben Abbo

Sample Description:

Two samples of water from Midway Atoll were submitted on 10/26/23. See sample descriptions on p.3.

Additional Comments:

- Two replicates of each sample were analyzed.
- Cache la Poudre River water from Laporte, Colorado (S230825-01) was used as the matrix for QC samples for fresh water.

Contact the author for further details on QA/QC certification at Carmen.Antaky@usda.gov

Analyst	Date	QC Specialist	Date	Reviewer	Date

Sample Preparation and Extraction:

Water Extraction Procedure:

1. Add 60 mL of seawater to a 125-mL separatory funnel.
2. Add 0.020 mL surrogate to all and 0.040 mL of acetonitrile or 75X brodifacoum stock as indicated.
3. Add 20 mL chloroform.
4. Add ~8.5g sodium chloride.
5. Add 10 mL 1M hydrochloric acid.
6. Cap and shake for 8-10s, let set 1 minute. Repeat 2X.
7. Dispense chloroform phase into 25-mL glass tube taking care to not transfer any water phase.
8. Remove solvent in a 60°C N-Evap with a gentle flow of nitrogen gas.
9. Add 0.300 mL acetonitrile, vortex thoroughly, wetting as much of the inside surface of the tube as possible.
10. Add 1.200 mL pH 9.5 20-mM ammonium acetate buffer and vortex thoroughly.
11. Transfer the sample to an autosampler vial and assay by LC-MS/MS.

Method Limit of Detection/Quantitation (MLOD/MLOQ) Values:

Method detection and quantitation limits were determined by comparing the noise at the analyte retention in two unfortified control water samples to the peak height of brodifacoum in two control water samples fortified to 0.0214 ng/mL brodifacoum. The detection limit was determined to be 3X the noise and the quantitation limit was determined to be 10X the noise found in the unfortified samples.

Method Limit of Detection (MLOD)

**Matrix**	**Detection Limit**
Fresh water	0.0071 ng/mL
Sea water	0.0014 ng/mL

Method Limit of Quantitation (MLOQ)

**Matrix**	**Quantitation Limit**
Fresh water	0.0236 ng/mL
Sea water	0.00453 ng/mL

Results:

Fresh Water

Sample ID	Sample Description	Brodifacoum (ng/g)	Descriptive Statistics	
S231026-55-A	Drainage Water, A-I-Post5-WC, Catchment,	ND	Avg_2=	ND
S231026-55-B	catchment drainage grate, 10/10/2023	ND	sd=	------
			cv=	------
S231026-56-A	Drainage Water, C-I-Post5-WC, Catchment,	ND	Avg_2=	ND
S231026-56-B	catchment pond, 10/10/2023	ND	sd=	------
			cv=	------

ND-Not Detected

QC Results:

Fresh Water:

ID	Theoretical Brodifacoum Concentration (ng/mL)	Observed Brodifacoum Concentration (ng/mL)	% Recovery
QC-85	Control	ND	-----
QC-86	Control	ND	-----
QC-87	0.0214	0.018*	84.1
QC-88	0.0214	0.020*	93.5
QC-89	1.73	1.61	93.1
QC-90	1.73	1.67	96.5
QC-91	5.20	4.88	93.8
QC-92	5.20	4.91	94.4

ND-Not Detected
*-Value is below the quantitation limit of 0.0236 ng/mL
