# Supplementary material for: Anticoagulant residues associated with an attempted rodent eradication from a subtropical coral atoll
Source: PLoS One. 2026 Mar 23;21(3):e0344972. doi: 10.1371/journal.pone.0344972 (PMC13008109; doi:10.1371/journal.pone.0344972)
Supplement: S1 Appendix — (ZIP) [file pone.0344972.s001.zip › Supporting Information S1/24-009 Post 6 Brodifacoum Midway Island Avian Livers Report.pdf]

To:	Carmen Antaky<br>Biologist<br>NWRC Hawai'i Field Station
Subject:	Determination of brodifacoum in avian livers matrices from Midway Island, Post 6 (QA-3404)
Methods:	188A "Determination of Multiple Rodenticide Residues in Avian Liver by dSPE and LC-MS/MS" -Non-GLP
Analysis Dates:	12/15/23
Notebook References:	AC169, pp.20-21, 59, 62, 64<br>QC35, p.68
Analyst:	Ben Abbo

Sample Description:

One avian liver sample was submitted on 12/08/23. See sample description on p.2.

Additional Comments:

- Two replicates of the sample were analyzed due to not having sufficient sample to prepare three. The mean, standard deviation, and coefficient of variance are reported.
- Control quail liver (S221018-03) was used as the matrix for QC samples.

Method Limit of Detection/Quantitation (MLOD/MLOQ) Values:

Method detection and quantitation limits were determined from by comparing the noise at the analyte retention in three unfortified control quail liver samples to the peak height of brodifacoum in three control quail liver samples fortified to ~15 ng/g brodifacoum. The detection limit was determined to be 3X the noise and the quantitation limit was determined to be 10X the noise found in the unfortified samples.

Method Limit of Detection (MLOD)

Matrix	Detection Limit
Avian Liver	1.3 ng/g

Method Limit of Quantitation (MLOQ)

Matrix	Quantitation Limit
Avian Liver	4.26 ng/g

Results:

Sample ID	Sample Description	Brodifacoum Conc (ng/g)	Descriptive Statistics	
S231208-10-A	Shorebird, A-I-Post6-Sh, liver	92.6	$Mean_2$=	93.5
S231208-10-B		94.3	sd=	1.2
			cv=	1.3%

QC Results:

ID	Theoretical Brodifacoum Concentration (ng/g)	Observed Brodifacoum Concentration (ng/g)	% Recovery
QC-17	Control	ND	N/A
QC-18	Control	ND	N/A
QC-19	14.0	12.8	91.4
QC-20	13.7	12.2	89.1
QC-21	436	415	95.2
QC-22	499	474	95.0
QC-23	2250	2120	94.2
QC-24	2250	2110	93.8

ND = Not Detected.
