# Supplementary material for: Anticoagulant residues associated with an attempted rodent eradication from a subtropical coral atoll
Source: PLoS One. 2026 Mar 23;21(3):e0344972. doi: 10.1371/journal.pone.0344972 (PMC13008109; doi:10.1371/journal.pone.0344972)
Supplement: S1 Appendix — (ZIP) [file pone.0344972.s001.zip › Supporting Information S1/24-009 Post 6 Brodifacoum Midway Island Fish Report.pdf]

Wildlife Services NWRC National Wildlife Research Center<br>Analytical Services Report	United States Department of Agriculture<br>Animal Plant Health Inspection Service<br>Wildlife Services<br>National Wildlife Research Center<br>Laboratory Support Services Unit	Invoice #: 24-009/2<br>Date: 01/22/24<br>Page: 1 of 3

To: Carmen Antaky
Biologist
NWRC Hawai'i Field Station

Subject: Determination of brodifacoum in fish matrices from Midway Island, Post 6 (QA-3404)

Methods: 188A "Determination of Multiple Rodenticide Residues in Avian Liver by dSPE and LC-MS/MS" -Non-GLP

Analysis Dates: 12/15/23

Notebook References: AC169, pp.20-21, 59, 61, 64
QC35, p.68

Analyst: Ben Abbo

Sample Description:

Three fish samples were submitted on 12/08/23. See sample descriptions on p.3.

Additional Comments:

- Three replicates of each sample were analyzed. The mean, standard deviation, and coefficient of variance are reported.
- Control sea bass (S220906-01) was used as the matrix for QC samples.

Method Limit of Detection/Quantitation (MLOD/MLOQ) Values:

Method detection and quantitation limits were determined from by comparing the noise at the analyte retention in three unfortified control sea bass samples to the peak height of brodifacoum in three control sea bass samples fortified to ~15 ng/g brodifacoum. The detection limit was determined to be 3X the noise and the quantitation limit was determined to be 10X the noise found in the unfortified samples.

Method Limit of Detection (MLOD)

**Matrix**	**Detection Limit**
Fish	1.2 ng/g

Method Limit of Quantitation (MLOQ)

**Matrix**	**Quantitation Limit**
Fish	4.06 ng/g

Results:

Sample ID	Sample Description	Brodifacoum Conc (ng/g)	Descriptive Statistics	
S231208-07-A	Mosquito Fish , A-I-Post6-M, R2, Gambusia	19.4	$Mean_3$=	19.0
S231208-07-B		19.9	sd=	1.1
S231208-07-C		17.8	cv=	5.8%
S231208-08-A	Mosquito Fish , B-I-Post6-M, Catchment,	ND	$Mean_3$=	ND
S231208-08-B	Gambusia	ND	sd=	------
S231208-08-C		ND	cv=	------
S231208-09-A	Mosquito Fish , C-I-Post6-M, Drainage	33.2	$Mean_3$=	33.0
S231208-09-B	Pond, Gambusia	32.9	sd=	0.17
S231208-09-C		32.9	cv=	0.52%

ND = Not Detected.

QC Results:

ID	Theoretical Brodifacoum Concentration (ng/g)	Observed Brodifacoum Concentration (ng/g)	% Recovery
QC-09	Control	ND	N/A
QC-10	Control	ND	N/A
QC-11	14.9	13.4	89.9
QC-12	13.8	13.0	94.2
QC-13	545	520	95.4
QC-14	476	454	95.4
QC-15	2330	2160	92.7
QC-16	2050	1930	94.1

ND = Not Detected.
