# Supplementary material for: Anticoagulant residues associated with an attempted rodent eradication from a subtropical coral atoll
Source: PLoS One. 2026 Mar 23;21(3):e0344972. doi: 10.1371/journal.pone.0344972 (PMC13008109; doi:10.1371/journal.pone.0344972)
Supplement: S1 Appendix — (ZIP) [file pone.0344972.s001.zip › Supporting Information S1/24-018 Post 7 Brodifacoum Midway Island Avian Livers Report.pdf]

Wildlife Services NWRC National Wildlife Research Center<br>Analytical Services Report	United States Department of Agriculture<br>Animal Plant Health Inspection Service<br>Wildlife Services<br>National Wildlife Research Center<br>Laboratory Support Services Unit	Invoice #: 24-018/3<br>Date: 03/01/24<br>Page: 1 of 2

To: Carmen Antaky
Biologist
NWRC Hawai'i Field Station

Subject: Determination of brodifacoum in avian livers matrices from Midway Island, Post 7 (QA-3404)

Methods: 188A "Determination of Multiple Rodenticide Residues in Avian Liver by dSPE and LC-MS/MS" -Non-GLP

Analysis Dates: 02/27/24

Notebook References: AC169, pp.20, 96-103
QC35, p.68

Analyst: Ben Abbo

Sample Description:

One avian liver sample was submitted on 02/15/24. See sample description on p.2.

Additional Comments:

- There was only sufficient sample to do one analysis. The sample was analyzed in its entirety.
- Control quail liver (S221018-03) was used as the matrix for QC samples.

Contact the author for further details on QA/QC certification at Carmen.Antaky@usda.gov

Analyst	Date	QC Specialist	Date	Reviewer	Date

Method Limit of Detection/Quantitation (MLOD/MLOQ) Values:

Method detection and quantitation limits were determined from by comparing the noise at the analyte retention in three unfortified control quail liver samples to the peak height of brodifacoum in three control quail liver samples fortified to ~15 ng/g brodifacoum. The detection limit was determined to be 3X the noise and the quantitation limit was determined to be 10X the noise found in the unfortified samples.

Method Limit of Detection (MLOD)

Matrix	Detection Limit
Avian Liver	1.3 ng/g

Method Limit of Quantitation (MLOQ)

Matrix	Quantitation Limit
Avian Liver	4.26 ng/g

Results:

Sample ID	Sample Description	Brodifacoum Conc (ng/g)	Descriptive Statistics	
S240215-09-A	A-I-Post7-C, Sec 22/Sec 19 Border Rd	2.0*	Value=	2.0*
			sd=	------
			cv=	------
*Value is below the method quantitation limit of 4.26 ng/g				

QC Results:

ID	Theoretical Brodifacoum Concentration (ng/g)	Observed Brodifacoum Concentration (ng/g)	% Recovery
QC-17	Control	ND	N/A
QC-18	Control	ND	N/A
QC-19	14.8	14.4	97.3
QC-20	13.2	13.4	102
QC-21	545	550	101
QC-22	510	510	100
QC-23	2480	2470	99.6
QC-24	2520	2520	100
ND = Not Detected.			
