# Supplementary material for: Anticoagulant residues associated with an attempted rodent eradication from a subtropical coral atoll
Source: PLoS One. 2026 Mar 23;21(3):e0344972. doi: 10.1371/journal.pone.0344972 (PMC13008109; doi:10.1371/journal.pone.0344972)
Supplement: S1 Appendix — (ZIP) [file pone.0344972.s001.zip › Supporting Information S1/24-018 Post 7 Brodifacoum Midway Island Crabs Report.pdf]

Wildlife Services NWRC National Wildlife Research Center Analytical Services Report	United States Department of Agriculture<br>Animal Plant Health Inspection Service<br>Wildlife Services<br>National Wildlife Research Center<br>Laboratory Support Services Unit	Invoice #: 24-018/4<br>Date: 03/01/24<br>Page: 1 of 3

To: Carmen Antaky
Biologist
NWRC Hawai'i Field Station

Subject: Determination of brodifacoum in crustaceans matrices from Midway Island, Post 7 (QA-3404)

Methods: 188A "Determination of Multiple Rodenticide Residues in Avian Liver by dSPE and LC-MS/MS" -Non-GLP

Analysis Dates: 02/27/24

Notebook References: AC169, pp.20, 96-103
QC35, p.68

Analyst: Ben Abbo

Sample Description:

Three crustacean samples were submitted on 02/15/24. See sample descriptions on p.3.

Additional Comments:

- Three replicates of each sample were analyzed. The mean, standard deviation, and coefficient of variance are reported.
- Replicates that are reported as ND are treated as having a value of 1/2 the detection limit (0.47 ng/g) when averaging replicates to determine the sample mean. If the mean is below the detection limit (0.94 ng/g) the sample is reported as ND.
- Control ghost crab (S220801-42) was used as the matrix for QC samples.

Contact the author for further details on QA/QC certification at Carmen.Antaky@usda.gov

Analyst	Date	QC Specialist	Date	Reviewer	Date

Method Limit of Detection/Quantitation (MLOD/MLOQ) Values:

Method detection and quantitation limits were determined from by comparing the noise at the analyte retention in three unfortified control ghost crab samples to the peak height of brodifacoum in three control ghost crab samples fortified to ~15 ng/g brodifacoum. The detection limit was determined to be 3X the noise and the quantitation limit was determined to be 10X the noise found in the unfortified samples.

Method Limit of Detection (MLOD)

Matrix	Detection Limit
Invertebrates	0.94 ng/g

Method Limit of Quantitation (MLOQ)

Matrix	Quantitation Limit
Invertebrates	3.12 ng/g

Results:

Sample ID	Sample Description	Brodifacoum Conc (ng/g)	Descriptive Statistics	
S240215-10-A	A-I-Post7-Gh, CargoPier, Crabs	6.66	$Mean_3$=	6.45
S240215-10-B		5.94	sd=	0.45
S240215-10-C		6.76	cv=	7.0%
S240215-11-A	B-I-Post7-Gh, Hale Honu, Crabs	ND	$Mean_3$=	ND
S240215-11-B		ND	sd=	------
S240215-11-C		ND	cv=	------
S240215-12-A	C-I-Post7-Gh, RustyBucket, Crabs	1.0*	$Mean_3$=	ND
S240215-12-B		1.1*	sd=	------
S240215-12-C		ND	cv=	------

ND = Not Detected.
*-Value is below the method quantitation limit of 3.12 ng/g

QC Results:

ID	Theoretical Brodifacoum Concentration (ng/g)	Observed Brodifacoum Concentration (ng/g)	% Recovery
QC-25	Control	ND	N/A
QC-26	Control	ND	N/A
QC-27	13.3	12.2	91.7
QC-28	15.3	15.0	98.0
QC-29	445	446	100
QC-30	499	499	100
QC-31	2360	2410	102
QC-32	2140	2160	101

ND = Not Detected.
