# Supplementary material for: Anticoagulant residues associated with an attempted rodent eradication from a subtropical coral atoll
Source: PLoS One. 2026 Mar 23;21(3):e0344972. doi: 10.1371/journal.pone.0344972 (PMC13008109; doi:10.1371/journal.pone.0344972)
Supplement: S1 Appendix — (ZIP) [file pone.0344972.s001.zip › Supporting Information S1/24-018 Post 7 Brodifacoum Midway Island Fish Report.pdf]

To:	Carmen Antaky<br>Biologist<br>NWRC Hawai'i Field Station
Subject:	Determination of brodifacoum in fish matrices from Midway Island, Post 7 (QA-3404)
Methods:	188A "Determination of Multiple Rodenticide Residues in Avian Liver by dSPE and LC-MS/MS" -Non-GLP
Analysis Dates:	02/27/24
Notebook References:	AC169, pp.20, 96-103<br>QC35, p.68
Analyst:	Ben Abbo

Sample Description:

Two fish samples were submitted on 02/15/24. See sample descriptions on p.3.

Additional Comments:

- Three replicates of each sample were analyzed. The mean, standard deviation, and coefficient of variance are reported.
- Control sea bass (S220906-01) was used as the matrix for QC samples.

Method Limit of Detection/Quantitation (MLOD/MLOQ) Values:

Method detection and quantitation limits were determined from by comparing the noise at the analyte retention in three unfortified control sea bass samples to the peak height of brodifacoum in three control sea bass samples fortified to ~15 ng/g brodifacoum. The detection limit was determined to be 3X the noise and the quantitation limit was determined to be 10X the noise found in the unfortified samples.

Method Limit of Detection (MLOD)

Matrix	Detection Limit
Fish	1.2 ng/g

Method Limit of Quantitation (MLOQ)

Matrix	Quantitation Limit
Fish	4.06 ng/g

Results:

Sample ID	Sample Description	Brodifacoum Conc (ng/g)	Descriptive Statistics	
S240215-07-A	A-I-Post7-M, R2, Mosquito Fish	55.1	$Mean_3$=	54.5
S240215-07-B		53.5	sd=	0.87
S240215-07-C		54.9	cv=	1.6%
S240215-08-A	B-I-Post7-M, Catchment, Mosquito Fish	3.0*	$Mean_3$=	3.2*
S240215-08-B		3.4*	sd=	0.21
S240215-08-C		3.1*	cv=	6.6%

ND = Not Detected.
*-Value is below the method quantitation limit of 4.06 ng/g

QC Results:

ID	Theoretical Brodifacoum Concentration (ng/g)	Observed Brodifacoum Concentration (ng/g)	% Recovery
QC-09	Control	ND	N/A
QC-10	Control	ND	N/A
QC-11	13.4	14.5	108
QC-12	12.8	11.6	90.6
QC-13	525	534	102
QC-14	542	546	101
QC-15	2050	2040	99.5
QC-16	2240	2200	98.2

ND = Not Detected.
