# Supplementary material for: Anticoagulant residues associated with an attempted rodent eradication from a subtropical coral atoll
Source: PLoS One. 2026 Mar 23;21(3):e0344972. doi: 10.1371/journal.pone.0344972 (PMC13008109; doi:10.1371/journal.pone.0344972)
Supplement: S1 Appendix — (ZIP) [file pone.0344972.s001.zip › Supporting Information S1/24-018 Post 7 Brodifacoum Midway Island Invertebrates Report.pdf]

Wildlife Services NWRC National Wildlife Research Center<br>Analytical Services Report	United States Department of Agriculture<br>Animal Plant Health Inspection Service<br>Wildlife Services<br>National Wildlife Research Center<br>Laboratory Support Services Unit	Invoice #: 24-018/1<br>Date: 03/01/24<br>Page: 1 of 3

To:	Carmen Antaky<br>Biologist<br>NWRC Hawai'i Field Station
Subject:	Determination of brodifacoum in invertebrate matrices from Midway Island, Post 7 (QA-3404)
Methods:	188A "Determination of Multiple Rodenticide Residues in Avian Liver by dSPE and LC-MS/MS" -Non-GLP
Analysis Dates:	02/27/24
Notebook References:	AC169, pp.20, 96-103<br>QC35, p.68
Analyst:	Ben Abbo

Sample Description:

Six invertebrate samples were submitted on 02/15/24. See sample descriptions on p.3.

Additional Comments:

- Three replicates of each sample were analyzed. The mean, standard deviation, and coefficient of variance are reported.
- Control crickets (S221018-01) were used as the matrix for QC samples.

Method Limit of Detection/Quantitation (MLOD/MLOQ) Values:

Method detection and quantitation limits were determined from by comparing the noise at the analyte retention in three unfortified control cricket samples to the peak height of brodifacoum in three control cricket samples fortified to ~15 ng/g brodifacoum. The detection limit was determined to be 3X the noise and the quantitation limit was determined to be 10X the noise found in the unfortified samples.

Method Limit of Detection (MLOD)

Matrix	Detection Limit
Invertebrates	1.2 ng/g

Method Limit of Quantitation (MLOQ)

Matrix	Quantitation Limit
Invertebrates	3.97 ng/g

Results:

Sample ID	Sample Description	Brodifacoum Conc (ng/g)	Descriptive Statistics	
S240215-01-A	A-I-Post7-R, Radar, Roaches	ND	$Mean_3$=	ND
S240215-01-B		ND	sd=	------
S240215-01-C		ND	cv=	------
S240215-02-A	B-I-Post7-R, Brackish, Roaches	4.25	$Mean_3$=	4.20
S240215-02-B		4.27	sd=	0.10
S240215-02-C		4.08	cv=	2.4%
S240215-03-A	C-I-Post7-R, RustyBucket, Roaches	1.8*	$Mean_3$=	1.5*
S240215-03-B		1.5*	sd=	0.30
S240215-03-C		1.2*	cv=	20%
S240215-04-A	A-I-Post7-P, Radar, Pitfall	ND	$Mean_3$=	ND
S240215-04-B		ND	sd=	------
S240215-04-C		ND	cv=	------
S240215-05-A	B-I-Post7-P, Brackish, Pitfall	ND	$Mean_3$=	ND
S240215-05-B		ND	sd=	------
S240215-05-C		ND	cv=	------
S240215-06-A	C-I-Post7-P, RustyBucket, Pitfall	ND	$Mean_3$=	ND
S240215-06-B		ND	sd=	------
S240215-06-C		ND	cv=	------

*-Value is below the method quantitation limit of 3.97 ng/g

QC Results:

ID	Theoretical Brodifacoum Concentration (ng/g)	Observed Brodifacoum Concentration (ng/g)	% Recovery
QC-01	Control	ND	N/A
QC-02	Control	ND	N/A
QC-03	13.3	12.3	92.5
QC-04	13.4	13.6	102
QC-05	507	509	100
QC-06	513	514	100
QC-07	2140	2160	101
QC-08	2270	2250	99.1

ND = Not Detected.
