# Supplementary material for: Anticoagulant residues associated with an attempted rodent eradication from a subtropical coral atoll
Source: PLoS One. 2026 Mar 23;21(3):e0344972. doi: 10.1371/journal.pone.0344972 (PMC13008109; doi:10.1371/journal.pone.0344972)
Supplement: S1 Appendix — (ZIP) [file pone.0344972.s001.zip › Supporting Information S1/24-019-4 Pre-App Midway Island Reptiles Report.pdf]

To: Carmen Antaky
Biologist
NWRC Hawai'i Field Station

Subject: Determination of brodifacoum in reptile matrices from Midway Island, Post 2 (QA-3404)

Methods: 188A "Determination of Multiple Rodenticide Residues in Avian Liver by dSPE and LC-MS/MS" -Non-GLP

Analysis Dates: 05/16/24

Notebook References: AC169, pp.116, 119, 130, 134
QC35, p.68

Analyst: Ben Abbo

Sample Description:

Three reptile samples were submitted on 01/11/24. See sample descriptions on p.3.

Additional Comments:

- Three replicates of each sample were analyzed. The mean, standard deviation, and coefficient of variance are reported.
- Replicate sample S240111-021-A had a small amount of brodifacoum. Given that the other replicates for the sample did not have detectable levels of brodifacoum, this is likely a carry-over from the previous standard injection and not an indication of brodifacoum in the sample. The overall mean value for the samples was determined by assigning a value of ½ the value of the detection limit (0.50 ng/g) to the replicates with no detected brodifacoum for calculations. The mean for the sample was below the detection limit and is reported as Not Detected.
- Control gecko (S220801-47) was used as the matrix for QC samples.

Method Limit of Detection/Quantitation (MLOD/MLOQ) Values:

Method detection and quantitation limits were determined by comparing the noise at the analyte retention in three unfortified control gecko samples to the peak height of brodifacoum in three control gecko samples fortified to ~15 ng/g brodifacoum. The detection limit was determined to be 3X the noise and the quantitation limit was determined to be 10X the noise found in the unfortified samples.

Method Limit of Detection (MLOD)

Matrix	Detection Limit
Reptile	1.0 ng/g

Method Limit of Quantitation (MLOQ)

Matrix	Quantitation Limit
Reptile	3.46 ng/g

Results:

Sample ID	Sample Description	Brodifacoum Conc (ng/g)	Descriptive Statistics	
S240111-021-A	Geckos, A-I-PreApp-Ge, House gecko,	1.7*	$Mean_3$=	ND
S240111-021-B	Clipper, 6/21/2023	ND	sd=	-
S240111-021-C		ND	cv=	-
S240111-022-A	Geckos, B-I-PreApp-Ge, House gecko,	ND	$Mean_3$=	ND
S240111-022-B	Gym/Store, 6/21/2023	ND	sd=	-
S240111-022-C		ND	cv=	-
S240111-023-A	Geckos, C-I-PreApp-Ge, House gecko,	ND	$Mean_3$=	ND
S240111-023-B	FWS Office, 6/21/2023	ND	sd=	-
S240111-023-C		ND	cv=	-

ND = Not Detected.
*-Sample is below the quantitation limit of 3.46 ng/g.

QC Results:

ID	Theoretical Brodifacoum Concentration (ng/g)	Observed Brodifacoum Concentration (ng/g)	% Recovery
QC-25	Control	ND	N/A
QC-26	Control	ND	N/A
QC-27	14.4	14.0	97.2
QC-28	13.6	11.8	86.8
QC-29	486	443	91.2
QC-30	541	500	92.4
QC-31	2090	2020	96.7
QC-32	2500	2360	94.4

ND = Not Detected.
