# Supplementary material for: Anticoagulant residues associated with an attempted rodent eradication from a subtropical coral atoll
Source: PLoS One. 2026 Mar 23;21(3):e0344972. doi: 10.1371/journal.pone.0344972 (PMC13008109; doi:10.1371/journal.pone.0344972)
Supplement: S1 Appendix — (ZIP) [file pone.0344972.s001.zip › Supporting Information S1/24-019-5 Pre-App Midway Island Avian Livers Report.pdf]

| Wildlife Services NWRC National Wildlife Research Center<br>Analytical Services Report | United States Department of Agriculture<br>Animal Plant Health Inspection Service<br>Wildlife Services<br>National Wildlife Research Center<br>Laboratory Support Services Unit | Invoice #: 24-019/5<br>Date: 06/17/24<br> |
|---|---|---|

To: Carmen Antaky
Biologist
NWRC Hawai'i Field Station

Subject: Determination of brodifacoum in avian livers matrices from Midway Island, Pre-application (QA-3404)

Methods: 188A "Determination of Multiple Rodenticide Residues in Avian Liver by dSPE and LC-MS/MS" -Non-GLP

Analysis Dates: 05/16/24

Notebook References: AC169, pp.116, 120, 131, 134
QC35, p.68

Analyst: Ben Abbo

---

**Sample Description:**

Four avian liver samples were submitted on 01/11/24. See sample descriptions on p.3.

---

**Additional Comments:**

- Three replicates of each sample were analyzed, unless there was insufficient sample to analyze three replicates. The mean, standard deviation, and coefficient of variance are reported.
- Control quail liver (S221018-03) was used as the matrix for QC samples.

**Method Limit of Detection/Quantitation (MLOD/MLOQ) Values:**

Method detection and quantitation limits were determined by comparing the noise at the analyte retention in three unfortified control quail liver samples to the peak height of brodifacoum in three control quail liver samples fortified to ~15 ng/g brodifacoum. The detection limit was determined to be 3X the noise and the quantitation limit was determined to be 10X the noise found in the unfortified samples.

Method Limit of Detection (MLOD)

| Matrix | Detection Limit |
|---|---|
| Avian Liver | 1.3 ng/g |

Method Limit of Quantitation (MLOQ)

| Matrix | Quantitation Limit |
|---|---|
| Avian Liver | 4.26 ng/g |

**Results:**

| Sample ID | Sample Description | Brodifacoum Conc (ng/g) | Descriptive Statistics | |
|---|---|---|---|---|
| S240111-039-A | Sentinel, A-I-PreApp-My, Myna. Died in captive care, FWS Office, 6/10/2023 | ND | $Mean_2$= | ND |
| S240111-039-B | | ND | sd= | - |
| S240111-039-C | | ND | cv= | - |
| S240111-040-A | Sentinel, A-I-PreApp-Ca, Canary. Died in captive care, FWS Office, 6/30/2023 | ND | $Mean_3$= | ND |
| S240111-040-B | | No Sample | sd= | - |
| S240111-040-C | | No Sample | cv= | - |
| S240111-041-A | Seabird, A-I-PreApp-Se, Bonin petrel, Old taxiway, 6/22/2023 | ND | $Mean_3$= | ND |
| S240111-041-B | | ND | sd= | - |
| S240111-041-C | | ND | cv= | - |
| S240111-042-A | Seabird, A-II-PreApp-Se, LAAL liver, FWS Office, 6/28/2023 | ND | $Mean_3$= | ND |
| S240111-042-B | | No Sample | sd= | - |
| S240111-042-C | | No Sample | cv= | - |

ND = Not Detected.
INS-Insufficient Sample

**QC Results:**

| ID | Theoretical Brodifacoum Concentration (ng/g) | Observed Brodifacoum Concentration (ng/g) | % Recovery |
|---|---|---|---|
| QC-33 | Control | ND | N/A |
| QC-34 | Control | ND | N/A |
| QC-35 | 12.9 | 12.9 | 100 |
| QC-36 | 14.9 | 14.8 | 99.3 |
| QC-37 | 517 | 471 | 91.1 |
| QC-38 | 551 | 514 | 93.3 |
| QC-39 | 2460 | 2350 | 95.5 |
| QC-40 | 2150 | 2070 | 96.3 |

ND = Not Detected.
