# Supplementary material for: Anticoagulant residues associated with an attempted rodent eradication from a subtropical coral atoll
Source: PLoS One. 2026 Mar 23;21(3):e0344972. doi: 10.1371/journal.pone.0344972 (PMC13008109; doi:10.1371/journal.pone.0344972)
Supplement: S1 Appendix — (ZIP) [file pone.0344972.s001.zip › Supporting Information S1/24-019-6 Pre-App Midway Island Plants Report.pdf]

To: Carmen Antaky
Biologist
NWRC Hawai'i Field Station

Subject: Determination of brodifacoum in plant matrices from Midway Island, Pre-application (QA-3404)

Methods: 188A "Determination of Multiple Rodenticide Residues in Avian Liver by dSPE and LC-MS/MS" -Non-GLP

Analysis Dates: 05/16/24

Notebook References: AC169, pp.116, 119, 132, 134
QC35, p.68

Analyst: Ben Abbo

Sample Description:

Three plant samples were submitted on 01/11/24. See sample descriptions on p.3.

Additional Comments:

- Three replicates of each sample were analyzed, unless there was insufficient sample to analyze three replicates. The mean, standard deviation, and coefficient of variance are reported.
- Control lime (S221013-01) was used as the matrix for QC samples.

Method Limit of Detection/Quantitation (MLOD/MLOQ) Values:

Method detection and quantitation limits were determined by comparing the noise at the analyte retention in three unfortified control lime samples to the peak height of brodifacoum in three control lime samples fortified to ~15 ng/g brodifacoum. The detection limit was determined to be 3X the noise and the quantitation limit was determined to be 10X the noise found in the unfortified samples.

Method Limit of Detection (MLOD)

Matrix	Detection Limit
Plants	1.1 ng/g

Method Limit of Quantitation (MLOQ)

Matrix	Quantitation Limit
Plants	3.72 ng/g

Results:

Sample ID	Sample Description	Brodifacoum Conc (ng/g)	Descriptive Statistics	
S240111-046-A	Plants, A-I-PreApp-Pl, Papaya , Community Garden A, 6/22/2023	ND	$Mean_3$=	ND
S240111-046-B		ND	sd=	-
S240111-046-C		ND	cv=	-
S240111-047-A	Plants, B-I-PreApp-Pl, Kaffir lime leaves, Community Garden B, 6/22/2023	ND	$Mean_3$=	ND
S240111-047-B		ND	sd=	-
S240111-047-C		ND	cv=	-
S240111-048-A	Plants, C-I-PreApp-Pl, Pesian lime, Orchard, 6/22/2023	ND	$Mean_3$=	ND
S240111-048-B		ND	sd=	-
S240111-048-C		ND	cv=	-

ND = Not Detected.
*-Value is below the method quantitation limit of 3.72 ng/g

QC Results:

ID	Theoretical Brodifacoum Concentration (ng/g)	Observed Brodifacoum Concentration (ng/g)	% Recovery
QC-41	Control	ND	N/A
QC-42	Control	ND	N/A
QC-43	13.8	13.0	94.2
QC-44	14.5	13.4	92.4
QC-45	509	475	93.3
QC-46	512	473	92.4
QC-47	2430	2340	96.3
QC-48	2310	2220	96.1

ND = Not Detected.
