# Supplementary material for: Anticoagulant residues associated with an attempted rodent eradication from a subtropical coral atoll
Source: PLoS One. 2026 Mar 23;21(3):e0344972. doi: 10.1371/journal.pone.0344972 (PMC13008109; doi:10.1371/journal.pone.0344972)
Supplement: S1 Appendix — (ZIP) [file pone.0344972.s001.zip › Supporting Information S1/24-019-8 Pre-App Midway Island Waters Report.pdf]

Wildlife Services **NWRC** National Wildlife Research Center<br>Analytical Services Report	United States Department of Agriculture<br>Animal Plant Health Inspection Service<br>Wildlife Services<br>National Wildlife Research Center<br>Laboratory Support Services Unit	Invoice #: 24-019/8<br>Date: 06/17/24<br>Page: 1 of 5

To: Carmen Antaky
Biologist
NWRC Hawai'i Field Station

Subject: Determination of brodifacoum in Waters from Midway Island, Pre-application (QA-3404)

Methods: "Determination of Brodifacoum Residues in Water"-Non-GLP

Analysis Dates: 04/24/24

Notebook References: AC169, pp.20-21, 116-118
QC35, p.68

Analyst: Ben Abbo

Sample Description:

Twelve samples of water from Midway Atoll were submitted on 01/11/24. See sample descriptions on pp.3-4.

Additional Comments:

- Two replicates of each sample were analyzed.
- Cache la Poudre River water from Laporte, Colorado (S230825-03) was used as the matrix for QC samples for fresh water.
- Synthetic sea water (NWRC ID# 7262) was used as the matrix for QC samples for sea water.

Contact the author for further details on QA/QC certification at Carmen.Antaky@usda.gov

Analyst	Date	QC Specialist	Date	Reviewer	Date

Sample Preparation and Extraction:

Water Extraction Procedure:

1. Add 60 mL of water sample to a 125-mL separatory funnel.
2. Add 0.020 mL surrogate to all and 0.040 mL of acetonitrile or 75X brodifacoum stock as indicated.
3. Add 20 mL chloroform.
4. Add ~8.5g sodium chloride.
5. Add 10 mL 1M hydrochloric acid.
6. Cap and shake for 8-10s, let set 1 minute. Repeat 2X.
7. Dispense chloroform phase into 25-mL glass tube taking care to not transfer any water phase.
8. Remove solvent in a 60°C N-Evap with a gentle flow of nitrogen gas.
9. Add 0.300 mL acetonitrile, vortex thoroughly, wetting as much of the inside surface of the tube as possible.
10. Add 1.200 mL pH 9.5 20-mM ammonium acetate buffer and vortex thoroughly.
11. Transfer the sample to an autosampler vial and assay by LC-MS/MS.

Method Limit of Detection/Quantitation (MLOD/MLOQ) Values:

Method detection and quantitation limits were determined by comparing the noise at the analyte retention in two unfortified control water samples to the peak height of brodifacoum in two control water samples fortified to 0.0219 ng/mL brodifacoum. The detection limit was determined to be 3X the noise and the quantitation limit was determined to be 10X the noise found in the unfortified samples.

Method Limit of Detection (MLOD)

Matrix	Detection Limit
Fresh water	0.0028 ng/mL
Sea water	0.0032 ng/mL

Method Limit of Quantitation (MLOQ)

Matrix	Quantitation Limit
Fresh water	0.00938 ng/mL
Sea water	0.0107 ng/mL

Results:

Fresh Water

Sample ID	Sample Description	Brodifacoum (ng/g)	Descriptive Statistics	
S240111-027-A	Drinking Water, A-I-PreApp-DW, FWS	ND	Avg_2=	ND
S240111-027-B	Office, 6/27/2023	ND	sd=	-
			cv=	-
S240111-028-A	Drinking Water, B-I-PreApp-DW, Charlie	ND	Avg_2=	ND
S240111-028-B	Barracks, 6/27/2023	ND	sd=	-
			cv=	-
S240111-033-A	Fresh Water, A-I-PreApp-Fr, Radar,	ND	Avg_2=	ND
S240111-033-B	6/27/2023	ND	sd=	-
			cv=	-
S240111-034-A	Fresh Water, B-I-PreApp-Fr, Brackish,	ND	Avg_2=	ND
S240111-034-B	6/27/2023	ND	sd=	-
			cv=	-
S240111-035-A	Fresh Water, C-I-PreApp-Fr, R2,	ND	Avg_2=	ND
S240111-035-B	6/21/2023	ND	sd=	-
			cv=	-
S240111-036-A	Fresh Water, D-I-PreApp-Fr, Office	ND	Avg_2=	ND
S240111-036-B	Guzzler, 6/27/2023	ND	sd=	-
			cv=	-
S240111-037-A	Drainage Water, A-I-PreApp-WC,	ND	Avg_2=	ND
S240111-037-B	catchment pond, Catchment, 6/22/2023	ND	sd=	-
			cv=	-
S240111-038-A	Drainage Water, B-I-PreApp-WC,	ND	Avg_2=	ND
S240111-038-B	Bowling Alley, 6/22/2023	ND	sd=	-
			cv=	-

ND-Not Detected
*-Sample is below the quantitation limit of 0.00938 ng/mL

Sea Water

Sample ID	Sample Description	Brodifacoum (ng/g)	Descriptive Statistics	
S240111-029-A	Ocean Water, A-I-PreApp-Oc, CargoPier,	ND	Avg_2=	ND
S240111-029-B	6/20/2023	ND	sd=	-
			cv=	-
S240111-030-A	Ocean Water, B-I-PreApp-Oc, Hale Honu,	ND	Avg_2=	ND
S240111-030-B	6/20/2023	ND	sd=	-
			cv=	-
S240111-031-A	Ocean Water, C-I-PreApp-Oc, RustyBucket,	ND	Avg_2=	ND
S240111-031-B	6/20/2023	ND	sd=	-
			cv=	-
S240111-032-A	Ocean Water, D-I-PreApp-Oc, Harbor,	ND	Avg_2=	ND
S240111-032-B	6/21/2023	ND	sd=	-
			cv=	-

ND-Not Detected

QC Results:

Fresh Water:

ID	Theoretical Brodifacoum Concentration (ng/mL)	Observed Brodifacoum Concentration (ng/mL)	% Recovery
QC-01	Control	ND	N/A
QC-02	Control	ND	N/A
QC-03	0.0219	0.0234	107
QC-04	0.0219	0.0226	103
QC-05	1.77	1.81	102
QC-06	1.77	1.80	102
QC-07	5.32	5.32	100
QC-08	5.32	5.35	101

ND-Not Detected

Sea Water:

ID	Theoretical Brodifacoum Concentration (ng/mL)	Observed Brodifacoum Concentration (ng/mL)	% Recovery
QC-09	Control	ND	N/A
QC-10	Control	ND	N/A
QC-11	0.0219	0.0214	97.7
QC-12	0.0219	0.0219	100
QC-13	1.77	1.76	99.4
QC-14	1.77	1.78	101
QC-15	5.32	5.36	101
QC-16	5.32	5.46	103

ND-Not Detected
