# Supplementary material for: Anticoagulant residues associated with an attempted rodent eradication from a subtropical coral atoll
Source: PLoS One. 2026 Mar 23;21(3):e0344972. doi: 10.1371/journal.pone.0344972 (PMC13008109; doi:10.1371/journal.pone.0344972)
Supplement: S1 Appendix — (ZIP) [file pone.0344972.s001.zip › Supporting Information S1/24-020 Post 2 Midway Island Avian Livers Report.pdf]

To: Carmen Antaky
Biologist
NWRC Hawai'i Field Station

Subject: Determination of brodifacoum in avian livers matrices from Midway Island, Post 2 (QA-3404)

Methods: 188A "Determination of Multiple Rodenticide Residues in Avian Liver by dSPE and LC-MS/MS" -Non-GLP

Analysis Dates: 04/04/24

Notebook References: AC169, pp.20-21, 101-102, 106, 113, 115
QC35, p.68

Analyst: Ben Abbo

Sample Description:

Three avian liver samples were submitted on 01/11/24. See sample descriptions on p.3.

Additional Comments:

- Three replicates of each sample were analyzed except for sample S240111-109. There was only sufficient sample to analyze two replicates of this sample. The mean, standard deviation, and coefficient of variance are reported.
- Control quail liver (S221018-03) was used as the matrix for QC samples.

Method Limit of Detection/Quantitation (MLOD/MLOQ) Values:

Method detection and quantitation limits were determined by comparing the noise at the analyte retention in three unfortified control quail liver samples to the peak height of brodifacoum in three control quail liver samples fortified to ~15 ng/g brodifacoum. The detection limit was determined to be 3X the noise and the quantitation limit was determined to be 10X the noise found in the unfortified samples.

Method Limit of Detection (MLOD)

Matrix	Detection Limit
Avian Liver	1.3 ng/g

Method Limit of Quantitation (MLOQ)

Matrix	Quantitation Limit
Avian Liver	4.26 ng/g

Results:

Sample ID	Sample Description	Brodifacoum Conc (ng/g)	Descriptive Statistics	
S240111-109-A	Duck, B-I-Post2-Du, LADU liver 23-143 ,	2110	$Mean_2$=	2050
S240111-109-B	Eastern (monument seep), 7/27/2023	1980	sd=	92
S240111-109-C		No Sample	cv=	4.5%
S240111-110-A	Sentinel, A-I-Post2-Egret, Egret, Eastern	914	$Mean_3$=	937
S240111-110-B	(EIMA guzzler), 7/28/2023	952	sd=	20
S240111-110-C		946	cv=	2.1%
S240111-111-A	Seabird, A-I-Post2-Se, LAAL liver,	ND	$Mean_3$=	ND
S240111-111-B	Bowling Alley, 7/28/2023	ND	sd=	-
S240111-111-C		ND	cv=	-

ND = Not Detected.
INS-Insufficient Sample

QC Results:

ID	Theoretical Brodifacoum Concentration (ng/g)	Observed Brodifacoum Concentration (ng/g)	% Recovery
QC-33	Control	ND	N/A
QC-34	Control	ND	N/A
QC-35	14.0	11.9	85.0
QC-36	15.3	13.6	88.9
QC-37	553	565	102
QC-38	534	537	101
QC-39	2280	2250	98.7
QC-40	2070	1990	96.1

ND = Not Detected.
