# Supplementary material for: Anticoagulant residues associated with an attempted rodent eradication from a subtropical coral atoll
Source: PLoS One. 2026 Mar 23;21(3):e0344972. doi: 10.1371/journal.pone.0344972 (PMC13008109; doi:10.1371/journal.pone.0344972)
Supplement: S1 Appendix — (ZIP) [file pone.0344972.s001.zip › Supporting Information S1/24-020 Post 2 Midway Island Fish Report.pdf]

To:	Carmen Antaky<br>Biologist<br>NWRC Hawai'i Field Station
Subject:	Determination of brodifacoum in fish matrices from Midway Island, Post 2 (QA-3404)
Methods:	188A "Determination of Multiple Rodenticide Residues in Avian Liver by dSPE and LC-MS/MS" -Non-GLP
Analysis Dates:	04/03/24
Notebook References:	AC169, pp.20-21, 101-102, 105, 109-110<br>QC35, p.68
Analyst:	Ben Abbo

Sample Description:

Eighteen fish samples were submitted on 01/11/24. See sample descriptions on pp.3-4.

Additional Comments:

- Three replicates of each sample were analyzed. The mean, standard deviation, and coefficient of variance are reported.
- Control sea bass (S220906-01) was used as the matrix for QC samples.

Method Limit of Detection/Quantitation (MLOD/MLOQ) Values:

Method detection and quantitation limits were determined by comparing the noise at the analyte retention in three unfortified control sea bass samples to the peak height of brodifacoum in three control sea bass samples fortified to ~15 ng/g brodifacoum. The detection limit was determined to be 3X the noise and the quantitation limit was determined to be 10X the noise found in the unfortified samples.

Method Limit of Detection (MLOD)

Matrix	Detection Limit
Fish	1.2 ng/g

Method Limit of Quantitation (MLOQ)

Matrix	Quantitation Limit
Fish	4.06 ng/g

Results:

Sample ID	Sample Description	Brodifacoum Conc (ng/g)	Descriptive Statistics	
S240111-069-A	Mosquito Fish , B-I-Post2-M, Gambusia,	3.2*	$Mean_3$=	2.4*
S240111-069-B	Catchment, 7/26/2023	2.0*	sd=	0.69
S240111-069-C		2.0*	cv=	29%
S240111-070-A	Mosquito Fish , B-II-Post2-M, Gambusia,	ND	$Mean_3$=	ND
S240111-070-B	Catchment, 7/26/2023	ND	sd=	-
S240111-070-C		ND	cv=	-
S240111-071-A	Mosquito Fish , C-I-Post2-M, Gambusia,	84.3	$Mean_3$=	85.8
S240111-071-B	Drainage Pond, 7/26/2023	85.9	sd=	1.5
S240111-071-C		87.2	cv=	1.7%
S240111-072-A	Mosquito Fish , C-II-Post2-M, Gambusia,	146	Value=	146
S240111-072-B	Drainage Pond, 7/26/2023	No Sample	sd=	-
S240111-072-C		No Sample	cv=	-
S240111-073-A	Bait Fish, A-I-Post2-Bait, Flagtail,	37.1	$Mean_3$=	44.9
S240111-073-B	CargoPier, 7/28/2023	50.7	sd=	7.0
S240111-073-C		46.8	cv=	16%
S240111-074-A	Bait Fish, A-II-Post2-Bait, Flagtail,	8.27	$Mean_3$=	9.18
S240111-074-B	CargoPier, 7/28/2023	9.27	sd=	0.87
S240111-074-C		10.0	cv=	9.5%
S240111-075-A	Bait Fish, B-I-Post2-Bait, Flagtail, Hale	99.6	$Mean_3$=	82.2
S240111-075-B	Honu, 7/28/2023	78.0	sd=	16
S240111-075-C		69.0	cv=	19%
S240111-076-A	Bait Fish, B-II-Post2-Bait, Flagtail, Hale	58.0	$Mean_3$=	62.8
S240111-076-B	Honu, 7/28/2023	65.3	sd=	4.1
S240111-076-C		65.0	cv=	6.5%
S240111-077-A	Bait Fish, C-I-Post2-Bait, Flagtail,	43.0	$Mean_3$=	44.3
S240111-077-B	RustyBucket, 7/27/2023	44.6	sd=	1.1
S240111-077-C		45.2	cv=	2.5%
S240111-078-A	Bait Fish, C-II-Post2-Bait, Flagtail,	37.1	$Mean_3$=	49.3
S240111-078-B	RustyBucket, 7/27/2023	62.8	sd=	13
S240111-078-C		47.9	cv=	26%
S240111-079-A	Reef Fish, A-I-Post2-Reef, Moi, CargoPier,	ND	$Mean_3$=	ND
S240111-079-B	7/28/2023	ND	sd=	-
S240111-079-C		ND	cv=	-
S240111-080-A	Reef Fish, A-I-Post2-Reef, Moi, CargoPier,	ND	$Mean_3$=	ND
S240111-080-B	7/28/2023	ND	sd=	-
S240111-080-C		ND	cv=	-

ND = Not Detected.
*-Value is below the method quantitation limit of 4.06 ng/g

Results:

Sample ID	Sample Description	Brodifacoum Conc (ng/g)	Descriptive Statistics	
S240111-081-A	Reef Fish, B-I-Post2-Reef, Goatfish, Hale Honu, 7/28/2023	ND	$Mean_3$=	ND
S240111-081-B		ND	sd=	-
S240111-081-C		ND	cv=	-
S240111-082-A	Reef Fish, B-II-Post2-Reef, Goatfish, Hale Honu, 7/28/2023	ND	$Mean_3$=	ND
S240111-082-B		ND	sd=	-
S240111-082-C		ND	cv=	-
S240111-083-A	Reef Fish, C-I-Post2-Reef, Moi, RustyBucket, 7/27/2023	ND	$Mean_3$=	ND
S240111-083-B		ND	sd=	-
S240111-083-C		ND	cv=	-
S240111-084-A	Reef Fish, C-II-Post2-Reef, Moi, RustyBucket, 7/27/2023	2.7*	$Mean_3$=	1.7
S240111-084-B		1.7*	sd=	1.1
S240111-084-C		ND	cv=	66%
S240111-116-A	Fish Carcass, A-II-Post2-Fc, Mosquitofish , R2, 7/21/2023	37.3	$Mean_3$=	36.9
S240111-116-B		37.1	sd=	0.59
S240111-116-C		36.2	cv=	1.6%
S240111-117-A	Fish Carcass, A-IV-Post2-Fc, Mosquitofish , Drainage Pond, 7/22/2023	107	$Mean_3$=	105
S240111-117-B		106	sd=	2.6
S240111-117-C		102	cv=	2.5%

ND = Not Detected.
When a replicate has a value of ND, ½ of the detection limit is used as the value for that replicate when calculating the mean value for the sample.
*-Value is below the method quantitation limit of 4.06 ng/g

QC Results:

ID	Theoretical Brodifacoum Concentration (ng/g)	Observed Brodifacoum Concentration (ng/g)	% Recovery
QC-09	Control	ND	N/A
QC-10	Control	ND	N/A
QC-11	13.7	13.8	101
QC-12	15.6	13.0	83.3
QC-13	479	460	96.0
QC-14	574	570	99.3
QC-15	2010	1950	97.0
QC-16	2220	2160	97.3

ND = Not Detected.
