# Supplementary material for: Anticoagulant residues associated with an attempted rodent eradication from a subtropical coral atoll
Source: PLoS One. 2026 Mar 23;21(3):e0344972. doi: 10.1371/journal.pone.0344972 (PMC13008109; doi:10.1371/journal.pone.0344972)
Supplement: S1 Appendix — (ZIP) [file pone.0344972.s001.zip › Supporting Information S1/24-020 Post 2 Midway Island Invertebrates Report.pdf]

To: Carmen Antaky
Biologist
NWRC Hawai'i Field Station

Subject: Determination of brodifacoum in invertebrate matrices from Midway Island, Post 2 (QA-3404)

Methods: 188A "Determination of Multiple Rodenticide Residues in Avian Liver by dSPE and LC-MS/MS" -Non-GLP

Analysis Dates: 04/02/24

Notebook References: AC169, pp.20-21, 101-102, 104, 107-108
QC35, p.68

Analyst: Ben Abbo

Sample Description:

Twenty invertebrate samples were submitted on 01/11/24. See sample descriptions on pp.3-4.

Additional Comments:

- Three replicates of each sample were analyzed, unless there was insufficient sample to analyze three replicates. The mean, standard deviation, and coefficient of variance are reported.
- Control crickets (S221018-01) were used as the matrix for QC samples.

Method Limit of Detection/Quantitation (MLOD/MLOQ) Values:

Method detection and quantitation limits were determined by comparing the noise at the analyte retention in three unfortified control cricket samples to the peak height of brodifacoum in three control cricket samples fortified to ~15 ng/g brodifacoum. The detection limit was determined to be 3X the noise and the quantitation limit was determined to be 10X the noise found in the unfortified samples.

Method Limit of Detection (MLOD)

Matrix	Detection Limit
Invertebrates	1.2 ng/g

Method Limit of Quantitation (MLOQ)

Matrix	Quantitation Limit
Invertebrates	3.97 ng/g

Results:

Sample ID	Sample Description	Brodifacoum Conc (ng/g)	Descriptive Statistics	
S240111-049-A	Grubs, A-I-Post2-G, Emerald beetle, Radar,	18.2	$Mean_3$=	16.8
S240111-049-B	8/3/2023	16.6	sd=	1.3
S240111-049-C		15.6	cv=	7.7%
S240111-050-A	Grubs, A-II-Post2-G, Emerald beetle, Radar,	17.1	$Mean_3$=	16.4
S240111-050-B	8/3/2023	15.3	sd=	0.99
S240111-050-C		16.9	cv=	6.0%
S240111-051-A	Grubs, B-I-Post2-G, Emerald beetle,	8.40	$Mean_3$=	8.62
S240111-051-B	Brackish, 8/3/2023	8.95	sd=	0.29
S240111-051-C		8.50	cv=	3.4%
S240111-052-A	Grubs, B-II-Post2-G, Emerald beetle,	11.7	$Mean_3$=	11.2
S240111-052-B	Brackish, 8/3/2023	10.6	sd=	0.55
S240111-052-C		11.2	cv=	4.9%
S240111-053-A	Grubs, C-I-Post2-G, Emerald beetle,	7.51	$Mean_3$=	7.57
S240111-053-B	RustyBucket, 8/3/2023	7.83	sd=	0.24
S240111-053-C		7.37	cv=	3.2%
S240111-054-A	Grubs, C-II-Post2-G, Emerald beetle,	4.15	$Mean_3$=	3.7*
S240111-054-B	RustyBucket, 8/3/2023	3.4*	sd=	0.41
S240111-054-C		3.5*	cv=	11%
S240111-055-A	Roaches, A-I-Post2-R, Blattodea, Radar,	ND	$Mean_3$=	ND
S240111-055-B	7/27/2023	ND	sd=	-
S240111-055-C		ND	cv=	-
S240111-056-A	Roaches, A-II-Post2-R, Blattodea, Radar,	ND	$Mean_3$=	ND
S240111-056-B	7/27/2023	ND	sd=	-
S240111-056-C		No Sample	cv=	-
S240111-057-A	Roaches, B-I-Post2-R, Blattodea, Brackish,	413	$Mean_3$=	400
S240111-057-B	7/27/2023	383	sd=	16
S240111-057-C		405	cv=	4.0%
S240111-058-A	Roaches, B-II-Post2-R, Blattodea, Brackish,	259	$Mean_3$=	256
S240111-058-B	7/27/2023	253	sd=	4.2
S240111-058-C		No Sample	cv=	1.6%
S240111-059-A	Roaches, C-I-Post2-R, Blattodea,	167	$Mean_3$=	173
S240111-059-B	RustyBucket, 7/27/2023	172	sd=	6.0
S240111-059-C		179	cv=	3.5%
S240111-060-A	Roaches, C-II-Post2-R, Blattodea,	123	$Mean_3$=	117
S240111-060-B	RustyBucket, 7/27/2023	111	sd=	8.5
S240111-060-C		No Sample	cv=	7.3%

ND = Not Detected.

*-Value is below the method quantitation limit of 3.97 ng/g

Results:

Sample ID	Sample Description	Brodifacoum Conc (ng/g)	Descriptive Statistics	
S240111-061-A	Roaches, D-I-Post2-R, Blattodea, Ballfield,	146	$Mean_3$=	145
S240111-061-B	7/27/2023	140	sd=	5.0
S240111-061-C		150	cv=	3.4%
S240111-062-A	Roaches, D-II-Post2-R, Blattodea, Ballfield,	70.6	$Mean_3$=	71.6
S240111-062-B	7/27/2023	72.0	sd=	0.91
S240111-062-C		72.3	cv=	1.3%
S240111-063-A	Pitfall, A-I-Post2-Pitfall, Pooled, Radar,	285	$Mean_3$=	257
S240111-063-B	8/1/2023	231	sd=	27
S240111-063-C		254	cv=	11%
S240111-064-A	Pitfall, A-II-Post2-Pitfall, Pooled, Radar,	28.8	$Mean_3$=	29.7
S240111-064-B	8/1/2023	29.2	sd=	1.2
S240111-064-C		31.0	cv=	4.0%
S240111-065-A	Pitfall, B-I-Post2-Pitfall, Pooled, Brackish,	52.1	Value=	52.1
S240111-065-B	8/2/2023	No Sample	sd=	-
S240111-065-C		No Sample	cv=	-
S240111-066-A	Pitfall, B-II-Post2-Pitfall, Pooled, Brackish,	920	Value=	920
S240111-066-B	8/2/2023	No Sample	sd=	-
S240111-066-C		No Sample	cv=	-
S240111-067-A	Pitfall, C-I-Post2-Pitfall, Pooled,	339	Value=	339
S240111-067-B	RustyBucket, 8/2/2023	No Sample	sd=	-
S240111-067-C		No Sample	cv=	-
S240111-068-A	Pitfall, C-II-Post2-Pitfall, Pooled,	58.8	Value=	58.8
S240111-068-B	RustyBucket, 8/2/2023	No Sample	sd=	-
S240111-068-C		No Sample	cv=	-

ND = Not Detected.
*-Value is below the method quantitation limit of 3.97 ng/g
INS-Insufficient Sample

QC Results:

ID	Theoretical Brodifacoum Concentration (ng/g)	Observed Brodifacoum Concentration (ng/g)	% Recovery
QC-01	Control	ND	N/A
QC-02	Control	ND	N/A
QC-03	14.5	13.2	91.0
QC-04	12.5	10.9	87.2
QC-05	462	453	98.1
QC-06	554	561	101
QC-07	2420	2410	99.6
QC-08	2470	2460	99.6

ND = Not Detected.
