# Supplementary material for: Anticoagulant residues associated with an attempted rodent eradication from a subtropical coral atoll
Source: PLoS One. 2026 Mar 23;21(3):e0344972. doi: 10.1371/journal.pone.0344972 (PMC13008109; doi:10.1371/journal.pone.0344972)
Supplement: S1 Appendix — (ZIP) [file pone.0344972.s001.zip › Supporting Information S1/24-020 Post 2 Midway Island Reptiles Report.pdf]

To: Carmen Antaky
Biologist
NWRC Hawai'i Field Station

Subject: Determination of brodifacoum in reptile matrices from Midway Island, Post 2 (QA-3404)

Methods: 188A "Determination of Multiple Rodenticide Residues in Avian Liver by dSPE and LC-MS/MS" -Non-GLP

Analysis Dates: 04/04/24

Notebook References: AC169, pp.20-21, 101-102, 106, 112, 115
QC35, p.68

Analyst: Ben Abbo

Sample Description:

Six reptile samples were submitted on 01/11/24. See sample descriptions on p.3.

Additional Comments:

- Three replicates of each sample were analyzed. The mean, standard deviation, and coefficient of variance are reported.
- Control gecko (S220801-47) was used as the matrix for QC samples.

Method Limit of Detection/Quantitation (MLOD/MLOQ) Values:

Method detection and quantitation limits were determined by comparing the noise at the analyte retention in three unfortified control gecko samples to the peak height of brodifacoum in three control gecko samples fortified to ~15 ng/g brodifacoum. The detection limit was determined to be 3X the noise and the quantitation limit was determined to be 10X the noise found in the unfortified samples.

Method Limit of Detection (MLOD)

Matrix	Detection Limit
Reptile	2.0 ng/g

Method Limit of Quantitation (MLOQ)

Matrix	Quantitation Limit
Reptile	6.59 ng/g

Results:

Sample ID	Sample Description	Brodifacoum Conc (ng/g)	Descriptive Statistics	
240111-091-A	Geckos, A-I-Post2-Ge, Mourning gecko,	32.6	$Mean_3$=	31.2
240111-091-B	Clipper, 8/3/2023	31.0	sd=	1.3
240111-091-C		30.0	cv=	4.2%
240111-092-A	Geckos, A-II-Post2-Ge, Mourning gecko,	27.3	$Mean_3$=	27.3
240111-092-B	Clipper, 8/3/2023	27.9	sd=	0.60
240111-092-C		26.7	cv=	2.2%
240111-093-A	Geckos, B-I-Post2-Ge, Indo-pacific gecko,	71.4	$Mean_3$=	72.9
240111-093-B	Gym/Store, 8/3/2023	72.9	sd=	1.5
240111-093-C		74.4	cv=	2.1%
240111-094-A	Geckos, B-II-Post2-Ge, Mourning gecko,	128	$Mean_3$=	116
240111-094-B	Gym/Store, 8/3/2023	127	sd=	20
240111-094-C		93.7	cv=	17%
240111-095-A	Geckos, C-I-Post2-Ge, Indo-pacific gecko,	154	$Mean_3$=	151
240111-095-B	FWS Office, 8/3/2023	146	sd=	4.4
240111-095-C		153	cv=	2.9%
240111-096-A	Geckos, C-II-Post2-Ge, Mourning gecko,	31.8	$Mean_3$=	30.9
240111-096-B	FWS Office, 8/3/2023	31.2	sd=	1.0
240111-096-C		29.8	cv=	3.2%

ND = Not Detected.

QC Results:

ID	Theoretical Brodifacoum Concentration (ng/g)	Observed Brodifacoum Concentration (ng/g)	% Recovery
QC-25	Control	ND	N/A
QC-26	Control	ND	N/A
QC-27	14.5	14.2	97.9
QC-28	14.4	12.8	88.9
QC-29	472	472	100
QC-30	552	547	99.1
QC-31	2510	2500	99.6
QC-32	2120	2070	97.6

ND = Not Detected.
