# Supplementary material for: Anticoagulant residues associated with an attempted rodent eradication from a subtropical coral atoll
Source: PLoS One. 2026 Mar 23;21(3):e0344972. doi: 10.1371/journal.pone.0344972 (PMC13008109; doi:10.1371/journal.pone.0344972)
Supplement: S1 Appendix — (ZIP) [file pone.0344972.s001.zip › Supporting Information S1/24-020 Post 2 Midway Island Soils Report.pdf]

Wildlife Services **NWRC** National Wildlife Research Center<br>Analytical Services Report	United States Department of Agriculture<br>Animal Plant Health Inspection Service<br>Wildlife Services<br>National Wildlife Research Center<br>Laboratory Support Services Unit	Invoice #: 24-020/6<br>Date: 05/02/24<br>Page: 1 of 4

To:	Carmen Antaky<br>Biologist<br>NWRC Hawai'i Field Station
Subject:	Determination of brodifacoum in soil matrices from Midway Island, Post 2 (QA-3404)
Methods:	188A "Determination of Multiple Rodenticide Residues in Avian Liver by dSPE and LC-MS/MS" -Non-GLP
Analysis Dates:	04/04/24
Notebook References:	AC169, pp.20-21, 101-102, 106, 114-115<br>QC35, p.68
Analyst:	Ben Abbo, Keli-Jean Smith

Sample Description:

Seven soil samples were submitted on 01/11/24. See sample descriptions on p.3.

Additional Comments:

- Three replicates of each sample were analyzed. The mean, standard deviation, and coefficient of variance are reported.
- Control soil (S220801-61) was used as the matrix for QC samples.

Method Limit of Detection/Quantitation (MLOD/MLOQ) Values:

Method detection and quantitation limits were determined by comparing the noise at the analyte retention in three unfortified control soil samples to the peak height of brodifacoum in three control soil samples fortified to ~15 ng/g brodifacoum. The detection limit was determined to be 3X the noise and the quantitation limit was determined to be 10X the noise found in the unfortified samples.

Method Limit of Detection (MLOD)

Matrix	Detection Limit
Soil	1.3 ng/g

Method Limit of Quantitation (MLOQ)

Matrix	Quantitation Limit
Soil	4.29 ng/g

Results:

Sample ID	Sample Description	Brodifacoum Conc (ng/g)	Descriptive Statistics	
240111-097-A	Soil, A-I-Post2-S, Radar, 7/25/2023	ND	$Mean_3$=	ND
240111-097-B		ND	sd=	-
240111-097-C		ND	cv=	-
240111-098-A	Soil, B-I-Post2-S, Brackish, 7/25/2023	ND	$Mean_3$=	ND
240111-098-B		ND	sd=	-
240111-098-C		ND	cv=	-
240111-099-A	Soil, C-I-Post2-S, covered, Community Garden , 7/24/2023	ND	$Mean_3$=	ND
240111-099-B		ND	sd=	-
240111-099-C		ND	cv=	-
240111-112-A	Soil, A-I-Post2-SS, Parade Ground, 7/24/2023	ND	$Mean_3$=	ND
240111-112-B		ND	sd=	-
240111-112-C		ND	cv=	-
240111-113-A	Soil, B-I-Post2-SS, Orchard, 7/24/2023	ND	$Mean_3$=	ND
240111-113-B		ND	sd=	-
240111-113-C		ND	cv=	-
240111-114-A	Soil, C-I-Post2-SS, uncovered, Community Garden , 7/24/2023	ND	$Mean_3$=	ND
240111-114-B		ND	sd=	-
240111-114-C		ND	cv=	-
240111-115-A	Soil, D-I-Post2-SS, Ballfield, 7/24/2023	ND	$Mean_3$=	ND
240111-115-B		ND	sd=	-
240111-115-C		ND	cv=	-

ND = Not Detected.
*-Sample was below quantitation limit of 4.29 ng/g; result should be considered qualitative.

QC Results:

ID	Theoretical Brodifacoum Concentration (ng/g)	Observed Brodifacoum Concentration (ng/g)	% Recovery
QC-41	Control	ND	N/A
QC-42	Control	ND	N/A
QC-43	14.3	12.8	89.5
QC-44	15.6	14.0	89.7
QC-45	506	506	100
QC-46	536	524	97.8
QC-47	2450	2440	99.6
QC-48	2430	2400	98.8

ND = Not Detected.
