# Supplementary material for: Anticoagulant residues associated with an attempted rodent eradication from a subtropical coral atoll
Source: PLoS One. 2026 Mar 23;21(3):e0344972. doi: 10.1371/journal.pone.0344972 (PMC13008109; doi:10.1371/journal.pone.0344972)
Supplement: S1 Appendix — (ZIP) [file pone.0344972.s001.zip › Supporting Information S1/24-027-2 Post 8 Midway Island Fish Report.pdf]

Wildlife Services NWRC National Wildlife Research Center<br>Analytical Services Report	United States Department of Agriculture<br>Animal Plant Health Inspection Service<br>Wildlife Services<br>National Wildlife Research Center<br>Laboratory Support Services Unit	Invoice #: 24-027/2<br>Date: 06/17/24<br>Page: 1 of 3

To: Carmen Antaky
Biologist
NWRC Hawai'i Field Station

Subject: Determination of brodifacoum in fish matrices from Midway Island, Post 8 (QA-3404)

Methods: 188A "Determination of Multiple Rodenticide Residues in Avian Liver by dSPE and LC-MS/MS" -Non-GLP

Analysis Dates: 06/04/24

Notebook References: AC169, pp.116, 135-137
QC35, p.68

Analyst: Ben Abbo

Sample Description:

Two fish samples were submitted on 04/19/24. See sample descriptions on p.3.

Additional Comments:

- Three replicates of each sample were analyzed. The mean, standard deviation, and coefficient of variance are reported.
- Control sea bass (S220906-01) was used as the matrix for QC samples.

Method Limit of Detection/Quantitation (MLOD/MLOQ) Values:

Method detection and quantitation limits were determined by comparing the noise at the analyte retention in three unfortified control sea bass samples to the peak height of brodifacoum in three control sea bass samples fortified to ~15 ng/g brodifacoum. The detection limit was determined to be 3X the noise and the quantitation limit was determined to be 10X the noise found in the unfortified samples.

Method Limit of Detection (MLOD)

Matrix	Detection Limit
Fish	1.2 ng/g

Method Limit of Quantitation (MLOQ)

Matrix	Quantitation Limit
Fish	4.06 ng/g

Results:

Sample ID	Sample Description	Brodifacoum Conc (ng/g)	Descriptive Statistics	
S240419-07-A	Mosquito Fish, A-I-Post8-MOSQ,	16.1	$Mean_3$=	16.0
S240419-07-B	Gambusia, R2, 4/9/2024	16.2	sd=	0.32
S240419-07-C		15.6	cv=	2.0%
S240419-08-A	Mosquito Fish, B-I-Post8-MOSQ,	ND	$Mean_3$=	ND
S240419-08-B	Gambusia, Catchment, 4/9/2024	ND	sd=	-
S240419-08-C		ND	cv=	-

ND = Not Detected.
*-Value is below the method quantitation limit of 4.06 ng/g

QC Results:

ID	Theoretical Brodifacoum Concentration (ng/g)	Observed Brodifacoum Concentration (ng/g)	% Recovery
QC-09	Control	ND	N/A
QC-10	Control	ND	N/A
QC-11	12.9	13.0	101
QC-12	14.3	13.1	91.6
QC-13	490	486	99.2
QC-14	487	476	97.7

ND = Not Detected.
