# Supplementary material for: Anticoagulant residues associated with an attempted rodent eradication from a subtropical coral atoll
Source: PLoS One. 2026 Mar 23;21(3):e0344972. doi: 10.1371/journal.pone.0344972 (PMC13008109; doi:10.1371/journal.pone.0344972)
Supplement: S1 Appendix — (ZIP) [file pone.0344972.s001.zip › Supporting Information S1/24-027-3 Post 8 Midway Island Avian Liver Report.pdf]

To: Carmen Antaky
Biologist
NWRC Hawai'i Field Station

Subject: Determination of brodifacoum in avian livers matrices from Midway Island, Post 8 (QA-3404)

Methods: 188A "Determination of Multiple Rodenticide Residues in Avian Liver by dSPE and LC-MS/MS" -Non-GLP

Analysis Dates: 06/04/24, 06/05/24

Notebook References: AC169, pp.116, 135-137
QC35, p.68

Analyst: Ben Abbo

Sample Description:

Two avian liver samples were submitted on 04/19/24. See sample descriptions on p.3.

Additional Comments:

- Three replicates of each sample were analyzed, unless there was insufficient sample to analyze three replicates. The mean, standard deviation, and coefficient of variance are reported.
- The analytical run was interrupted by an instrument error during the running of these samples. The QC samples were analyzed on 06/04/24 and the samples were analyzed on 06/05/24.
- Control quail liver (S221018-03) was used as the matrix for QC samples.

Method Limit of Detection/Quantitation (MLOD/MLOQ) Values:

Method detection and quantitation limits were determined by comparing the noise at the analyte retention in three unfortified control quail liver samples to the peak height of brodifacoum in three control quail liver samples fortified to ~15 ng/g brodifacoum. The detection limit was determined to be 3X the noise and the quantitation limit was determined to be 10X the noise found in the unfortified samples.

Method Limit of Detection (MLOD)

Matrix	Detection Limit
Avian Liver	1.3 ng/g

Method Limit of Quantitation (MLOQ)

Matrix	Quantitation Limit
Avian Liver	4.26 ng/g

Results:

Sample ID	Sample Description	Brodifacoum Conc (ng/g)	Descriptive Statistics	
S240419-09-A	Myna, A-I-Post8-M, liver, Gym fig tree,	220	$Mean_3$=	226
S240419-09-B	4/4/2024	227	sd=	5.1
S240419-09-C		230	cv=	2.3%
S240419-10-A	Duck, B-I-Post8-D, liver, Radar Seep,	2.2*	$Mean_3$=	2.1*
S240419-10-B	4/2/2024	2.1*	sd=	0.058
S240419-10-C		2.1*	cv=	2.8%

ND = Not Detected.
*-Value is below the method quantitation limit of 4.26 ng/g.

QC Results:

ID	Theoretical Brodifacoum Concentration (ng/g)	Observed Brodifacoum Concentration (ng/g)	% Recovery
QC-13	Control	ND	N/A
QC-14	Control	ND	N/A
QC-15	13.7	11.5	83.9
QC-16	14.4	14.3	99.3
QC-17	500	491	98.2
QC-18	464	448	96.6

ND = Not Detected.
