# Supplementary material for: Anticoagulant residues associated with an attempted rodent eradication from a subtropical coral atoll
Source: PLoS One. 2026 Mar 23;21(3):e0344972. doi: 10.1371/journal.pone.0344972 (PMC13008109; doi:10.1371/journal.pone.0344972)
Supplement: S1 Appendix — (ZIP) [file pone.0344972.s001.zip › Supporting Information S1/24-036-3 Post 9 Midway Island Crustaceans Report.pdf]

Wildlife Services NWRC National Wildlife Research Center<br>Analytical Services Report	United States Department of Agriculture<br>Animal Plant Health Inspection Service<br>Wildlife Services<br>National Wildlife Research Center<br>Laboratory Support Services Unit	Invoice #: 24-036/3<br>Date: 07/24/24<br>Page: 1 of 3

To: Carmen Antaky
Biologist
NWRC Hawai'i Field Station

Subject: Determination of brodifacoum in crustaceans matrices from Midway Island, Post 9 (QA-3404)

Methods: 188A "Determination of Multiple Rodenticide Residues in Avian Liver by dSPE and LC-MS/MS" -Non-GLP

Analysis Dates: 07/02/24

Notebook References: AC169, pp.144-148
QC35, p.68

Analyst: Ben Abbo

Sample Description:

Three crustacean samples were submitted on 06/14/24. See sample descriptions on p.3.

Additional Comments:

- Three replicates of each sample were analyzed. The mean, standard deviation, and coefficient of variance are reported.
- Control ghost crab (S220801-42) was used as the matrix for QC samples.

Contact the author for further details on QA/QC certification at Carmen.Antaky@usda.gov

Analyst	Date	QC Specialist	Date	Reviewer	Date

Method Limit of Detection/Quantitation (MLOD/MLOQ) Values:

Method detection and quantitation limits were determined by comparing the noise at the analyte retention in three unfortified control ghost crab samples to the peak height of brodifacoum in three control ghost crab samples fortified to ~15 ng/g brodifacoum. The detection limit was determined to be 3X the noise and the quantitation limit was determined to be 10X the noise found in the unfortified samples.

Method Limit of Detection (MLOD)

Matrix	Detection Limit
Crustaceans	0.94 ng/g

Method Limit of Quantitation (MLOQ)

Matrix	Quantitation Limit
Crustaceans	3.12 ng/g

Results:

Sample ID	Sample Description	Brodifacoum Conc (ng/g)	Descriptive Statistics	
S240614-10-A	Crabs, A-I-Post9-Gh, Ghostcrabs,	ND	$Mean_3$=	ND
S240614-10-B	CargoPier, 6/6/2024	ND	sd=	-
S240614-10-C		ND	cv=	-
S240614-11-A	Crabs, B-I-Post9-Gh, Ghostcrabs, Hale	ND	$Mean_3$=	ND
S240614-11-B	Honu, 6/6/2024	ND	sd=	-
S240614-11-C		ND	cv=	-
S240614-12-A	Crabs, C-I-Post9-Gh, Ghostcrabs,	ND	$Mean_3$=	ND
S240614-12-B	RustyBucket, 6/6/2024	ND	sd=	-
S240614-12-C		ND	cv=	-

ND = Not Detected.

QC Results:

ID	Theoretical Brodifacoum Concentration (ng/g)	Observed Brodifacoum Concentration (ng/g)	% Recovery
QC-17	Control	ND	N/A
QC-18	Control	ND	N/A
QC-19	14.0	13.1	93.6
QC-20	13.0	12.6	96.9
QC-21	497	496	99.8
QC-22	456	460	101
QC-23	2060	2010	97.6
QC-24	2290	2290	100

ND = Not Detected.
