# Supplementary material for: Anticoagulant residues associated with an attempted rodent eradication from a subtropical coral atoll
Source: PLoS One. 2026 Mar 23;21(3):e0344972. doi: 10.1371/journal.pone.0344972 (PMC13008109; doi:10.1371/journal.pone.0344972)
Supplement: S1 Appendix — (ZIP) [file pone.0344972.s001.zip › Supporting Information S1/25-001 Post 10 Midway Island Avian Livers Report.pdf]

Wildlife Services **NWRC** National Wildlife Research Center<br>Analytical Services Report	United States Department of Agriculture<br>Animal Plant Health Inspection Service<br>Wildlife Services<br>National Wildlife Research Center<br>Laboratory Support Services Unit	Invoice #: 25-001/8<br>Date: 01/31/25<br>Page: 1 of 3

To:	Carmen Antaky<br>Biologist<br>NWRC Hawai'i Field Station
Subject:	Determination of brodifacoum in avian livers matrices from Midway Island, 1-Year Post (QA-3404)
Methods:	188A "Determination of Multiple Rodenticide Residues in Avian Liver by dSPE and LC-MS/MS" -Non-GLP
Analysis Dates:	01/06/25
Notebook References:	AC169, pp.177-179, 188-190<br>QC35, p.68
Analyst:	Ben Abbo

Sample Description:

Six avian liver samples were submitted on 10/10/24. See sample descriptions on p.3.

Additional Comments:

- Due to the small amount of sample available, each sample was analyzed in its entirety as a single sample. The value from these single samples is reported.
- Control quail liver (S221018-03) was used as the matrix for QC samples.

Method Limit of Detection/Quantitation (MLOD/MLOQ) Values:

Method detection and quantitation limits were determined by comparing the noise at the analyte retention in three unfortified control quail liver samples to the peak height of brodifacoum in three control quail liver samples fortified to ~15 ng/g brodifacoum. The detection limit was determined to be 3X the noise and the quantitation limit was determined to be 10X the noise found in the unfortified samples.

Method Limit of Detection (MLOD)

Matrix	Detection Limit
Avian Liver	1.3 ng/g

Method Limit of Quantitation (MLOQ)

Matrix	Quantitation Limit
Avian Liver	4.26 ng/g

Results:

Sample ID	Sample Description	Brodifacoum Conc (ng/g)
S241010-69-A	Seabird, A-I-1YearPost-Se, LAAL chick (liver), Cargo Pier, 8/27/2024	ND
S241010-70-A	Seabird, B-I-1YearPost-Se, WHTE chick (liver), Gym, 8/29/2024	ND
S241010-71-A	Duck, A-I-1YearPost-Du, LADU liver, Radar, 8/20/2024	45.8
S241010-72-A	Duck, A-II-1YearPost-Du, LADU liver, Radar, 8/21/2024	71.8
S241010-73-A	Duck, B-I-1YearPost-Du, LADU liver, Mauka Makai Ditch, 7/8/2024	354
S241010-74-A	Passerines, A-I-1YearPost-Pass, COCA liver, Greenhouse, 7/20/2024	12.3

ND = Not Detected.
INS-Insufficient Sample

QC Results:

ID	Theoretical Brodifacoum Concentration (ng/g)	Observed Brodifacoum Concentration (ng/g)	% Recovery
QC-37	Control	ND	N/A
QC-38	Control	ND	N/A
QC-39	13.1	12.7	96.9
QC-40	14.7	15.2	103
QC-41	539	518	96.1
QC-42	465	451	97.0
QC-43	2430	2390	98.4
QC-44	2340	2280	97.4

ND = Not Detected.
