# Supplementary material for: Anticoagulant residues associated with an attempted rodent eradication from a subtropical coral atoll
Source: PLoS One. 2026 Mar 23;21(3):e0344972. doi: 10.1371/journal.pone.0344972 (PMC13008109; doi:10.1371/journal.pone.0344972)
Supplement: S1 Appendix — (ZIP) [file pone.0344972.s001.zip › Supporting Information S1/25-001 Post 10 Midway Island Crustaceans Report.pdf]

To: Carmen Antaky
Biologist
NWRC Hawai'i Field Station

Subject: Determination of brodifacoum in crustaceans matrices from Midway Island, 1-Year Post (QA-3404)

Methods: 188A "Determination of Multiple Rodenticide Residues in Avian Liver by dSPE and LC-MS/MS" -Non-GLP

Analysis Dates: 01/06/25

Notebook References: AC169, pp.177-179, 189-191
QC35, p.68

Analyst: Ben Abbo

Sample Description:

Three crustacean samples were submitted on 10/10/24. See sample descriptions on p.3.

Additional Comments:

- Three replicates of each sample were analyzed. The mean, standard deviation, and coefficient of variance are reported.
- Control ghost crab (S220801-42) was used as the matrix for QC samples.

Method Limit of Detection/Quantitation (MLOD/MLOQ) Values:

Method detection and quantitation limits were determined by comparing the noise at the analyte retention in three unfortified control ghost crab samples to the peak height of brodifacoum in three control ghost crab samples fortified to ~15 ng/g brodifacoum. The detection limit was determined to be 3X the noise and the quantitation limit was determined to be 10X the noise found in the unfortified samples.

Method Limit of Detection (MLOD)

Matrix	Detection Limit
Crustaceans	0.94 ng/g

Method Limit of Quantitation (MLOQ)

Matrix	Quantitation Limit
Crustaceans	3.12 ng/g

Results:

Sample ID	Sample Description	Brodifacoum Conc (ng/g)	Descriptive Statistics	
S241010-31-A	Crabs, A-I-1YearPost-Gh, Ghostcrabs,	ND	$Mean_3$=	ND
S241010-31-B	CargoPier, 8/26/2024	ND	sd=	-
S241010-31-C		ND	cv=	-
S241010-32-A	Crabs, B-I-1YearPost-Gh, Ghostcrabs, Hale	ND	$Mean_3$=	ND
S241010-32-B	Honu, 8/26/2024	ND	sd=	-
S241010-32-C		ND	cv=	-
S241010-33-A	Crabs, C-I-1YearPost-Gh, Ghostcrabs,	ND	$Mean_3$=	ND
S241010-33-B	RustyBucket, 8/26/2024	ND	sd=	-
S241010-33-C		ND	cv=	-

ND = Not Detected.

QC Results:

ID	Theoretical Brodifacoum Concentration (ng/g)	Observed Brodifacoum Concentration (ng/g)	% Recovery
QC-29	Control	ND	N/A
QC-30	Control	ND	N/A
QC-31	14.1	14.4	102
QC-32	12.3	13.7	111
QC-33	447	455	102
QC-34	453	454	100
QC-35	2250	2210	98.2
QC-36	2170	2180	101

ND = Not Detected.
