# Supplementary material for: Anticoagulant residues associated with an attempted rodent eradication from a subtropical coral atoll
Source: PLoS One. 2026 Mar 23;21(3):e0344972. doi: 10.1371/journal.pone.0344972 (PMC13008109; doi:10.1371/journal.pone.0344972)
Supplement: S1 Appendix — (ZIP) [file pone.0344972.s001.zip › Supporting Information S1/25-001 Post 10 Midway Island Invertebrates Report.pdf]

To: Carmen Antaky
Biologist
NWRC Hawai'i Field Station

Subject: Determination of brodifacoum in invertebrate matrices from Midway Island, 1-Year Post (QA-3404)

Methods: 188A "Determination of Multiple Rodenticide Residues in Avian Liver by dSPE and LC-MS/MS" -Non-GLP

Analysis Dates: 01/10/25

Notebook References: AC169, pp.177-179, 195-197
QC35, p.68

Analyst: Ben Abbo

Sample Description:

Twelve invertebrate samples were submitted on 10/10/24. See sample descriptions on p.3.

Additional Comments:

- Three replicates of each sample were analyzed, unless there was insufficient sample to analyze three replicates. The mean, standard deviation, and coefficient of variance are reported.
- Control crickets (S221018-01) were used as the matrix for QC samples.

Method Limit of Detection/Quantitation (MLOD/MLOQ) Values:

Method detection and quantitation limits were determined by comparing the noise at the analyte retention in three unfortified control cricket samples to the peak height of brodifacoum in three control cricket samples fortified to ~15 ng/g brodifacoum. The detection limit was determined to be 3X the noise and the quantitation limit was determined to be 10X the noise found in the unfortified samples.

Method Limit of Detection (MLOD)

Matrix	Detection Limit
Invertebrates	1.2 ng/g

Method Limit of Quantitation (MLOQ)

Matrix	Quantitation Limit
Invertebrates	3.97 ng/g

Results:

Sample ID	Sample Description	Brodifacoum Conc (ng/g)	Descriptive Statistics	
S241010-01-A	Grubs, A-I-1YearPost-G, Emerald beetle,	ND	$Mean_3$=	ND
S241010-01-B	Radar, 8/26/2024	ND	sd=	-
S241010-01-C		ND	cv=	-
S241010-02-A	Grubs, B-I-1YearPost-G, Emerald beetle,	ND	$Mean_3$=	ND
S241010-02-B	Brackish, 8/26/2024	ND	sd=	-
S241010-02-C		ND	cv=	-
S241010-03-A	Grubs, C-I-1YearPost-G, Emerald beetle,	ND	$Mean_3$=	ND
S241010-03-B	RustyBucket, 8/26/2024	ND	sd=	-
S241010-03-C		ND	cv=	-
S241010-04-A	Roaches, A-I-1YearPost-R, Blattodea,	ND	$Mean_3$=	ND
S241010-04-B	Radar, 8/22/2024	ND	sd=	-
S241010-04-C		ND	cv=	-
S241010-05-A	Roaches, B-I-1YearPost-R, Blattodea,	ND	$Mean_3$=	ND
S241010-05-B	Brackish, 8/22/2024	ND	sd=	-
S241010-05-C		ND	cv=	-
S241010-06-A	Roaches, C-I-1YearPost-R, Blattodea,	ND	$Mean_3$=	ND
S241010-06-B	RustyBucket, 8/22/2024	ND	sd=	-
S241010-06-C		ND	cv=	-
S241010-07-A	Adult, A-I-1YearPost-Em, Emerald beetle,	ND	$Mean_3$=	ND
S241010-07-B	Radar, 8/27/2024	ND	sd=	-
S241010-07-C		ND	cv=	-
S241010-08-A	Adult, B-I-1YearPost-Em, Emerald beetle,	ND	$Mean_3$=	ND
S241010-08-B	Brackish, 8/27/2024	ND	sd=	-
S241010-08-C		No Sample	cv=	-
S241010-09-A	Adult, C-I-1YearPost-Em, Emerald beetle,	ND	$Mean_3$=	ND
S241010-09-B	RustyBucket, 8/27/2024	ND	sd=	-
S241010-09-C		ND	cv=	-
S241010-10-A	Pitfall, A-I-1YearPost-Pitfall, Pooled, Radar,	ND	$Mean_3$=	ND
S241010-10-B	8/27/2024	No Sample	sd=	-
S241010-10-C		No Sample	cv=	-
S241010-11-A	Pitfall, B-I-1YearPost-Pitfall, Pooled,	ND	$Mean_3$=	ND
S241010-11-B	Brackish, 8/27/2024	ND	sd=	-
S241010-11-C		ND	cv=	-
S241010-12-A	Pitfall, C-I-1YearPost-Pitfall, Pooled,	ND	$Mean_3$=	ND
S241010-12-B	RustyBucket, 8/29/2024	ND	sd=	-
S241010-12-C		No Sample	cv=	-

ND = Not Detected.
*-Value is below the method quantitation limit of 3.97 ng/g

QC Results:

ID	Theoretical Brodifacoum Concentration (ng/g)	Observed Brodifacoum Concentration (ng/g)	% Recovery
QC-61	Control	ND	N/A
QC-62	Control	ND	N/A
QC-63	14.5	16.4	113
QC-64	13.3	13.4	101
QC-65	559	538	96.2
QC-66	497	477	96.0
QC-67	2280	2250	98.7
QC-68	2330	2260	97.0

ND = Not Detected.
