# Supplementary material for: Anticoagulant residues associated with an attempted rodent eradication from a subtropical coral atoll
Source: PLoS One. 2026 Mar 23;21(3):e0344972. doi: 10.1371/journal.pone.0344972 (PMC13008109; doi:10.1371/journal.pone.0344972)
Supplement: S1 Appendix — (ZIP) [file pone.0344972.s001.zip › Supporting Information S1/25-001 Post 10 Midway Island Plants Report.pdf]

To:	Carmen Antaky<br>Biologist<br>NWRC Hawai'i Field Station
Subject:	Determination of brodifacoum in plant matrices from Midway Island, 1-Year Post (QA-3404)
Methods:	188A "Determination of Multiple Rodenticide Residues in Avian Liver by dSPE and LC-MS/MS" -Non-GLP
Analysis Dates:	01/07/25
Notebook References:	AC169, pp.177-179, 192-194<br>QC35, p.68
Analyst:	Ben Abbo

Sample Description:

Three plant samples were submitted on 10/10/24. See sample descriptions on p.3.

Additional Comments:

- Three replicates of each sample were analyzed. The mean, standard deviation, and coefficient of variance are reported.
- Control lime (S221013-01) was used as the matrix for QC samples.

Method Limit of Detection/Quantitation (MLOD/MLOQ) Values:

Method detection and quantitation limits were determined from by comparing the noise at the analyte retention in three unfortified control lime samples to the peak height of brodifacoum in three control lime samples fortified to ~15 ng/g brodifacoum. The detection limit was determined to be 3X the noise and the quantitation limit was determined to be 10X the noise found in the unfortified samples.

Method Limit of Detection (MLOD)

Matrix	Detection Limit
Plants	1.1 ng/g

Method Limit of Quantitation (MLOQ)

Matrix	Quantitation Limit
Plants	3.72 ng/g

Results:

Sample ID	Sample Description	Brodifacoum Conc (ng/g)	Descriptive Statistics	
S241010-40-A	Fruit, A-I-1YearPost-Pl, Kaffir lime leaves,	ND	$Mean_3$=	ND
S241010-40-B	Community Garden A, 8/28/2024	ND	sd=	------
S241010-40-C		ND	cv=	------
S241010-41-A	Fruit, B-I-1YearPost-Pl, Persian lime,	ND	$Mean_3$=	ND
S241010-41-B	Orchard, 8/28/2024	ND	sd=	------
S241010-41-C		ND	cv=	------
S241010-42-A	Fruit, C-I-1YearPost-Pl, Papaya, Community	ND	Mean3=	ND
S241010-42-B	Garden B, 8/28/2024	ND	sd=	------
S241010-42-C		ND	cv=	------

ND = Not Detected.

QC Results:

ID	Theoretical Brodifacoum Concentration (ng/g)	Observed Brodifacoum Concentration (ng/g)	% Recovery
QC-45	Control	ND	N/A
QC-46	Control	ND	N/A
QC-47	12.8	12.4	96.9
QC-48	13.4	11.4	85.1
QC-49	507	500	98.6
QC-50	485	480	99.0
QC-51	2390	2380	99.6
QC-52	2340	2290	97.6

ND = Not Detected.
