# Supplementary material for: Anticoagulant residues associated with an attempted rodent eradication from a subtropical coral atoll
Source: PLoS One. 2026 Mar 23;21(3):e0344972. doi: 10.1371/journal.pone.0344972 (PMC13008109; doi:10.1371/journal.pone.0344972)
Supplement: S1 Appendix — (ZIP) [file pone.0344972.s001.zip › Supporting Information S1/25-001 Post 10 Midway Island Reptiles Report.pdf]

To:	Carmen Antaky<br>Biologist<br>NWRC Hawai'i Field Station
Subject:	Determination of brodifacoum in reptile matrices from Midway Island, 1-Year Post (QA-3404)
Methods:	188A "Determination of Multiple Rodenticide Residues in Avian Liver by dSPE and LC-MS/MS" -Non-GLP
Analysis Dates:	01/07/25
Notebook References:	AC169, pp.177-179, 192-194<br>QC35, p.68
Analyst:	Ben Abbo

Sample Description:

Six gecko samples were submitted on 10/10/24. See sample descriptions on p.3.

Additional Comments:

- Three replicates of each sample were analyzed. The mean, standard deviation, and coefficient of variance are reported.
- Control gecko (S220801-47) was used as the matrix for QC samples.

Method Limit of Detection/Quantitation (MLOD/MLOQ) Values:

Method detection and quantitation limits were determined by comparing the noise at the analyte retention in three unfortified control gecko samples to the peak height of brodifacoum in three control gecko samples fortified to ~15 ng/g brodifacoum. The detection limit was determined to be 3X the noise and the quantitation limit was determined to be 10X the noise found in the unfortified samples.

Method Limit of Detection (MLOD)

Matrix	Detection Limit
Reptile	2.0 ng/g

Method Limit of Quantitation (MLOQ)

Matrix	Quantitation Limit
Reptile	6.59 ng/g

Results:

Sample ID	Sample Description	Brodifacoum Conc (ng/g)	Descriptive Statistics	
S241010-34-A	Geckos, A-I-1YearPost-Ge, Mourning gecko, Clipper, 8/27/2024	ND	$Mean_3$=	ND
S241010-34-B		ND	sd=	-
S241010-34-C		ND	cv=	-
S241010-35-A	Geckos, A-II-1YearPost-Ge, House gecko, Clipper, 8/27/2024	6.69	$Mean_3$=	6.73
S241010-35-B		7.30	sd=	0.55
S241010-35-C		6.2*	cv=	8.2%
S241010-36-A	Geckos, B-I-1YearPost-Ge, House gecko, Gym/Store, 8/27/2024	13.0	$Mean_3$=	13.4
S241010-36-B		13.8	sd=	0.40
S241010-36-C		13.5	cv=	3.0%
S241010-37-A	Geckos, B-II-1YearPost-Ge, Indo-pacific gecko, Gym/Store, 8/27/2024	ND	$Mean_3$=	ND
S241010-37-B		ND	sd=	-
S241010-37-C		ND	cv=	-
S241010-38-A	Geckos, C-I-1YearPost-Ge, House gecko, FWS Office, 8/27/2024	ND	$Mean_3$=	ND
S241010-38-B		ND	sd=	-
S241010-38-C		ND	cv=	-
S241010-39-A	Geckos, C-II-1YearPost-Ge, Mourning gecko, FWS Office, 8/27/2024	ND	$Mean_3$=	ND
S241010-39-B		ND	sd=	-
S241010-39-C		ND	cv=	-

ND = Not Detected.

QC Results:

ID	Theoretical Brodifacoum Concentration (ng/g)	Observed Brodifacoum Concentration (ng/g)	% Recovery
QC-53	Control	ND	N/A
QC-54	Control	ND	N/A
QC-55	13.0	13.0	100
QC-56	15.9	15.7	98.7
QC-57	556	547	98.4
QC-58	538	519	96.5
QC-59	2280	2250	98.7
QC-60	2150	2110	98.1

ND = Not Detected.
