# Supplementary material for: Anticoagulant residues associated with an attempted rodent eradication from a subtropical coral atoll
Source: PLoS One. 2026 Mar 23;21(3):e0344972. doi: 10.1371/journal.pone.0344972 (PMC13008109; doi:10.1371/journal.pone.0344972)
Supplement: S1 Appendix — (ZIP) [file pone.0344972.s001.zip › Supporting Information S1/25-001 Post 10 Midway Island Soils Report.pdf]

Wildlife Services **NWRC** National Wildlife Research Center<br>Analytical Services Report	United States Department of Agriculture<br>Animal Plant Health Inspection Service<br>Wildlife Services<br>National Wildlife Research Center<br>Laboratory Support Services Unit	Invoice #: 25-001/6<br>Date: 01/31/25<br>Page: 1 of 4

To:	Carmen Antaky<br>Biologist<br>NWRC Hawai'i Field Station
Subject:	Determination of brodifacoum in soil matrices from Midway Island, 1-Year Post (QA-3404)
Methods:	188A "Determination of Multiple Rodenticide Residues in Avian Liver by dSPE and LC-MS/MS" -Non-GLP
Analysis Dates:	11/22/24
Notebook References:	AC169, pp.177-182<br>QC35, p.68
Analyst:	Ben Abbo

Sample Description:

Fourteen soil samples were submitted on 10/10/24. See sample descriptions on p.3-4.

Additional Comments:

- Three replicates of each sample were analyzed. The mean, standard deviation, and coefficient of variance are reported.
- Samples were extracted on 11/21/24, stored overnight at 4°C, and analyzed on 11/22/24. Brodifacoum extracts are known to be stable at 4°C for at least a week. QC sample recoveries were all within normal parameters, indicating that the results were not impacted.
- Control soil (S220801-61) was used as the matrix for QC samples.

Method Limit of Detection/Quantitation (MLOD/MLOQ) Values:

Method detection and quantitation limits were determined by comparing the noise at the analyte retention in three unfortified control soil samples to the peak height of brodifacoum in three control soil samples fortified to ~15 ng/g brodifacoum. The detection limit was determined to be 3X the noise and the quantitation limit was determined to be 10X the noise found in the unfortified samples.

Method Limit of Detection (MLOD)

**Matrix**	**Detection Limit**
Soil	1.3 ng/g

Method Limit of Quantitation (MLOQ)

**Matrix**	**Quantitation Limit**
Soil	4.29 ng/g

Results:

Sample ID	Sample Description	Brodifacoum Conc (ng/g)	Descriptive Statistics	
S241010-43-A	Soil, A-I-1YearPost-S, Radar, 8/28/2024	ND	$Mean_3$=	ND
S241010-43-B		ND	sd=	-
S241010-43-C		ND	cv=	-
S241010-44-A	Soil, B-I-1YearPost-S, Brackish, 8/28/2024	ND	$Mean_3$=	ND
S241010-44-B		ND	sd=	-
S241010-44-C		ND	cv=	-
S241010-45-A	Soil, C-I-1YearPost-S, Community Garden , 8/28/2024	ND	$Mean_3$=	ND
S241010-45-B		ND	sd=	-
S241010-45-C		ND	cv=	-
S241010-46-A	Soil, A-I-1YearPost-S1ft, Radar, 8/28/2024	ND	$Mean_3$=	ND
S241010-46-B		ND	sd=	-
S241010-46-C		ND	cv=	-
S241010-47-A	Soil, B-I-1YearPost-S1ft, Brackish, 8/28/2024	ND	$Mean_3$=	ND
S241010-47-B		ND	sd=	-
S241010-47-C		ND	cv=	-
S241010-48-A	Soil, C-I-1YearPost-S1ft, Community Garden , 8/28/2024	ND	$Mean_3$=	ND
S241010-48-B		ND	sd=	-
S241010-48-C		ND	cv=	-
S241010-49-A	Soil, A-I-1YearPost-SS, Parade Ground, 8/28/2024	ND	$Mean_3$=	ND
S241010-49-B		ND	sd=	-
S241010-49-C		ND	cv=	-
S241010-50-A	Soil, B-I-1YearPost-SS, Orchard, 8/28/2024	ND	$Mean_3$=	ND
S241010-50-B		ND	sd=	-
S241010-50-C		ND	cv=	-
S241010-51-A	Soil, C-I-1YearPost-SS, R2, 8/28/2024	ND	$Mean_3$=	ND
S241010-51-B		ND	sd=	-
S241010-51-C		ND	cv=	-
S241010-52-A	Soil, D-I-1YearPost-SS, Ballfield, 8/28/2024	ND	$Mean_3$=	ND
S241010-52-B		ND	sd=	-
S241010-52-C		ND	cv=	-

ND = Not Detected.
*-Sample was below quantitation limit of 4.29 ng/g; result should be considered qualitative.

Results:

Sample ID	Sample Description	Brodifacoum Conc (ng/g)	Descriptive Statistics	
S241010-53-A	Soil, A-I-1YearPost-SS1ft, Parade Ground,	ND	$Mean_3$=	ND
S241010-53-B	8/28/2024	ND	sd=	-
S241010-53-C		ND	cv=	-
S241010-54-A	Soil, B-I-1YearPost-SS1ft, Orchard,	ND	$Mean_3$=	ND
S241010-54-B	8/28/2024	ND	sd=	-
S241010-54-C		ND	cv=	-
S241010-55-A	Soil, C-I-1YearPost-SS1ft, R2, 8/28/2024	ND	$Mean_3$=	ND
S241010-55-B		ND	sd=	-
S241010-55-C		ND	cv=	-
S241010-56-A	Soil, D-I-1YearPost-SS1ft, Ballfield,	ND	$Mean_3$=	ND
S241010-56-B	8/28/2024	ND	sd=	-
S241010-56-C		ND	cv=	-

ND = Not Detected.
*-Sample was below quantitation limit of 4.29 ng/g; result should be considered qualitative.

QC Results:

ID	Theoretical Brodifacoum Concentration (ng/g)	Observed Brodifacoum Concentration (ng/g)	% Recovery
QC-01	Control	ND	N/A
QC-02	Control	ND	N/A
QC-03	Control	ND	N/A
QC-04	15.4	12.4	80.5
QC-05	14.5	13.6	93.8
QC-06	15.5	14.9	96.1
QC-07	516	521	101
QC-08	504	495	98.2
QC-09	542	518	95.6
QC-10	2170	2160	99.5
QC-11	2200	2180	99.1
QC-12	2100	2090	99.5

ND = Not Detected.
