# Supplementary material for: Anticoagulant residues associated with an attempted rodent eradication from a subtropical coral atoll
Source: PLoS One. 2026 Mar 23;21(3):e0344972. doi: 10.1371/journal.pone.0344972 (PMC13008109; doi:10.1371/journal.pone.0344972)
Supplement: S1 Appendix — (ZIP) [file pone.0344972.s001.zip › Supporting Information S1/25-001 Post 10 Midway Island Waters Report.pdf]

| | |
|---|---|
| To: | Carmen Antaky<br>Biologist<br>NWRC Hawai'i Field Station |
| Subject: | Determination of brodifacoum in Waters from Midway Island, 1-Year Post (QA-3404) |
| Methods: | "Determination of Brodifacoum Residues in Water"-Non-GLP |
| Analysis Dates: | 12/19/24, 12/20/24 |
| Notebook References: | AC169, pp.177-179, 187-188<br>QC35, p.68 |
| Analyst: | Ben Abbo |

---

**Sample Description:**

Twelve samples of water from Midway Atoll were submitted on 10/10/24. See sample descriptions on pp.3-4.

---

**Additional Comments:**

- Sea water samples were analyzed on 12/19/24. Fresh water samples were analyzed on 12/20/24.
- Two replicates of each sample were analyzed.
- Cache la Poudre River water from Laporte, Colorado (S241212-01) was used as the matrix for QC samples for fresh water.
- Synthetic sea water (NWRC ID# 7262) was used as the matrix for QC samples for sea water.

**Sample Preparation and Extraction:**

Water Extraction Procedure:

1. Add 60 mL of water sample to a 125-mL separatory funnel.
2. Add 0.020 mL surrogate to all and 0.040 mL of acetonitrile or 75X brodifacoum stock as indicated.
3. Add 20 mL chloroform.
4. Add ~8.5g sodium chloride.
5. Add 10 mL 1M hydrochloric acid.
6. Cap and shake for 8-10s, let set 1 minute. Repeat 2X.
7. Dispense chloroform phase into 25-mL glass tube taking care to not transfer any water phase.
8. Remove solvent in a 60°C N-Evap with a gentle flow of nitrogen gas.
9. Add 0.300 mL acetonitrile, vortex thoroughly, wetting as much of the inside surface of the tube as possible.
10. Add 1.200 mL pH 9.5 20-mM ammonium acetate buffer and vortex thoroughly.
11. Transfer the sample to an autosampler vial and assay by LC-MS/MS.

**Method Limit of Detection/Quantitation (MLOD/MLOQ) Values:**

Method detection and quantitation limits were determined by comparing the noise at the analyte retention in two unfortified control water samples to the peak height of brodifacoum in two control water samples fortified to 0.0219 ng/mL brodifacoum. The detection limit was determined to be 3X the noise and the quantitation limit was determined to be 10X the noise found in the unfortified samples.

Method Limit of Detection (MLOD)

| **Matrix** | **Detection Limit** |
|---|---|
| Fresh water | 0.0028 ng/mL |
| Sea water | 0.0032 ng/mL |

Method Limit of Quantitation (MLOQ)

| **Matrix** | **Quantitation Limit** |
|---|---|
| Fresh water | 0.00938 ng/mL |
| Sea water | 0.0107 ng/mL |

**Results:**

**Fresh Water**

| Sample ID | Sample Description | Brodifacoum (ng/g) | Descriptive Statistics | |
|---|---|---|---|---|
| S241010-57-A | Drinking water, A-I-1YearPost-DW, | ND | $Avg_2$= | ND |
| S241010-57-B | FWS Office, 8/28/2024 | ND | sd= | - |
| | | | cv= | - |
| S241010-58-A | Drainage Water, A-I-1YearPost-WC, | ND | $Avg_2$= | ND |
| S241010-58-B | Catchment, 8/22/2024 | ND | sd= | - |
| | | | cv= | - |
| S241010-59-A | Drainage Water, B-I-1YearPost-WC, | ND | $Avg_2$= | ND |
| S241010-59-B | Bowling Alley, 8/28/2024 | ND | sd= | - |
| | | | cv= | - |
| S241010-64-A | Fresh Water, A-I-1YearPost-Fr, Radar, | ND | $Avg_2$= | ND |
| S241010-64-B | 8/27/2024 | ND | sd= | - |
| | | | cv= | - |
| S241010-65-A | Fresh Water, B-I-1YearPost-Fr, Brackish, | ND | $Avg_2$= | ND |
| S241010-65-B | 8/27/2024 | ND | sd= | - |
| | | | cv= | - |
| S241010-66-A | Fresh Water, C-I-1YearPost-Fr, R2, | ND | $Avg_2$= | ND |
| S241010-66-B | 8/22/2024 | ND | sd= | - |
| | | | cv= | - |
| S241010-67-A | Fresh Water, D-I-1YearPost-Fr, Drainage | ND | $Avg_2$= | ND |
| S241010-67-B | Pond, 8/22/2024 | ND | sd= | - |
| | | | cv= | - |
| S241010-68-A | Fresh Water, E-I-1YearPost-Fr, Office | ND | $Avg_2$= | ND |
| S241010-68-B | Guzzler, 8/27/2024 | ND | sd= | - |
| | | | cv= | - |

ND-Not Detected
*-Sample is below the quantitation limit o 0.00938 ng/mL

**Sea Water**

| Sample ID | Sample Description | Brodifacoum (ng/g) | Descriptive Statistics | |
|---|---|---|---|---|
| S241010-60-A | Ocean Water, A-I-1YearPost-Oc, CargoPier, | ND | $Avg_2$= | ND |
| S241010-60-B | 8/23/2024 | ND | sd= | - |
| | | | cv= | - |
| S241010-61-A | Ocean Water, B-I-1YearPost-Oc, Hale Honu, | ND | $Avg_2$= | ND |
| S241010-61-B | 8/23/2024 | ND | sd= | - |
| | | | cv= | - |
| S241010-62-A | Ocean Water, C-I-1YearPost-Oc, | ND | $Avg_2$= | ND |
| S241010-62-B | RustyBucket, 8/23/2024 | ND | sd= | - |
| | | | cv= | - |
| S241010-63-A | Ocean Water, D-I-1YearPost-Oc, Harbor, | ND | $Avg_2$= | ND |
| S241010-63-B | 8/26/2024 | ND | sd= | - |
| | | | cv= | - |

ND-Not Detected

**QC Results:**

**Fresh Water:**

| ID | Theoretical Brodifacoum Concentration (ng/mL) | Observed Brodifacoum Concentration (ng/mL) | % Recovery |
|---|---|---|---|
| QC-21 | Control | ND | N/A |
| QC-22 | Control | ND | N/A |
| QC-23 | 0.0219 | 0.0230 | 105 |
| QC-24 | 0.0219 | 0.0232 | 106 |
| QC-25 | 1.77 | 1.71 | 96.6 |
| QC-26 | 1.77 | 1.74 | 98.3 |
| QC-27 | 5.32 | 5.22 | 98.1 |
| QC-28 | 5.32 | 5.79 | 109 |

ND-Not Detected

**Sea Water:**

| ID | Theoretical Brodifacoum Concentration (ng/mL) | Observed Brodifacoum Concentration (ng/mL) | % Recovery |
|---|---|---|---|
| QC-13 | Control | ND | N/A |
| QC-14 | Control | ND | N/A |
| QC-15 | 0.0219 | 0.0209 | 95.4 |
| QC-16 | 0.0219 | 0.0182 | 83.1 |
| QC-17 | 1.77 | 1.74 | 98.3 |
| QC-18 | 1.77 | 1.77 | 100 |
| QC-19 | 5.32 | 5.22 | 98.1 |
| QC-20 | 5.32 | 5.15 | 96.8 |

ND-Not Detected
